# Site-Specific Modification of the Anticancer and Antituberculosis Polyether Salinomycin by Biosynthetic Engineering

**DOI:** 10.1002/cbic.201402300

**Published:** 2014-08-22

**Authors:** Hanna Luhavaya, Simon R Williams, Hui Hong, Luciana Gonzaga de Oliveira, Peter F Leadlay

**Affiliations:** [a[ Department of Biochemistry, University of Cambridge80 Tennis Court Road, Cambridge CB2 1GA (UK); [b[ University Chemical Laboratory, University of CambridgeLensfield Road, Cambridge CB2 1EW (UK); [c[ Department of Organic Chemistry, University of Campinas, UNICAMP, Cidade Universitária Zeferino Vaz s/nP.O. Box 6154, 13083-970, Campinas, SP (Brazil)

**Keywords:** biosynthesis, dehydratases, ionophores, polyketides, spiroacetals

## Abstract

The complex bis-spiroacetal polyether ionophore salinomycin has been identified as a uniquely selective agent against cancer stem cells and is also strikingly effective in an animal model of latent tuberculosis. The basis for these important activities is unknown. We show here that deletion of the *salE* gene abolishes salinomycin production and yields two new analogues, in both of which the C18=C19 *cis* double bond is replaced by a hydroxy group stereospecifically located at C19, but which differ from each other in the configuration of the bis-spiroacetal. These results identify SalE as a novel dehydratase and demonstrate that biosynthetic engineering can be used to redirect the reaction cascade of oxidative cyclization to yield new salinomycin analogues for use in mechanism-of-action studies.

Natural products provide both valuable leads for drug discovery and chemical probes for fundamental biological mechanisms. There is therefore continuing interest in their site-specific chemical modification.[1] An alternative approach to conventional chemical modification is the genetic engineering of biosynthetic pathways to produce novel analogues of natural product drugs.[2] In particular, the modular polyketide synthase (PKS) multienzymes that govern the synthesis of bacterial complex polyketides follow a remarkable assembly-line model, in which the growing polyketide or peptide chain is handed on from one set (or module) of enzymes to the next, each module catalysing a single round of chain extension with use of short carboxylic acid building blocks.[3] The full-length chain then undergoes further chemical transformation by additional enzymes. Genetic manipulation of these pathways has been successfully applied to produce useful libraries of bacterial complex polyketides, such as the immunosuppressants rapamycin[4] and FK506.[5] However, extension of this attractive engineering approach to many other such natural products has not proved routine, because the limits to the modularity of the synthases and the specificity of auxiliary enzymes are poorly understood.

We[6] and others[7] have characterized the gene cluster that governs the biosynthetic pathway to salinomycin (**1**), 
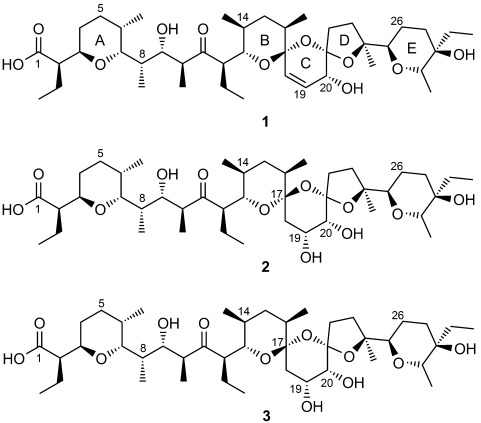
a polyether ionophore antibiotic from *Streptomyces albus* that is used in animal husbandry to combat parasitic infections. Commercial fermentation of salinomycin is highly productive, reaching yields of up to 100 g L^−1^. Its severe toxicity has prevented clinical use, but salinomycin has surprisingly emerged from a functional screen of 16 000 compounds as a novel anticancer agent uniquely selective in its toxicity towards cancer stem cells (CSCs).[8] CSCs cause treatment failure through resistance to clinically used anticancer drugs and radiation therapy.[9] Subsequent work has highlighted additional antitumour activities.[10] Salinomycin is also effective in an animal model for latent tuberculosis (TB),[11] which afflicts roughly one third of humanity.

The detailed mechanisms of these effects are unknown, but they are likely to be linked to salinomycin's function as an ionophore.[12] Analogues of salinomycin that retained the key structural features and cation-binding properties of salinomycin but showed lower toxicity would therefore be valuable for structure–activity studies to establish the mechanisms of CSC and TB cytotoxicity, and might eventually open the way to targeted therapies. The total synthesis of salinomycins has been achieved in several laboratories[13] and remains an option for making analogues, despite the challenge of establishing the correct stereochemistry of its remarkable 1,6,8-trioxadispiro[4.1.5.3]pentadec-13-ene core. Meanwhile, conventional chemical modification at C1[14] and at C20[15] of salinomycin has revealed analogues with significantly lower toxicity towards normal cell lines and higher antiproliferative activity against drug-resistant cancer cell lines than salinomycin itself. However, most areas of the molecule remain inaccessible to medicinal chemistry. We describe here an investigation into the enzymology of the late stages of salinomycin biosynthesis to uncover the origin of the unusual *cis* double bond at C18–C19 within the bis-spiroacetal core. This has resulted in salinomycin derivatives that can be produced by direct fermentation.

Salinomycin is formed in *S. albus* by initial assembly-line polyketide chain synthesis on a modular polyketide synthase[6] (Figure S2 in the Supporting Information). As for all ionophoric polyethers patterned on monensin,[16] oxidative cyclization is initiated on the enzyme-tethered polyketide by epoxidation of precisely placed carbon–carbon double bonds catalysed by a flavin-linked epoxidase (here, SalC), and completed by a cascade of epoxide ring-opening and polyether ring formation catalysed by novel cyclase enzymes (here, some combination of SalBI, SalBII, and SalBIII).[6] The Δ*salC* mutant of *S. albus* accumulated a diene shunt metabolite[6] in which the only ring already formed was pyran ring A. Also, the unusual *cis* double bond at C18=C19 was absent. This focussed our attention on the protein product of the unassigned (but essential[7]) gene *salE*, which bears a remote sequence similarity to authentic dehydratases (Figure S3), as a candidate enzyme responsible for catalysing formation of the *cis* double bond during oxidative cyclization. We thought it possible that the deletion mutant Δ*salE* of *S. albus* might accumulate a metabolite bearing 1,2-diol functionality at C19/C20, which would open up new possibilities for medicinal chemistry on this important molecule. This would fit the previous observation of a species with *m*/*z* 791.5 after deletion of this gene.[7] We show here that strains in which *salE* is deleted from the salinomycin cluster actually produce two major components, E15 (**2**) and E16 (**3**), the structures of which confirm the predicted site-specific alteration in salinomycin.

To facilitate analysis of the role of SalE, and future manipulation of salinomycin biosynthesis, we undertook transplantation of the entire salinomycin gene cluster (Figures S18 and S19) as a single 136.8 kbp region of *S. albus* DNA into *Streptomyces coelicolor* M1154, a heterologous host strain producing no significant polyketide natural products of its own.[17] A large-insert P1-based artificial chromosome (PAC) library in *Escherichia coli* was screened for the presence of the *sal* genes, and one positive PAC clone was selected, characterized and transferred by conjugation into *S. coelicolor* M1154 (Supporting Information and Figure S20).[18] HPLC-MS analysis of the recombinant strain confirmed the production of authentic salinomycin at levels (≈2.4 mg mL^−1^) around 10 % of those typically seen from wild-type *S. albus* (Figure 1). This result provides direct evidence that the cluster had been transplanted intact and that the *sal* biosynthetic genes are appropriately expressed in the heterologous host. To the best of our knowledge this is the first example of heterologous expression of a polyether biosynthetic gene cluster.

**Figure 1:**
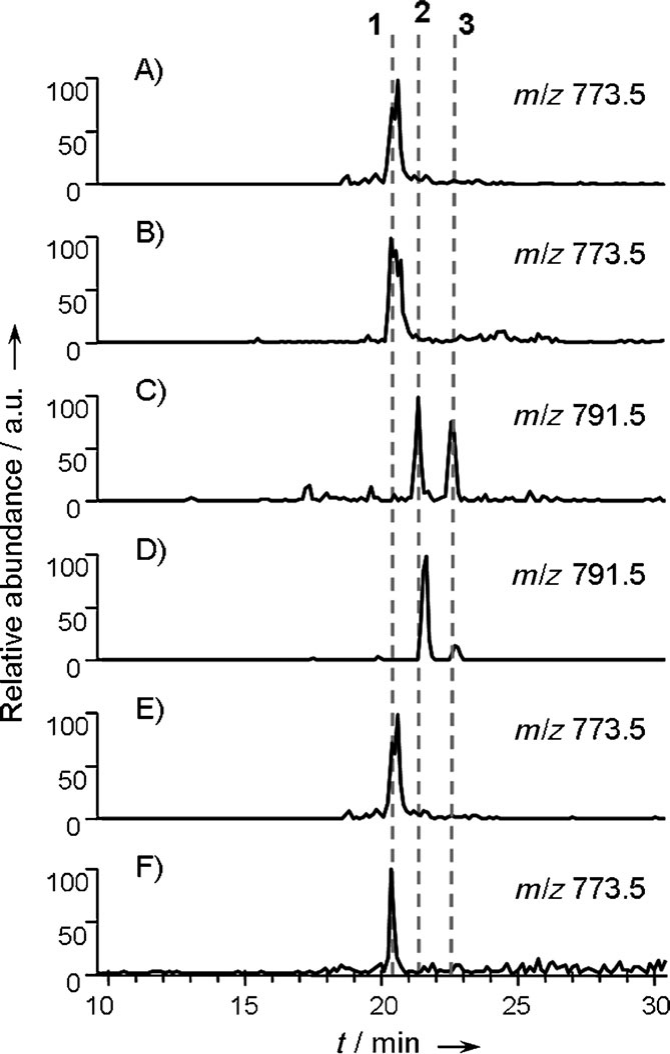
Salinomycins produced by biosynthetic engineering. A) Compound 1 produced in the heterologous host strain *S. coelicolor* M1154. B) Compound 1 produced in the wild-type strain *S. albus*. C) Compounds 2 and 3 produced in *S. albus* Δ*salE* mutant. D) Compounds 2 and 3 produced from the modified *sal* gene cluster expressed in *S. coelicolor* M1154. E) Production of 1 was restored in *S. albus* (Δ*salE*) strain complemented with expression plasmid pIB-*salE*. F) Salinomycin sodium salt standard.

We carried out the targeted in-frame deletion of the *salE* gene in the salinomycin-producing strain of *S. albus* and checked the identity of the mutant *S. albus* (Δ*salE*) by PCR analysis and Southern hybridization (Figures S5 and S6). Salinomycin production in this mutant was abolished, but production was completely restored by complementation with a copy of *salE* housed on an integrative plasmid (Figure S8). In parallel, the *salE* gene in the PAC clone housing the *sal* gene cluster was specifically replaced by a gene conferring apramycin resistance. When this altered gene cluster was transferred to *S. coelicolor* and expressed, salinomycin production was likewise abolished (Supporting Information and Figure S24). Analysis of culture extracts from *S. albus* (Δ*salE*) by HPLC-MS revealed the presence of two new peaks at *m*/*z* 791.5 corresponding to the molecular ions of compounds **2** and **3** (Figure 1).The same two new peaks were seen in culture extracts from *S. coelicolor* M1154 (PAC-Δ*salE*), with compound **2** as the more abundant component (Figure 1).

Salinomycin analogues **2** (8 mg) and **3** (4 mg) were purified from 6 L of culture of the *S. albus* (Δ*salE*) mutant by reversed-phase HPLC (Supporting Information and Figure S9). HRMS (ESI) analysis confirmed that they share the same molecular formula (C_42_H_72_O_12_Na), and both LCMS (ESI) and high-resolution MS^*n*^ analysis (Figures S11–S16) gave results fully consistent with the proposed stereoisomeric structures, containing 1,2-diol functionality at C19/C20. The structures of **2** and **3** were then fully established by comprehensive analysis of one- and two-dimensional NMR spectroscopic data and detailed comparison with salinomycin (**1**). NOESY (Figure 2) and ^1^H–^1^H coupling constant data were used to determine the stereochemistry of the hydroxy group at C19 and the configuration of the bis-spiroacetal region formed by the BCD rings (supplementary NMR analysis). We initially thought that **2** and **3** might differ in the configuration of the hydroxy group inserted at C20 because if the cytochrome P450 hydroxylase SalD were confronted with a non-native substrate this enzyme might no longer discriminate between the two C=H bonds at this position. Surprisingly, the NMR analysis revealed that **2** and **3** are both *cis*-(19*R*,20*R*)-1,2-diols. They differ from each other only in the stereochemistry of the bis-spiroacetal, with **3** having the same configuration as in salinomycin at C17 and C21, whereas **2** has the opposite configuration at C17, and in this stereoisomer the ring C adopts a twisted boat conformation (Figure 2 and supplementary NMR analysis). The *R* configuration determined for the hydroxy group at C19 that is installed during polyketide chain synthesis is in full agreement with the predicted stereochemistry of ketoreduction by the ketoreductase in extension module 6 of the salinomycin PKS[6] (Figures S2 and S4). In some culture extracts of *S. albus* (Δ*salE*) we also detected an additional salinomycin-related metabolite with *m*/*z* 775.5, although in amounts too small for NMR analysis. This metabolite was not present in extracts from *S. coelicolor* M1154 (Figure S26). HRMS (ESI) of this metabolite gave the molecular formula C_42_H_72_O_11_Na, and its high-resolution MS^2^ fragmentation pattern (Figure S29) is fully consistent with the structures of 20-deoxy-**2** or 20-deoxy-**3**, thus hinting that P450 hydroxylation at this position is not essential for bis-spiroacetal formation.

**Figure 2:**
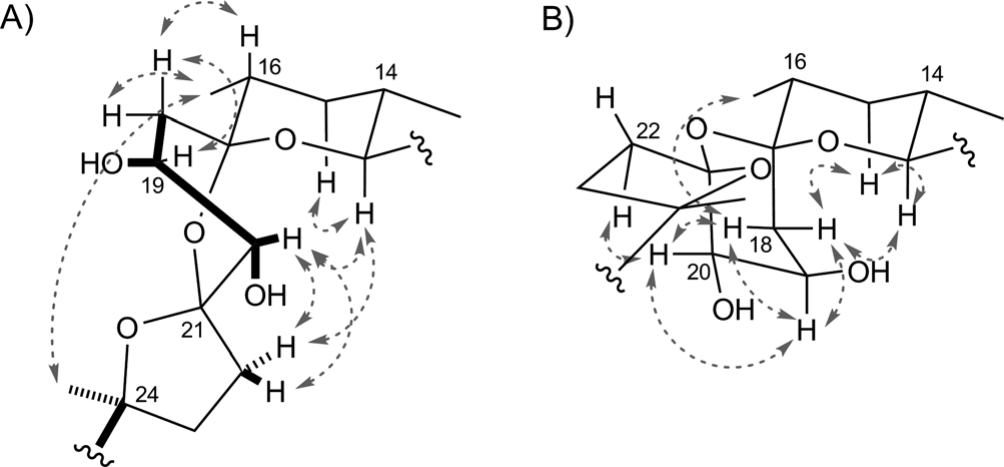
NMR analysis of engineered salinomycins. A) Structure of the bis-spiroacetal core in 2, consisting of rings B, C and D, determined as described in the supplementary NMR analysis. B) Structure of the bis-spiroacetal core in 3 (supplementary NMR analysis). Both compounds contain a *cis*-(19*R*,20*R*)-1,2-diol. Compound 3 has the same bis-spiroacetal stereostructure as in salinomycin. However, 2 is epimeric at C17, and ring C adopts a twisted boat conformation in this isomer. Key NOE correlations are shown by arrows.

A tentative scheme to account for the formation of **2** and **3** in the absence of the putative dehydratase SalE is shown in Scheme 1. The polyketide chain undergoes all late-stage enzymatic modifications while it is still tethered either to the last *sal* PKS extension module at the C terminus of SalAIX or to an auxiliary enzyme.[6] Stereospecific spiroketal formation in certain actinomycete polyketides—the antibiotics avermectin[19] and reveromycin, for example[20]—proceeds by dehydrative cyclization of a dihydroxyketone precursor catalysed by a spirocyclase. However, in polyether ionophores such as salinomycin the trigger for spiroacetal formation is the ring opening of epoxide intermediates, catalysed by new epoxide hydrolase/cyclase enzymes of the MonB[16] family, and neither the *sal* gene cluster nor any other cluster for a polyether ionophore encodes an enzyme with sequence similarity to known spirocyclases.[19] The observation that the P450 hydroxylase SalD shows the same regio- and stereospecificity in the absence of SalE as in the normal pathway to salinomycin makes it likely that SalD acts before spiroketal formation is made irreversible by epoxide ring opening catalysed by the SalB epoxide hydrolases (Scheme 1). SalE also probably acts before epoxide ring opening, but the exact timing of dehydration with respect to C20 hydroxylation remains to be established. Various factors might govern the observed ratio of **2** to **3**. In many spiroacetals the configuration at the spiroacetal centre is determined by contributions from anomeric effects, dipole–dipole interactions, opportunities for intramolecular hydrogen-bonding and steric effects.[20] In the salinomycin system there is also a potential contribution from metal binding to the nascent chain, and it cannot be discounted that the specificity and kinetics of subsequent chain release affect the observed product ratios. Also, previous work on the biosynthesis of the polyether lasalocid[21] has shown that oxidative cyclization and spiroacetal formation (as well as other enzymatic “late” steps) might actually take place during enzyme-bound extension of the polyketide chain rather than on the full-length chain. Other features of this remarkable enzymatic reaction cascade, such as the mechanism of formation of tetrohydropyran ring A, remain to be defined. In certain other polyethers such rings are generated by intramolecular oxaconjugate addition onto a 2-enoyl thioester, catalysed by a specific pyran synthase (PS), but the salinomycin gene cluster does not apparently encode a PS homologue.[22] The mechanism of final polyketide chain release in salinomycin biosynthesis is also unknown at present. Nevertheless, recognition of the role of SalE encourages the view that similar investigation of the remaining unannotated enzymes encoded in the gene cluster will allow deconvolution of these steps. Meanwhile, it is clear that the approach of “biosynthetic medicinal chemistry”[23] can be extended even to this highly complex polyether.

**Scheme 1:**
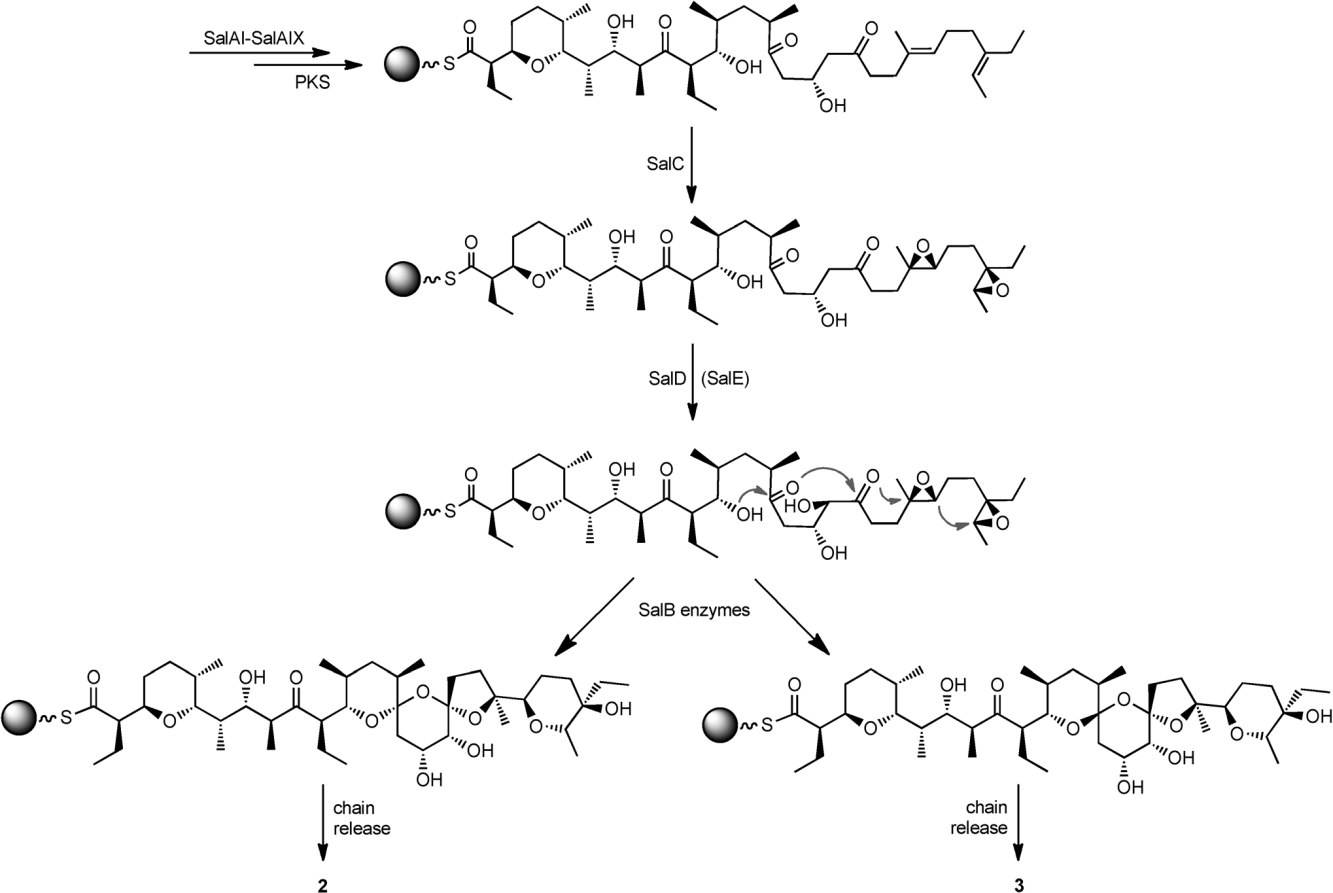
Biosynthesis of engineered salinomycins. The point at which it is proposed that the dehydratase SalE acts in the normal biosynthetic pathway to salinomycin is indicated. When SalE is missing, 2 and 3 are produced. Other enzymes shown as involved at each stage are discussed in the text. Enzyme attachment is indicated by the shaded sphere.
