# Supplementary material for: Site-Specific Modification of the Anticancer and Antituberculosis Polyether Salinomycin by Biosynthetic Engineering
Source: Chembiochem. 2014 Aug 22;15(14):2081–5. doi: 10.1002/cbic.201402300 (PMC4515104; doi:10.1002/cbic.201402300)
Supplement: Supplementary file 1 [file cbic0015-2081-sd1.pdf]

CHEMBIOCHEM

Supporting Information

© Copyright Wiley-VCH Verlag GmbH & Co. KGaA, 69451 Weinheim, 2014

Site-Specific Modification of the Anticancer and Antituberculosis Polyether Salinomycin by Biosynthetic Engineering

Hanna Luhavaya,[a] Simon R. Williams,[b] Hui Hong,[a] Luciana Gonzaga de Oliveira,[c] and Peter F. Leadlay*[a]

cbic_201402300_sm_miscellaneous_information.pdf

Table of Contents

1. Supplementary Methods……………………………………………………………………………3
1.1. General analytical procedures…………………………………………………………………......3
1.2. Bacterial strains and culture conditions………………………………………………………………4
1.3 Materials, DNA isolation and manipulation……………………………………………………......5
1.4. Culture extraction for HPLC-MS analysis of metabolites…………………………………………..6
1.5. Purification of *S. albus* Δ*salE* metabolites – **2** and **3**……………………………………………………6
1.6 Gene disruption in *S. albus* DSM 41398………………………………………………………...6
1.7. Southern hybridisation…………………………………………………………………………………7
1.8. Construction of a plasmid for complementation…………………………………………………...7
1.9. Heterologous expression of salinomycin in *S. coelicolor* M1154…………………… ………………….8
1.10. *E. coli* triparental mating and conjugation of *S. coelicolor* M1154……………….............................8
1.11 *salE* gene inactivation in *E. coli*……..…………..............9
2. Supplementary Figures…………………………………………………………………………11
Figure S1. The organisation of the biosynthetic gene cluster for salinomycin……….………………………11
Figure S2. Proposed mechanism of polyketide chain assembly on the salinomycin PKS…………………….12
Figure S3. Alignment of the SalE with authentic dehydratases – FabA and FabZ……………………………13
Figure S4. Alignment of ketoreductase domains from the salinomycin PKS…………………………………14
Figure S5. Schematic illustration of in-frame deletion of the *salE* gene..……………….……..…………..14
Figure S6. PCR and Southern blot confirmation of in-frame deletion of the *salE* gene …………………......15
Figure S7. Restriction analysis of the complementation plasmid pIB-*salE*…………..……………………......15
Figure S8. LC-ESI-MS analysis of metabolite profile of *S. albus* strains……...……………………….……16
Figure S9. LC-ESI-MS analysis of purified compounds **2** and **3**………………...........................…..16
Figure S10. High-resolution MS analysis of compounds **2** and **3**…………………………………………17
Figure S11. High-resolution MS/MS spectrum of compound **2** ………………………………………...17
Figure S12. High-resolution MS/MS spectrum of compound **3** …………..……………………………………17
Figure S13. Proposed fragmentation pathways for compounds **2** and **3** based on MS^n analysis……………...18
Figure S14. High-resolution MS^3 spectrum for m/z 549.3 for **2** and **3**……………………..........................19
Figure S15. High-resolution MS^3 spectrum for m/z 449.3 for **2** and **3**……………………………………..19
Figure S16. High-resolution MS^3 spectrum for m/z 535.3 for **2** and **3**……………………………………..20
Figure S17. Comparison of MS spectra of non-deuterated and deuterated ions for compounds **2** and **3**……..20
Figure S18. Heterologous expression of salinomycin biosynthetic gene cluster……………………………...21
Figure S19. The map of the PAC vector containing the salinomycin biosynthetic gene cluster……………... 22
Figure S20. Confirmation of integration of the *sal* gene cluster in the *S. coelicolor* M1154 genome...……....22
Figure S21. ESI-MS/MS analysis of **1** produced by *S. coelicolor* and *S. albus* strains ……………..……..23
Figure S22. Gene inactivation in PAC DNA using λ-Red recombination ……..………..…………………..23
Figure S23. Confirmation of the replacement of the *salE* gene in PAC-*sal* by the *acc(3)IV* gene conferring apramycin resistance…………………………………………………………………………..………………24

Figure S24. LC-ESI-MS analysis of the metabolite profile of *S. albus* Δ*salE* and *S. coelicolor* M1154:PAC-Δ*salE* ……………………………………………………………………………………………..24

Figure S25. ESI-MS/MS analysis of **2** and **3** produced by *S. coelicolor* M1154:PAC-Δ*salE* and *S. albus* Δ*salE* strains ……………………………………………………………………………………………25

Figure S26. LC-ESI-MS analysis of the metabolite with m/z 775.5……..……...………..…………………...25

Figure S27. MS/MS spectrum of $[M+Na]^+$ m/z 775.5…………………………...………..……………..26

Figure S28. MS/MS spectrum of $[M+Na]^+$ m/z 775.5 (expansion)…………………...………..…………..26

Figure S29. High-resolution MS/MS spectrum of $[M+Na]^+$ m/z 775.5 ……….……………………...26

3. Supplementary Tables…………………………………………………………………………27

Table S1. Bacterial strains used in this study……………………………………...............................27

Table S2. Plasmids used in this work……………………………......……………....…………………...28

Table S3. Oligonucleotide primers used in this work………………………………...............................29

Table S4. Deduced function of genes of salinomycin cluster…………………………………………………29

Table S5. MS data of **2** and **3** measured by HR-ESI-Orbitrap MS…………………...............................30

Table S6. MS/MS data of **2** measured by HR-ESI-Orbitrap MS…………………...............................31

Table S7. MS/MS data of **3** measured by HR-ESI-Orbitrap MS…………………...............................31

Table S8. MS/MS data of $[M+Na]^+$ m/z 775.5 measured by HR-ESI-Orbitrap MS………...........................32

4. NMR Analysis…………………………………………………………………………………...33

Figure S30. Proposed 2D structure for **2** and **3** showing spin systems and key HMBC correlations…...…...33

Figure S31. NMR analysis of *bis*-spiroacetal ring system in **3**…………………...……………..……34

Figure S32. NMR analysis of *bis*-spiroacetal ring system in **2**…………………...……………..……35

Figure S33. Conformation of the A ring…………………………...………………………………36

Figure S34. Conformation of the E ring ……………………………….. ……………….........................37

Figure S35. NOE correlations and coupling constants in the C7-C10 region…….………............................38

Figure S36-50. NMR spectra for **2**………………………………………………………………………42

Figure S51-60. NMR spectra for **3**………………………………………………………………………57

Figure S61-70. NMR spectra for **1**………………………………………………………………………67

Table S9. Selected coupling constants in CD_3CN in the A ring of **1**, **2**, **3**……………………………..…..37

Table S10. Selected coupling constants in CD_3CN in the C7-C10 region of **1**, **2**, **3**………………….…....38

Table S11. NMR data for **2**……………………..……...……………………...............................39

Table S12. NMR data for **3**………………………………...…………………..…...........................40

Table S13. NMR data for **1**………………………………....……………………...............................41

5. Supplementary References…………………………………………………..........................77

1. Supplementary Methods

1.1. General analytical procedures

NMR data were collected using Bruker Avance spectrometers using either a 500 DCH cryoprobe operating at 500.05 MHz for ^{1}H and 125.7 MHz for ^{13}C (^{13}C, DEPT, TOCSY) or a 500 TCI cryoprobe operating at 500.13 MHz for ^{1}H and 125.8 MHz for ^{13}C (^{1}H, DQF-COSY, HSQC, HSQC-TOCSY, HMBC, NOESY) in the Chemistry Department, University of Cambridge. Chemical shifts were recorded using an internal deuterium lock for ^{13}C and residual ^{1}H in CD_3CN (δ_H 1.94, δ_C 118.26) or CD_3OD (δ_H 3.31, δ_C 49.00), and are given in ppm on a scale relative to $\delta_{TMS} = 0$. NMR spectra were processed using Bruker Topspin (v. 3.2).

DQF-COSY spectra were acquired with 2k data points in F_2 and 360 increments with 2 scans per increment. TOCSY spectra were acquired using DIPSI2 modulation and a mixing time of 120 ms, 8k data points were acquired in F_2 and 360 increments with 2 scans per increment. The edited HSQC spectra were optimized for 145 Hz with 2k data points in F_2, 256 increments and 2 scans per increment. HMBC spectra optimized for 8 Hz with a three-fold low pass J filter to suppress one bond couplings, 4k data points were acquired in F_2 with 360 increments and 16 scans per increment. Edited-HSQC-TOCSY spectra were optimized for 145 Hz using DIPSI2 modulation and a mixing time of 120 ms, 8k data points were acquired in F_2, 360 increments and 8 scans per increment. NOESY spectra were recorded using a mixing time of 0.5 s, 4k data points in F_2 with 512 increments and 16 scans per increment. All data were zero filled to 1K in F_1 for processing.

HPLC-MS analysis was performed using an HPLC (Hewlett Packard, Agilent Technologies 1100 series) coupled to a Finnigan MAT LCQ mass spectrometer fitted with an electrospray ionization (ESI) source. The HPLC was fitted with a Prodigy 5µ C18 column (4.6 × 250 mm, Phenomenex) column. A solvent system of CH_3OH and H_2O both containing 0.1% formic acid (v/v) was used. Samples were eluted with a linear gradient of 85 to 100% of CH_3OH over 20 min, then 100% CH_3OH over 10 min at a flow rate 0.7 mL min^{-1} (method A). Alternatively a linear gradient of 85 to 100% of CH_3OH over 15 min, then 100% CH_3OH over 8 min at a flow rate 1 mL min^{-1} was used (method B). The mass spectrometer was run in positive ionization mode, scanning from m/z 150 to 1800, and the collision energy was set to 35%. Production of salinomycin (**1**), E15 (**2**), and E16 (**3**) metabolites was verified by LC-MS^2 analysis on $[M+Na]^+$ ions at m/z 773.5, 791.5 and 791.5 respectively with a normalized collision energy of 35%.

ESI high resolution MS (ESI-HR-MS) was carried out on a Thermo Fisher Orbitrap with 60,000 resolution and normalized collision energy of 15%.

HPLC-MS data were processed and deconvoluted using Xcalibur (v. 1.1) (Thermo Finnigan).

Preparative HPLC purification was performed using a Synergi C18 column (4 μM, 2.0 × 150 mm, Phenomenex), and using 20 mM ammonium acetate and MeOH as solvents. Samples were eluted with a gradient of 85 to 99% MeOH over 40 min at a flow rate of 15 $mLmin^{-1}$.

DNA sequencing was carried out by the DNA Sequencing Facility in the Department of Biochemistry, University of Cambridge.

1.2. Bacterial strains and culture conditions

Streptomyces albus strains were grown in TSBY liquid medium (3% tryptone soy broth, 10.3% sucrose, 0.5% yeast extract) for isolation of genomic DNA, and on SFM solid medium (2% mannitol, 2% soya flour, 2% agar) for conjugation and strain maintenance. For liquid cultures, the strains were grown at 30°C with shaking at 220 rpm in a rotary incubator for 36-44 h. For solid culture, the strains were grown at 30°C for 10-12 days.

For salinomycin production, seed medium (sucrose 4%, soybean flour 1%, brewer's yeast 0.5%, $CaCO_3$ 0.2%, pH 7.5) was inoculated with *S. albus* spore suspension and cultured for 36 - 48 h. 10% inocula of seed culture were used for fermentation medium (soybean flour 1%, starch 0.5%, $CaCO_3$ 0.5%, $(NH_4)_2SO_4$ 0.3%, NaCl 0.2%, $MgSO_4$ 0.01%, KH_2PO_4 0.02%, pH 7.5, after autoclaving sunflower oil was added at final level 6%). Fermentation was carried out at 30°C and 240 rpm in a rotary incubator for 7 days. Not more than 40 mL of culture was grown in each 250 mL flasks without metal spring.

To check salinomycin production in *Streptomyces coelicolor* M1154 the strain was grown at 30°C for 7 days on 35 ml R2YE agar plates inoculated with 100 μL from 2-day-old TSBY seed cultures (R2YE agar: sucrose 103 g, K_2SO_4 0.25 g, $MgCl_2·6H_2O$ 10.12 g, glucose 10 g, Difco casaminoacids 0.1 g, distilled H_2O 800 mL, Bacto agar 2.2 g for 80 mL of solution; add just before use to 80 mL of solution: KH_2PO_4, 0.5% - 1mL, $CaCl_2·2H_2O$, 3.68% - 8 ml, L-proline, 20% - 1.5 mL, TES buffer, 5.73% pH7.2 - 10 mL, trace element solution - 0.2 mL, NaOH 1N - 0.5 mL, Difco yeast extract 10% - 5 mL; trace element solution (per 1L): $ZnCl_2$ - 40 mg, $FeCl_3·6H_2O$ - 200 mg, $CuCl_2·2H_2O$ - 10 mg, $MnCl_2·4H_2O$ - 10 mg, $Na_2B_4O_7·10H_2O$ - 10 mg, $(NH_4)_6Mo_7O_{24}·4H_2O$ - 10 mg).

E. coli strains containing PAC DNA were grown in Luria-Bertani (LB) broth (1% tryptone, 0.5% yeast extract, 1% NaCl) or on LB agar (1% tryptone, 0.5% yeast extract, 1% NaCl, 2% agar) at 37°C with appropriate antibiotics selection (apramycin 50 μg mL^{-1}, chloramphenicol 25 μg mL^{-1}, kanamycin 50 μg mL^{-1}, carbanecillin 50 μg mL^{-1}). For cloning and conjugation purposes of *E. coli* without PAC DNA 2TY medium (1.6% tryptone, 1% yeast extract, 0.5% NaCl) was used with appropriate antibiotic selection (apramycin 50 μg mL^{-1}, chloramphenicol 25 μg mL^{-1}, kanamycin 50 μg mL^{-1}, ampicillin 100 μg mL^{-1}).

1.3. Materials, DNA isolation and manipulation

Bacterial strains, plasmids and oligonucleotides (Invitrogen) used in this work are summarised in Tables S1, S2 and S3 respectively. Restriction endonucleases and alkaline phosphatase were purchased from New England Biolabs. T4 DNA ligase was purchased from Fermentas. All chemicals were from Sigma-Aldrich. All organic solvents used were HPLC grade.

Plasmid DNA was isolated from an overnight culture using the Plasmid Mini Kit I (Omega BioTek) according to the manufacturer's protocol.

High-molecular weight PAC DNA was isolated from *E. coli* overnight culture by alkaline lysis.[1]

High molecular weight genomic DNA from *Streptomyces* strains was isolated using the salting out procedure.[2]

Purification of DNA fragments from agarose gels was performed using the Anachem Gel Recovery Kit according to the manufacturer's instructions.

PCR amplifications were carried out using a Mastercycler (Eppendorf) and the *Phusion* High-Fidelity PCR Master Mix from New England Biolabs (for cloning), or BioMix Red from Bioline (for screening purposes).

Reaction mixture A was used for amplifying fragments for gene knockouts, reaction B for gene knockout confirmation and PAC library screening, and reaction C for *salE* gene amplification.

PCR reaction mixture:	A	B	C
Milli-Q Water	10.5 µL	7.1 µL	15 µL
Primer 1 (15 pM)	2 µL	0.8 µL	2 µL
Primer 2 (15 pM)	2 µL	0.8 µL	2 µL
DMSO	5 µL	0.4 µL	5 µL
$MgCl_2$ (50 mM)	5 µL	0.4 µL	–
Template DNA	0.5 µL	0.5 µL	1 µL
2x Polymerase Mix	25 µL	10 µL	25 µL
Total volume	50 µL	20 µL	50 µL

PCR reaction conditions (30 cycles) for A and B reactions

Initial denaturation	98°C	30 s
Denaturation	98°C	10 s
Annealing	72→66°C (interval 0.2°C)	30 s
Extension	72°C	1 min
Final extension	72°C	5 min
	4°C	∞

PCR reaction conditions (30 cycles) for C reactions

Initial denaturation	98°C	30 s
Denaturation	98°C	10 s
Annealing	64 °C	30 s
Extension	72°C	45 s
Final extension	72°C	5 min
	4°C	∞

1.4. Culture extraction for HPLC-MS analysis of metabolites

500 μL samples of culture broth of *S. albus* strains were extracted with 500 μL of ethyl acetate. The solvent was evaporated, the residue was redissolved in 500 μL of methanol and the mixture centrifuged before being subjected to HPLC-MS analysis. 5 μL of the solution was injected.

For analysis of *S. coelicolor* M1154 metabolite profiles a 35 mL R2YE agar plate was extracted twice with 35 mL of ethyl acetate. The organic phase was evaporated, the residue was dissolved in 1 mL of methanol, the mixture was centrifuged, and 5 μL of the supernatant was analyzed by HPLC-MS.

1.5. Purification of *S. albus* Δ*salE* metabolites 2 and 3

A 7-day-old 6 L culture broth of *S. albus* DSM 41398 Δ*salE* mutant strain was extracted twice with 4 L of ethyl acetate. The combined organic layers were dried over $MgSO_4$ and the solvent was evaporated, yielding 153 g of an oily residue. The latter was redissolved in 2 L of hexane and extracted three times with 1 L methanol/water (4:1, v/v) mixture. Methanol-water fractions were combined, and methanol was removed by evaporation at reduced pressure. The remaining water layer was extracted three times with 500 mL of ethyl acetate. The combined extracts were dried with anhydrous $MgSO_4$ and the solvent was removed in vacuo. 8.4 g of oily residue was obtained. To remove the remaining oil, the sample was further purified by flash chromatography on a column (3 cm × 20 cm), of silica gel, 60 μm particle size. The column was washed with 300 mL of 1:1 (v/v) mixture of hexane/ethyl acetate, and compounds eluted with 2 L of ethyl acetate/methanol (19:1, v/v). Combined fractions contained 1.4 g of a mixture containing compounds **2** and **3**. Further purification was achieved by repeated rounds of preparative HPLC; fractions were collected at 0.9 min intervals. Final fractions were combined and desalted using Chromabond C18 EC column (Macherey-Nagel) yielding around 8 mg of **2** and 4 mg of **3** (Figure S9). All stages of purification were monitored by direct injection into the Finnigan MAT LCQ mass spectrometer or by HPLC-MS analysis.

1.6. Gene disruption in *S. albus* DSM 41398

In-frame deletion or replacement of the *salE* gene was carried out as follows. Recombinant plasmids were constructed by ligating DNA fragments (about 2 kb) PCR-amplified from the upstream and downstream flanks of the target gene into vector pYH7, previously digested with *NdeI* and gel purified. To ligate the fragments the isothermal Gibson assembly method was used as described.[3] The assembly mixture was incubated at 50°C for 60 min, and then was used to transform *E. coli* DH10B.

To disrupt *salE* by in-frame deletion, two flanking fragments to be used for homologous recombination were amplified from *S. albus* genomic DNA (gDNA) by PCR using the following two pairs of primers: SalE_1F, SalE_1R and SalE_2F, SalE_2R. The integrity of recombinant plasmids was checked by restriction digestion and sequencing. To verify the in-frame deletion in the construct and in the mutant by PCR, a pair of primers PCR_salE_f and PCR_salE_r was designed. The approach is schematically depicted in Figure S5.

The construct obtained, pHLΔ*salE*, was introduced by conjugation into *S. albus* DSM 41398. The donor strain was *E. coli* ET12657/pUZ8002, and conjugation was carried out on 25 mL SFM plates. After incubation at 30°C for 20 hours, exconjugants were selected with 5 µg mL^{-1} apramycin and 25 µg mL^{-1} nalidixic acid. Exconjugants were transferred to an SFM plate containing 50 µg mL^{-1} apramycin and 25 µg mL^{-1} nalidixic acid to double check for antibiotic resistance. Loss of recombinant plasmid pHLΔ*salE* with consequent formation of potential double-crossover mutants was initiated by streaking exconjugants on SFM agar medium for up to 12 rounds of non-selective growth. Single colonies were patched onto both SFM agar and SFM agar containing apramycin (50 µg mL^{-1}) in parallel to check for apramycin sensitivity; colonies with the correct phenotype (Apr^S) were selected and further grown in TSBY medium, gDNA was purified. Potential mutants were checked by PCR and Southern blot analysis (Figure S6).

1.7. Southern hybridisation

7 µg of genomic DNA from *S. albus* WT and *S. albus* Δ*salE* strains was digested for four hours with *PvuII*. After separation by agarose gel electrophoresis, *PvuII*-digested *S. albus* WT and Δ*salE* gDNA were probed, using the Roche DIG system according to the manufacturer's instructions, with a DIG-labelled 3654 bp fragment recovered from pHLΔ*salE* plasmid by digestion with *NdeI*. pHLΔ*salE* plasmid digested with *NdeI* used as a reference in gel electrophoresis alongside *S. albus* WT and Δ*salE* gDNA.

1.8. Construction of a plasmid for complementation

The complementation plasmid pIB-*salE* was constructed based upon integrative vector pIB139 placing *salE* under the *ermE** promoter. The PCR product (primers salE_NdeI_F and salE_EcoRV_R) and the pIB139 vector were digested with *NdeI* and *EcoRV* and the target fragments were recovered from a 0.7% agarose gel. The ligation of the digested and purified PCR product with the vector (treated with alkaline phosphatase after restriction step) was performed using T4 DNA ligase followed by transformation into DH10B competent cells using heat shock at 42°C for 55 sec. Cells were transferred onto LB plates containing 50 µg mL^{-1} apramycin. After incubation overnight, transformants were picked and inoculated into 10 mL LB broth containing

50 µg mL^{-1} apramycin and incubated at 37°C, with shaking at 250 rpm for 16 h. Plasmids were isolated and their identity confirmed by restriction analysis and DNA sequencing (Figure S7). pIB-*salE* was integrated into the *S. albus* Δ*salE* mutant by conjugation as described above.

1.9. Heterologous expression of salinomycin in *S. coelicolor* M1154

Heterologous production of salinomycin was carried out by expression of the entire *sal* biosynthetic gene cluster in *S. coelicolor* M1154. To capture the entire biosynthetic gene cluster a PAC library was constructed from genomic DNA of *S. albus* DSM 41398.

Mycelia of *S. albus* DSM 41398 were prepared for PAC library construction by growing the strain in 30 mL of TSBY medium inoculated with mycelia from TSBY starter culture. The mycelia were pelleted by centrifugation (4,600 × *g*, 10 min) and washed three times with SET buffer (75 mM NaCl, 20 mM Tris-HCl (pH 7.2), 75 mM EDTA (pH 8.0)), and flash-frozen in liquid nitrogen before shipping in dry ice to Canada. Large genomic DNA fragments were ligated into pESAC13 vector between two *BamHI* sites. The genomic PAC library of *S. albus* DSM 41398 was constructed by Bio S&T Inc. (Montreal, Canada). The library comprised 2,304 individual clones, which represented more than 20 times genomic coverage, and was constructed in *E. coli* DH10B host. Based on 14 randomly selected clones, the average size of an insert was 139 kb and only 1/14 clones contained no insert.

The screening of the library was performed as follows. Individual PAC clones were grown in 96 deep-well plates at 37°C, 300 rpm overnight. For each row (8 wells), the cultures (800 µL) were pooled into a 15-ml plastic centrifuge tube (Greiner) and centrifuged (4,600 × *g*, 10 min, 4°C). After DNA purification by alkaline lysis PCR reactions with primer pairs (salPAC cen_F, salPAC cen_R) complementary to the centre region of the salinomycin biosynthetic cluster were performed. Those samples that gave a band of the correct size were subjected to another round of PCR – with 2 primer pairs, complementary to the very beginning (salPAC beg_F, salPAC beg_R) and very end (salPAC end_F, salPAC end_R) regions of the cluster. Clones corresponding to the positive hits were grown again, DNA was isolated from each individual clone and subjected to PCR analysis with beginning and end primer pairs together. One out of the positive clones – PAC-*sal* -was chosen for further sequencing analysis to define exact insert boundaries and for heterologous expression experiments. The sequencing results showed that the exact insert size is 136,770 bp (Figure S19).

1.10. *E. coli* triparental mating and conjugation of *S. coelicolor* M1154

This is a two-step protocol for transferring individual PAC clones (derivatives of pESAC13 vector) into the *S. coelicolor* strain[4] (Figure S18a).

<u>Step 1:</u> moving PAC clones from *E. coli* DH10B into *E. coli* ET12567.

E. coli cells ET12567 (Cam^R), TOPO10/pR9604 (Carb^R), DH10B/PAC (Kan^R) were inoculated into 5 mL LB medium containing appropriate antibiotic and incubated overnight at 37°C, 250 rpm. From the overnight culture 500 μL was inoculated into 10 mL LB medium containing half of the working concentration of appropriate antibiotic and incubated at 37°C, 250 rpm until A_{600} reached 0.4. The cells were harvested by centrifugation at 2,200 × *g* for 5 min and washed twice with 20 mL of LB medium. The supernatant was discarded and the pellet was resuspended in 500 μL of LB medium. 20 μL of each strain was dripped onto the same location on the LB agar plate lacking antibiotics so that the three strains were mixed together. After drying the plates were incubated at 37°C overnight for tri-parental conjugation. Next day, to select for *E. coli* ET12567 derivatives containing the PAC clone and the helper plasmid (pR9604) the cells from the spot were streaked onto fresh LB agar plates containing kanamycin, chloramphenicol and carbanicillin antibiotics and incubated at 37°C overnight. Single colonies were used to inoculate LB medium containing the antibiotics. PCR analysis was carried out to confirm the presence of the PAC clone in ET12567 cells.

Step 2: conjugation between *E. coli* ET12567 containing pESAC13 derivatives and the helper plasmid (pR9604), and *S. coelicolor* M1154 strain.

The standard conjugation protocol was followed.[2] Mixtures of *Streptomyces* and *E. coli* were plated on SFM agar plates, and overlaid after 20 h with thiostrepton (50 μg mL^{-1}) and nalidixic acid (25 μg mL^{-1}). After observing putative exconjugants they were streaked onto an SFM plate containing thiostrepton (50 μg mL^{-1}) and nalidixic acid (25 μg mL^{-1}). Thiostrepton resistant colonies were grown in TSBY medium for genomic DNA purification and PCR analysis to confirm that the entire PAC clone has been transferred to the *S. coelicolor* recipient (Figure S20). Integration of an empty pESAC13 vector was done in parallel as a negative control.

1.11. *salE* gene inactivation in *E. coli*

To inactivate the *salE* gene in *E. coli* the λ-RED recombination approach was used (Figure S22).

The vector pIJ790 was transformed into *E. coli* DH10B with PAC clone PAC-*sal* and the cells were grown under chloramphenicol and kanamycin selection at 30°C. A single colony was grown overnight at 30°C in 5 mL of LB medium supplemented with the same antibiotics. 10 mL of fresh LB medium containing chloramphenicol and kanamycin was inoculated with 100 μL of overnight culture and grown at 30°C to OD_{600} reached 0.3. After 0.1% of L-arabinose was added to induce expression of *exo, bet, gam* and the cells were grown at 37°C for 45 min. Cultures then were centrifuged and electrocompetent *E. coli* cells (DH10B/PAC) were prepared as described by Dower.[5]

Linear DNA fragments containing *acc(3)IV* gene PCR were amplified from plasmid pIJ773 using primer pairs salE_to_Apr cas_PAC_F and salE_to_Apr cas_PAC_R to knockout *salE* gene. *E. coli* DH10B/PAC were electroporated with 100 ng of the linear fragment. For electroporation, the mixture of *E. coli* cells with DNA was immediately transferred into an ice-cold 2 mm electroporation cuvette and electroporated at 2.5 kV (25 μF, 200 Ω, t_{const} ~ 5 ms) using a Bio-Rad Gene Pulser II. LB medium (750 μL) was added and the cells were incubated at 37°C, 250 rpm for an hour. The culture (100 μL) was streaked onto LB plate containing apramycin and kanamycin and incubated overnight at 37°C. Verification of positive transformants was performed using primers PCR salE_f and PCR salE_r (Figure S23).

When apramycin-resistant clone PAC-Δ*salE* was introduced in *S. coelicolor* M1154 the plates after conjugation were overlaid with apramycin (50 μg mL^{-1}) and nalidixic acid (25 μg mL^{-1}).

2. Supplementary Figures

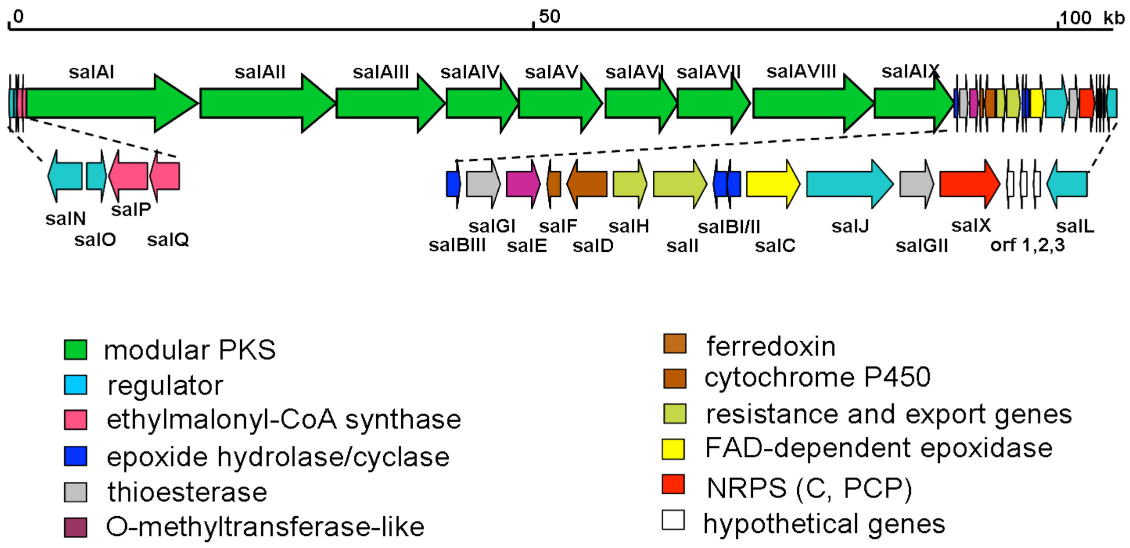

Figure S1. The organization of the biosynthetic gene cluster for salinomycin in *S. albus* DSM 41398 (not to scale) and assignment of putative gene functions.[6]

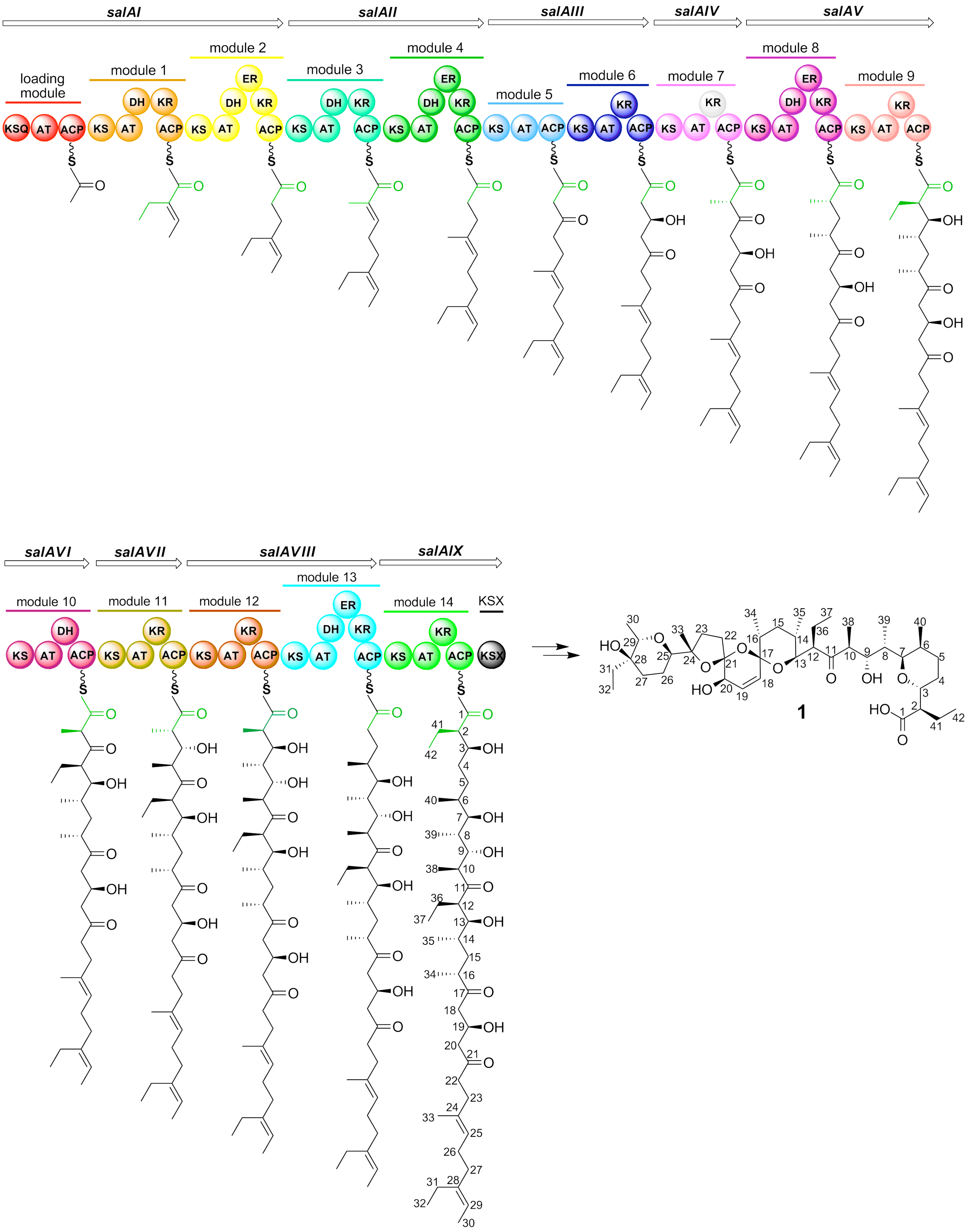

Figure S2. Proposed mechanism of polyketide chain assembly on the salinomycin PKS. The light grey KR domain in module 7 is inactive.[6]

```
                           10        20        30        40        50        60
                            |         |         |         |         |         |
FabA_E.coli        ------------------MVDKRESYTKEDLLASGRGELFGAKGPQLPAPNMLMMDRVVK
FabA_Haemophilus   --------------MNTCTPNIKSSYDYNDLLASGRGELFGSEGPQLPAPSMLMMDRITK
FabA_Pseudomonas   -------------------MTKQHAFTREDLLRCSRGELFGPGNAQLPAPNMLMIDRIVH
FabA_Azotobacter   -------------------MTKQHAYTREDLLRCARGELFGPGNAQLPAPNMLMVDRITH
SalE               METKPAQFTAETENLLIALYSRAHDAKSAKPILNDRMAVTVSESIEYDYAKLKIRDDDAI
FabZ_Haemophilus   ------------------------MAIETAENRTPKVIEVTEIMNMLPHRYPFLLVDRVT
FabZ_E.coli        ------------------------------MTTNTHTLQIEEILELLPHRFPFLLVDRVL
FabZ_Pseudomonas   ------------------------------------MMDINEIREYLPHRYPFLLVDRVV

                           70        80        90       100       110       120
                            |         |         |       * |         | *       |
FabA_E.coli        MTETGGNFDKGYVEAELDINPDLWFF--------GCHFIGDPVMPGCLGLDAMWQLVGFY
FabA_Haemophilus   ITDKEGLFEKGYIEAELDINQDLPFF--------GCHFIGDPVMPGCLGLDAMWQLVGFY
FabA_Pseudomonas   ISDVGGKYGKGELVAELDINPDLWFF--------ACHFEGDPVMPGCLGLDAMWQLVGFY
FabA_Azotobacter   ISDVGGKYGKGEMVAELDINPDLWFF--------ACHFEGDPVMPGCLGLDAMWQLVGFY
SalE               SLAIRGELIDGWIRSFLDAHPDAVVLHLGTGLDGRVHRMDPPASVEWYDLD-LPEIAALY
FabZ_Haemophilus   DYE-EGK----WLRAIKNVTVNEPCF--------TGHFPSSPVFPGVLILEAMAQATGVL
FabZ_E.coli        DFE-EGR----FLRAVKNVSVNEPFF--------QGHFPGKPIFPGVLILEAMAQATGIL
FabZ_Pseudomonas   ELDIEGK----RIRAYKNVSINEPFF--------NGHFPEHPIMPGVLIIEAMAQAAGIL

                          130       140       150       160       170       180
                            |         |         |         |         |         |
FabA_E.coli        LGWLGGEGKGRALGVG--EVKFTGQVLPTAKKVTYRIHFKRIVNRRLIMGLADGEVLV--
FabA_Haemophilus   LGWVGGKGKGRALGVG--EVKFTGQILPTAKKVIYRINMKRVINRKLVMGLADGEVEV--
FabA_Pseudomonas   LGWQGNPGRGRALGSG--EVKFFGQVLPTAKKVTYNIHIKRTINRSLVLAIADGTVSV--
FabA_Azotobacter   LGWQGNPGRGRALGSG--EVKFFGQVLPTAKKLTYNIHIKRTISRSLILGIADGTVSV--
SalE               AQLFPEREHHHVIGSSVMDLKWLEQVPTDRPTVIVAEGLLVYFTEEEVKGLIRKLFSVFP
FABZ_Haemophilus   AVATYG----KMREDELYYFAAIDNARFKRPVVPGDQLVLEVEFLKEIRGITKFTGKAYV
FabZ_E.coli        AFKSVG----KLEPGELYYFAGIDEARFKRPVVPGDQMIMEVTFEKTRRGLTRFKGVALV
FabZ_Pseudomonas   GFKMLDV---KPADGTLYYFVGSDKLRFRQPVLPGDQLQLHAKFISVKRSIWKFDCHATV

                          190       200       210       220       230       240
                            |         |         |         |         |         |
FabA_E.coli        DGRLIYTA-SDLKVGLFQDTSAF-------------------------------------
FabA_Haemophilus   DGRIIYTA-TDLKVGLFQDTTSF-------------------------------------
FabA_Pseudomonas   DGREIYSA-EGLRVGLFTSTDSF-------------------------------------
FabA_Azotobacter   DGREIYSA-EGLRVGLFTSTDSF-------------------------------------
SalE               SGQLIFDAYTPFSVKAMNKHWTVKASGKRFVWGLAEPASLEQWHPGVKHHDAWTFFGSPY
FabZ_Haemophilus   NGKLACEADLMCARK---------------------------------------------
FabZ_E.coli        DGKVVCEATMMCARSREA------------------------------------------
FabZ_Pseudomonas   DDKPVCSAEIICAERKL-------------------------------------------

                          250       260       270
                            |         |         |
FabA_E.coli        --------------------------------
FabA_Haemophilus   --------------------------------
FabA_Pseudomonas   --------------------------------
FabA_Azotobacter   --------------------------------
SalE               IAHMPWTSRVVCKIMNAIPALRHYNRVMRYSF
FabZ_Haemophilus   --------------------------------
FabZ_E.coli        --------------------------------
FabZ_Pseudomonas   --------------------------------
```

Figure S3. Alignment of the SalE with authentic dehydratases FabA and FabZ. Residues common to either FabA or FabZ are marked in yellow. Two proposed catalytic residues - His and Asp (in FabA) or Glu (in FabB) are marked with an asterisk.

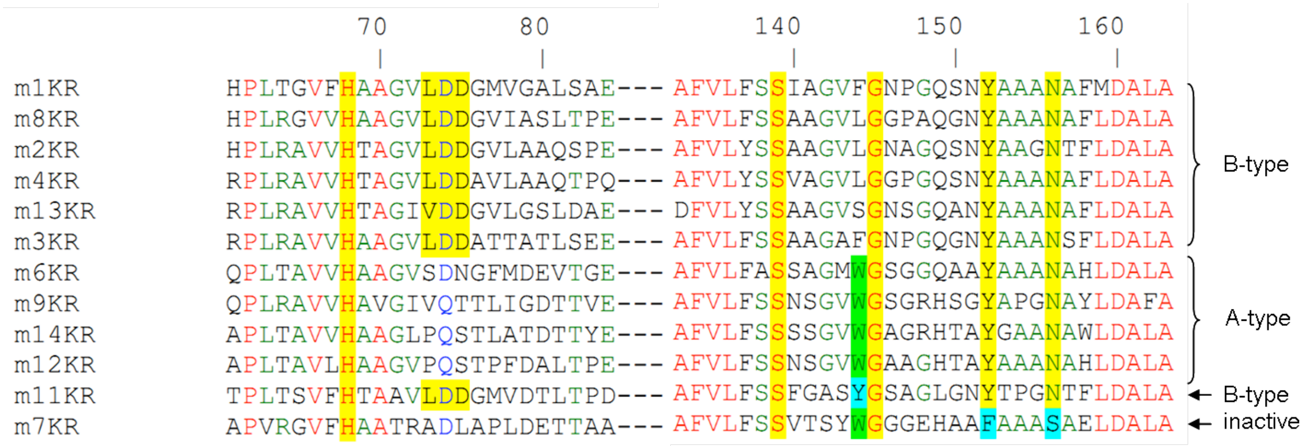

Figure S4. Alignment of the ketoreductase (KR) domains from the salinomycin PKS. Modules 6, 9, 12, 14 contain A-type KR domain which introduce *S* chirality at the corresponding C-OH; while modules 1, 2, 3, 4, 8, 11, 13 contain B-type KR domain which introduce *R* chirality at the corresponding C-OH. Stereochemistry of C19-OH is under control of KR from module 6, which gives configuration in agreement with the NMR results, which reveal (19*R*, 20*R*)-configuration in 1,2-diols **2** and **3**.

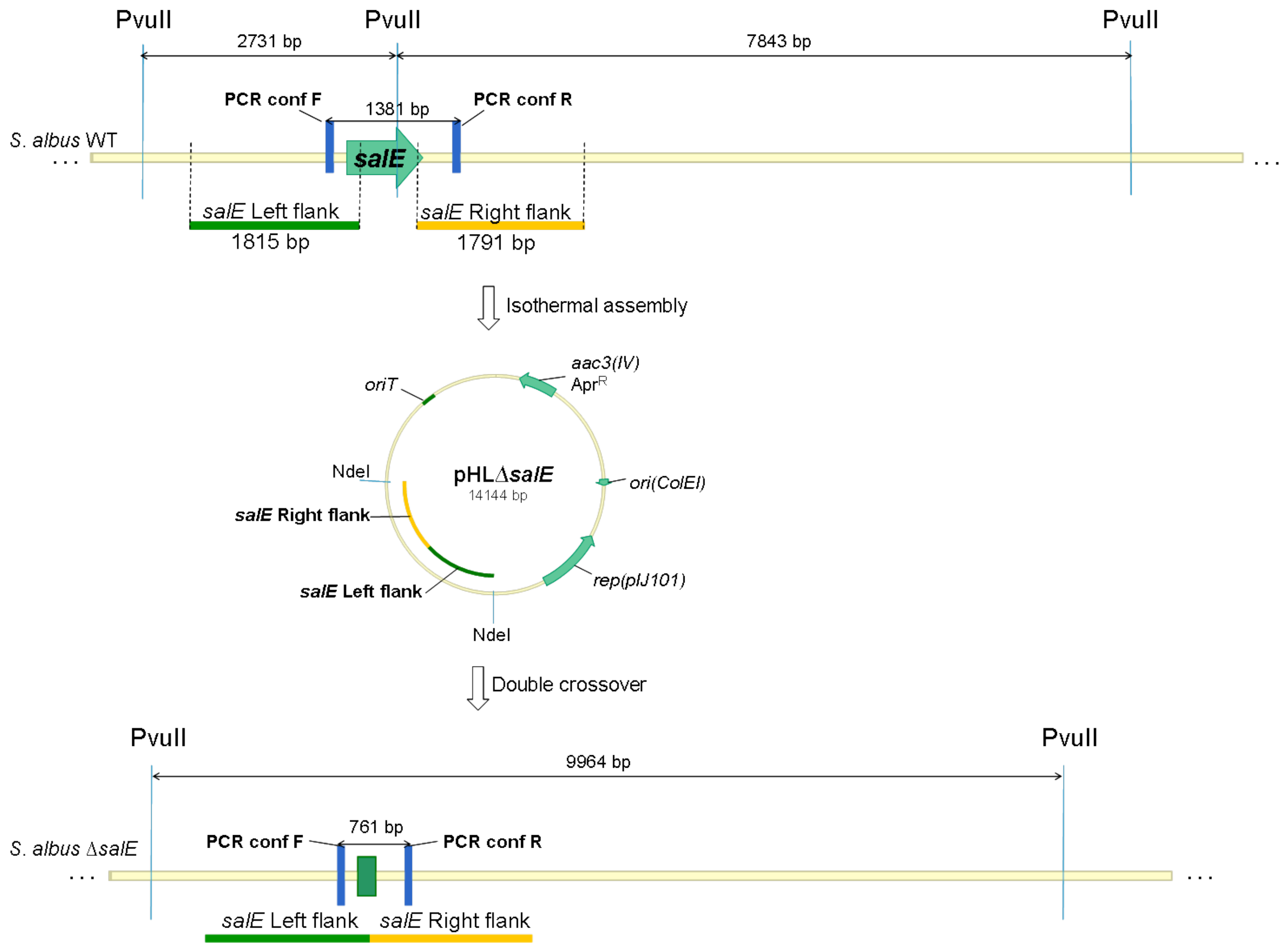

Figure S5. Schematic illustration of the in-frame deletion of the *salE* gene in the salinomycin biosynthetic gene cluster.

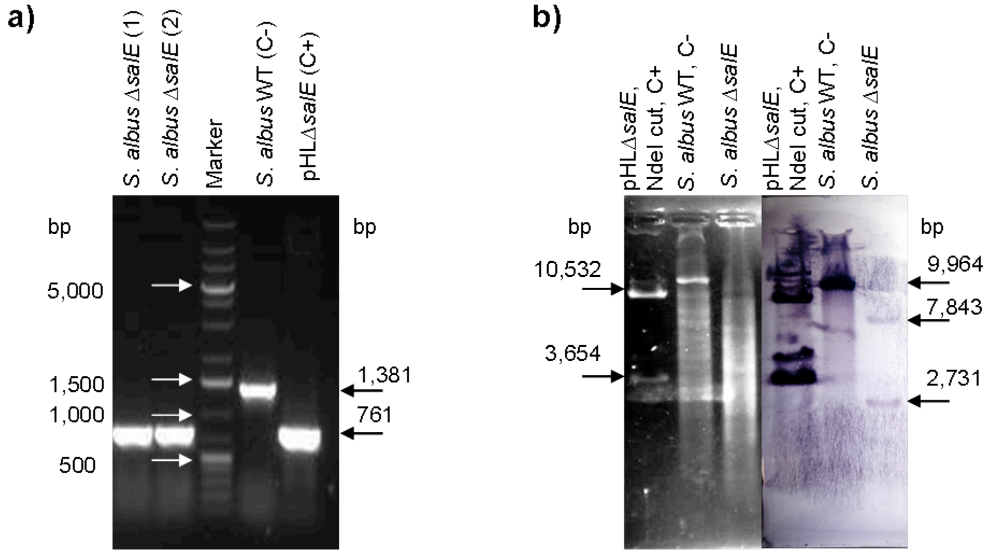

Figure S6. In-frame deletion of *salE* in the salinomycin biosynthetic gene cluster. (**a**) PCR analysis. In the case of the *salE* gene knockout, a band of 761 bp is expected, whereas for reversion to WT a band of 1,381 bp is expected. (**b**) Southern blot analysis. Hybridization with the probe gave two bands for the WT samples (around 2.7 kb and 7.8 kb), while the mutant samples gave just one band sized around 10 kb. Other bands are due to non-specific binding.

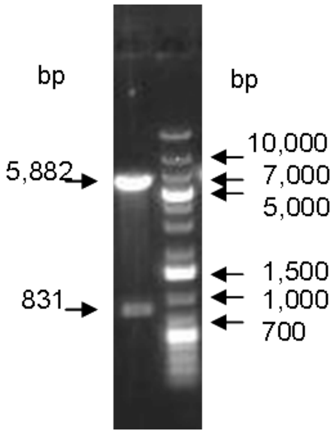

Figure S7. Restriction analysis of *NdeI/EcoRV* digested pIB-*salE* plasmid for complementation. The 5,882 bp band corresponds to the double-cut pIB139 plasmid, the 831 bp band to the cloned *salE* gene insert.

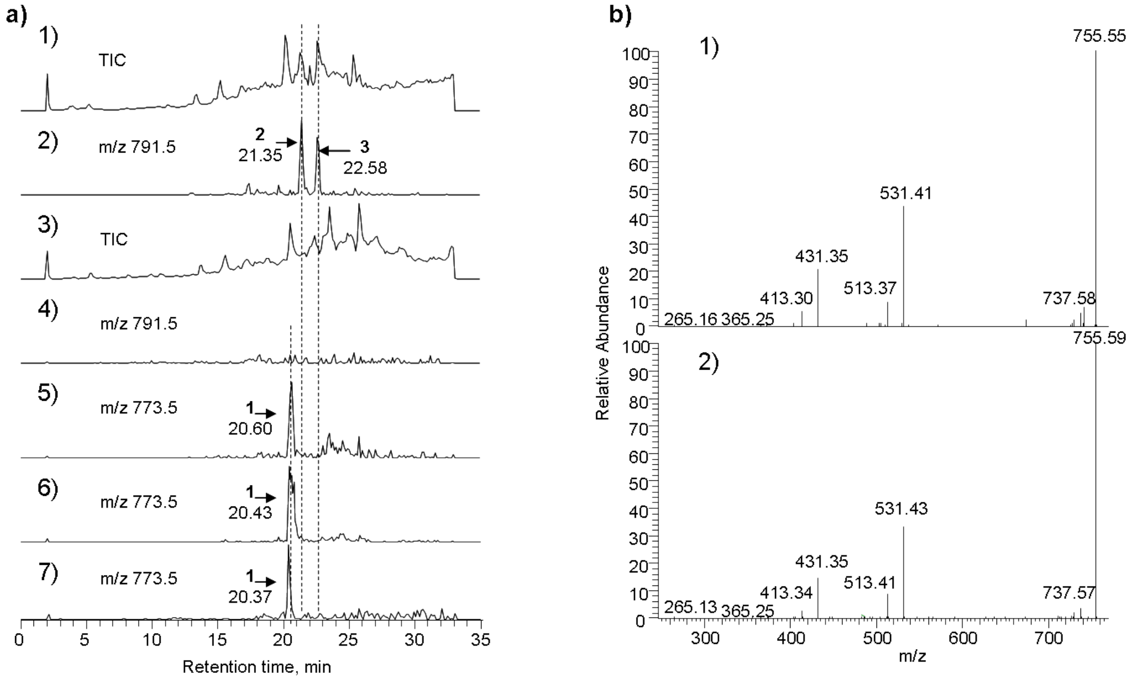

Figure S8. LC-ESI-MS analysis of metabolite profile of *S. albus* Δ*salE* mutant, *S. albus* Δ*salE*::pIB-*salE*, and *S. albus* WT strains. (**a**) 1 - LC-MS profile of ethyl acetate extract from *S. albus* Δ*salE* mutant, total ion current (TIC); 2 - *S. albus* Δ*salE* mutant, single ion current (SIC) m/z 791.5. The peak at 21.35 min corresponds to **2** $[M+Na]^+$, at 22.58 min to **3** $[M+Na]^+$; 3 - *S. albus* Δ*salE*::pIB-*salE* , TIC; 4 - *S. albus* Δ*salE*::pIB-*salE*, SIC m/z 791.5; 5 - *S. albus* Δ*salE*::pIB-*salE*, SIC m/z 773.5. The peak at 20.60 min corresponds to **1** $[M+Na]^+$; 6 - *S. albus* WT, SIC m/z 773.5. The peak at 20.43 min corresponds to **1** $[M+Na]^+$; 7 – salinomycin sodium salt standard (**1**), SIC m/z 773.5. The peak at 20.37 min corresponds to **1** $[M+Na]^+$. (**b**) ESI-MS/MS analysis of **1** produced by *S. albus* Δ*salE*::pIB-*salE* (spectrum 1) and *S. albus* WT (spectrum 2) strains. Identical MS/MS spectra suggest that the same compound **1** is produced in WT and complemented Δ*salE*::pIB-*salE* strains.

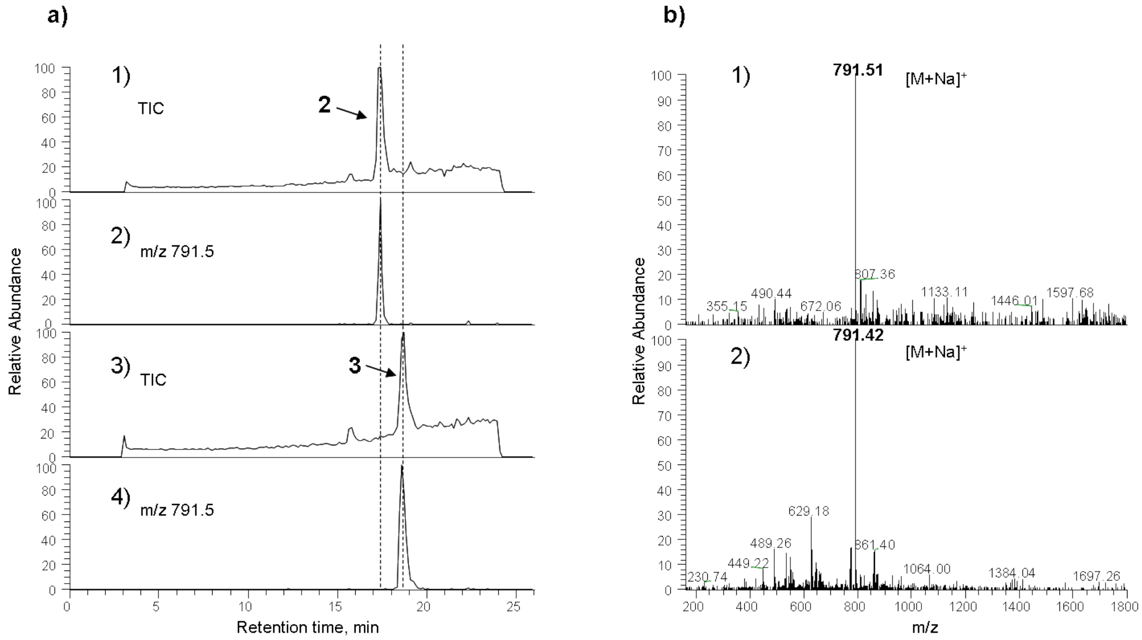

Figure S9. LC-ESI-MS analysis of purified compounds 2 and 3 from *S. albus* Δ*salE* mutant strain. (**a**) 1 - LC-MS profile of pure **2**, TIC; 2 - LC-MS profile of pure **2**, SIC m/z 791.5; 3 - LC-MS profile of pure **3**, TIC; 4 - LC-MS profile of pure **3**, SIC m/z 791.5. (**b**) ESI-MS spectra of purified componds **2** (spectrum 1) and **3** (spectrum 2). HPLC conditions used: Method B.

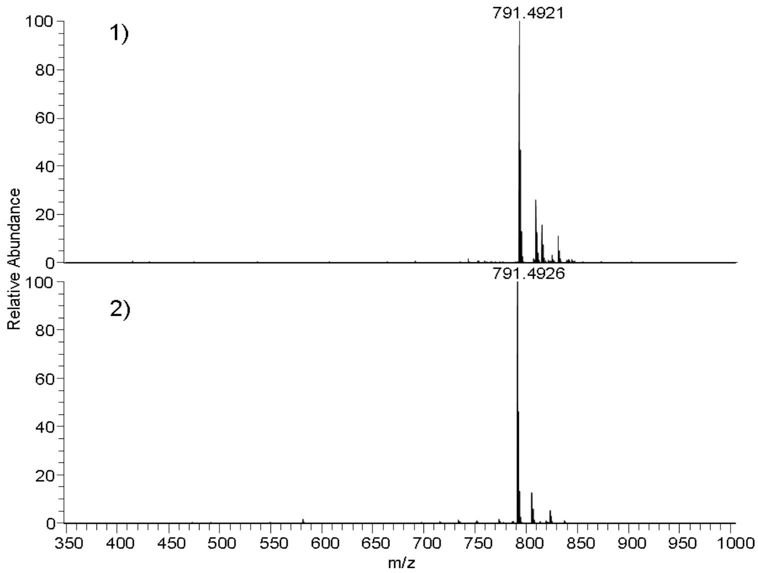

Figure S10. High-resolution MS analysis of $[M+Na]^+$ ions of compound 2 (spectrum 1) and 3 (spectrum 2), m/z $[M+Na]^+$ 791.4916.

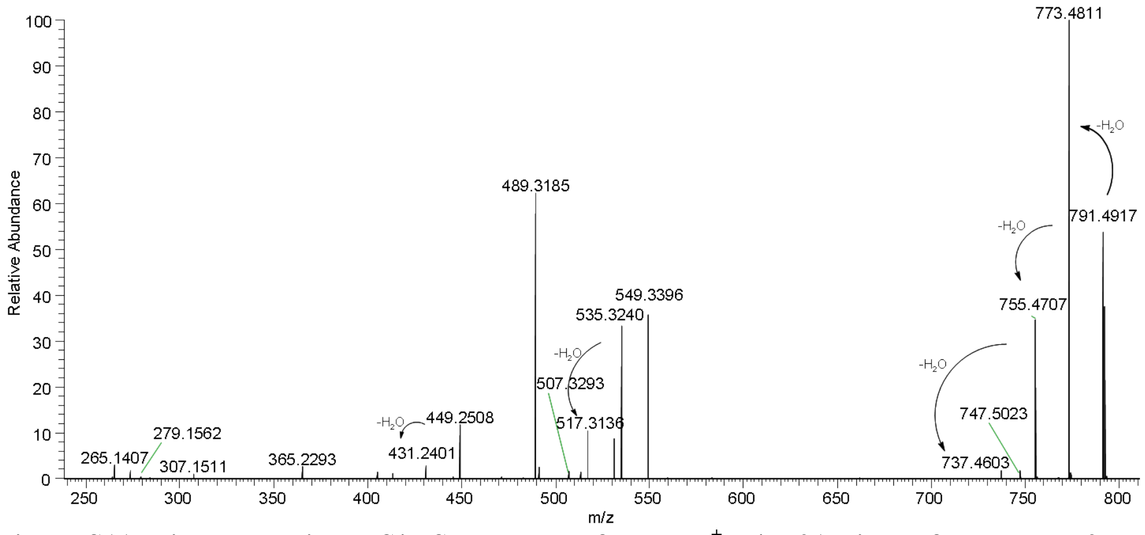

Figure S11. High-resolution MS/MS spectrum of $[M+Na]^+$ m/z 791.5 ions of compound 2.

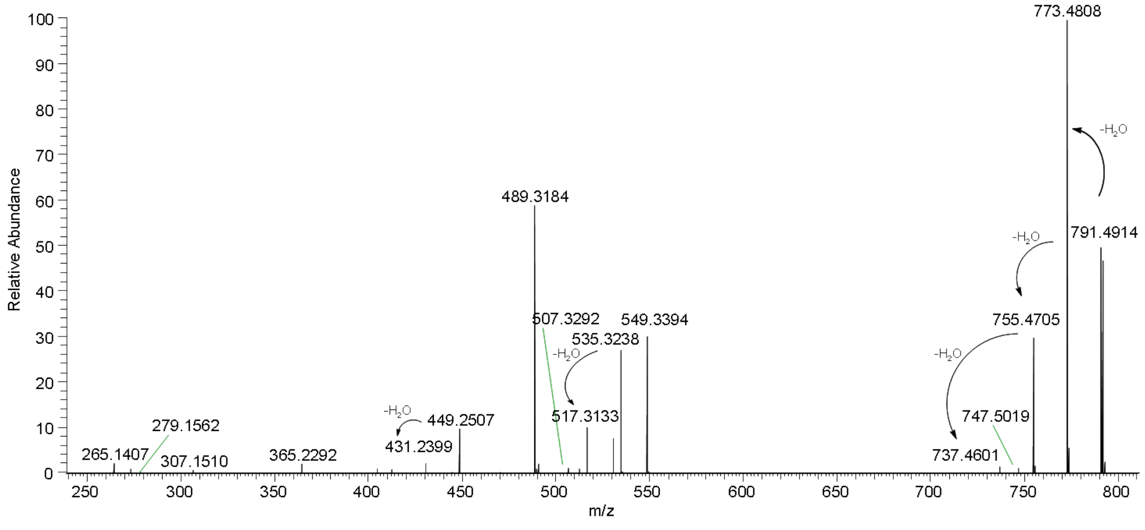

Figure S12. High-resolution MS/MS spectrum of $[M+Na]^+$ m/z 791.5 ions of compound 3.

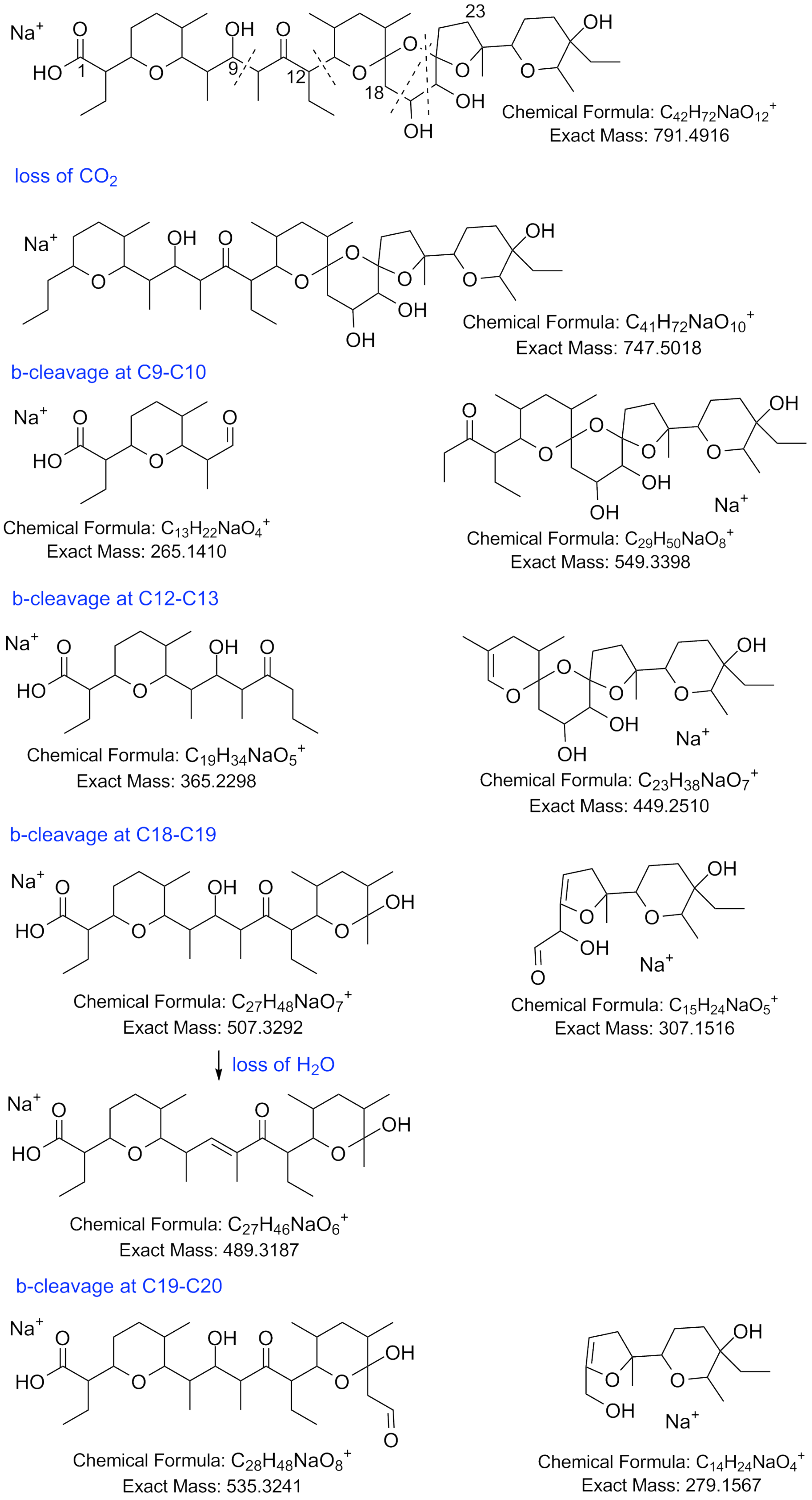

Figure S13. Proposed fragmentation pathways for compounds 2 and 3 based on MS/MS and MS^3 analysis. All the fragmentations occur due to β-cleavage, a McLafferty-like rearrangement. The formation of the ions at m/z 549.3 and m/z 449.3 suggests the presence on an extra –OH group in the "right hand side" of the molecule. The formation of the ions at m/z 507.3, 489.3 and 535.3 suggests the presence of C19-OH in the molecules **2** and **3**.

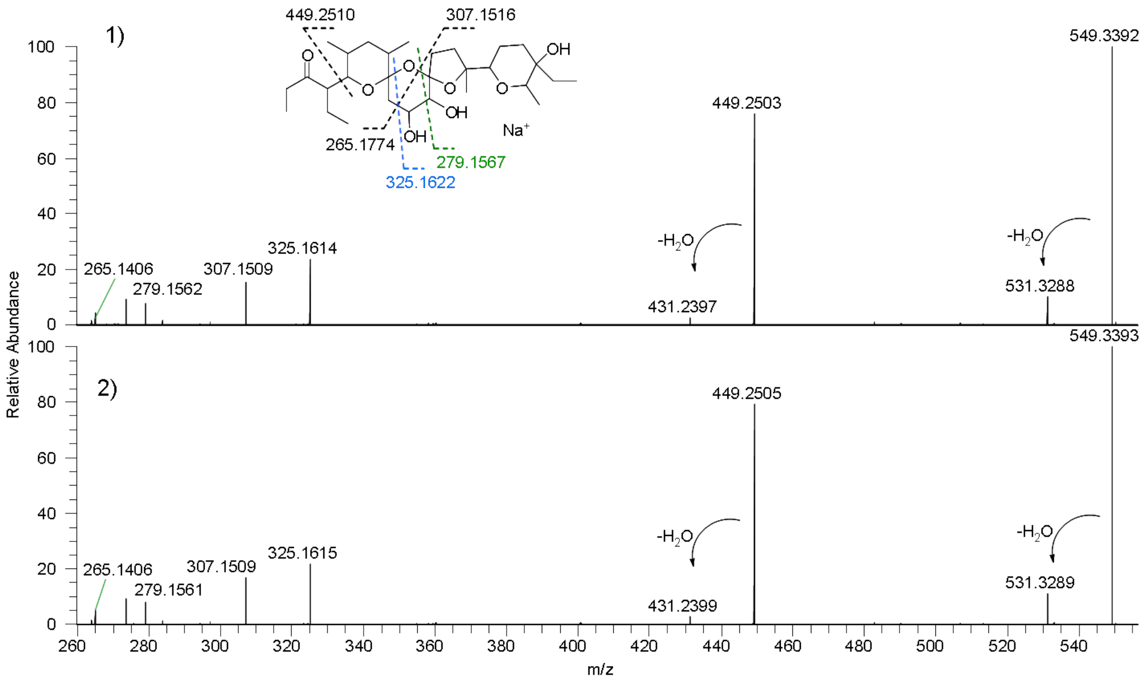

Figure S14. High-resolution MS^3 spectrum for m/z 549.3 [M+Na]$^+$ (791.5 → 549.3) for compounds 2 (spectrum 1) and 3 (spectrum 2).

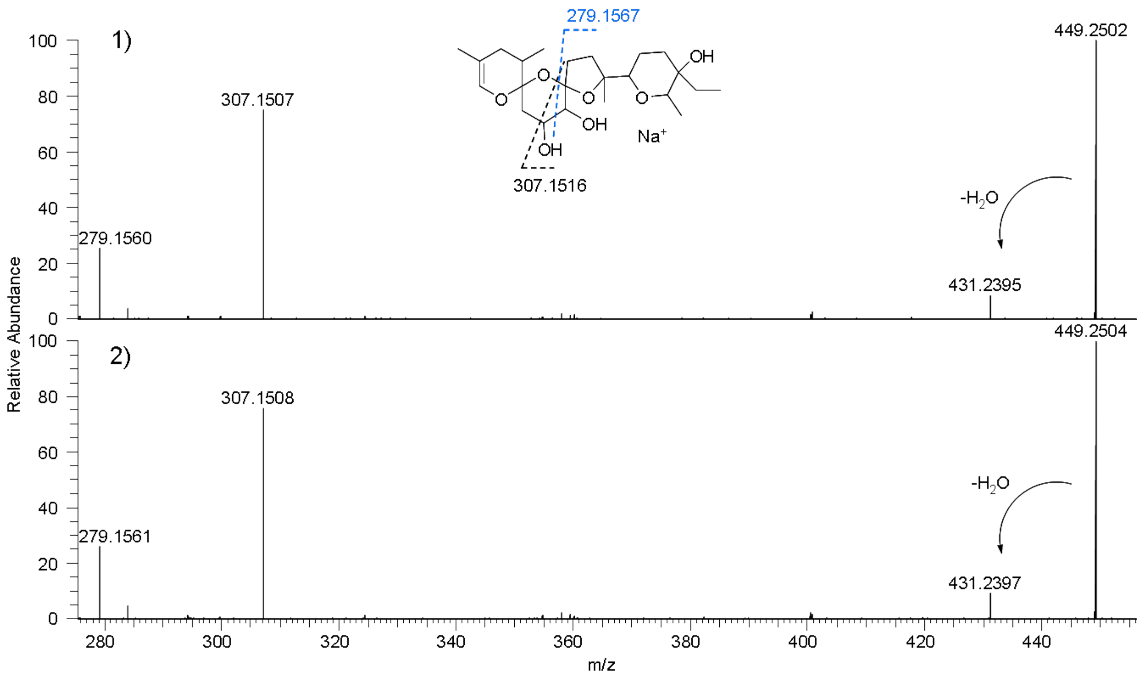

Figure S15. High-resolution MS^3 spectrum for m/z 449.3 [M+Na]$^+$ (791.5 → 449.3) for compounds 2 (spectrum 1) and 3 (spectrum 2).

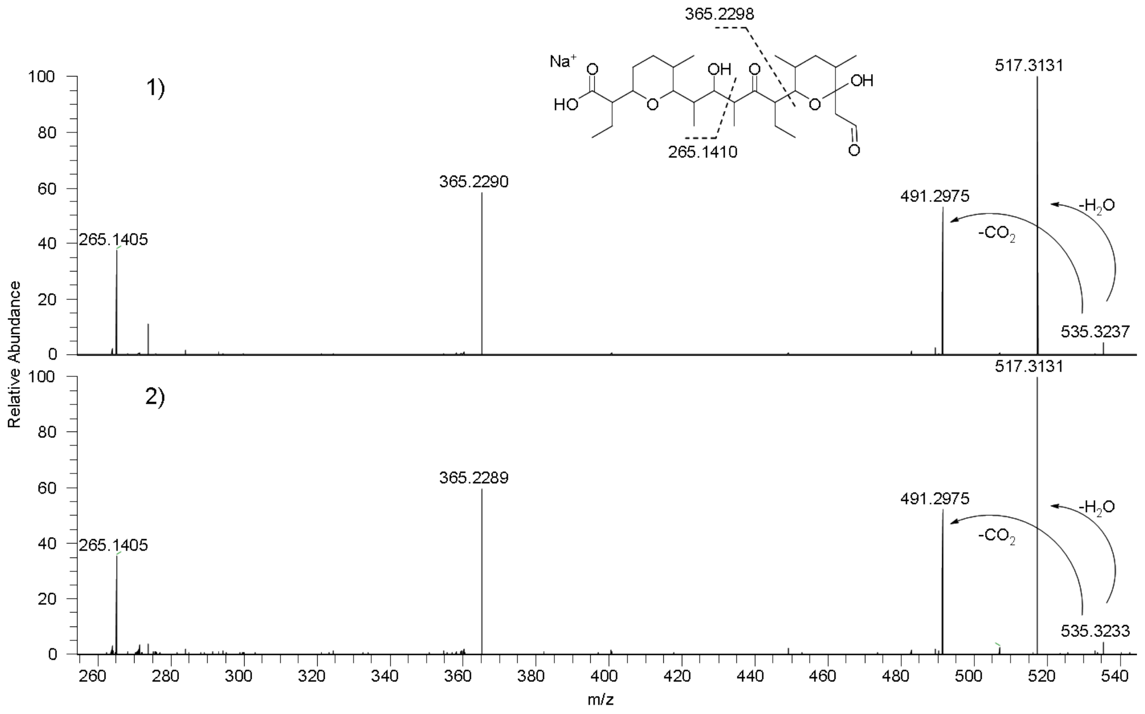

Figure S16. High-resolution MS^3 spectrum for m/z 535.3 $[M+Na]^+$ (791.5 → 535.3) for compounds 2 (spectrum 1) and 3 (spectrum 2).

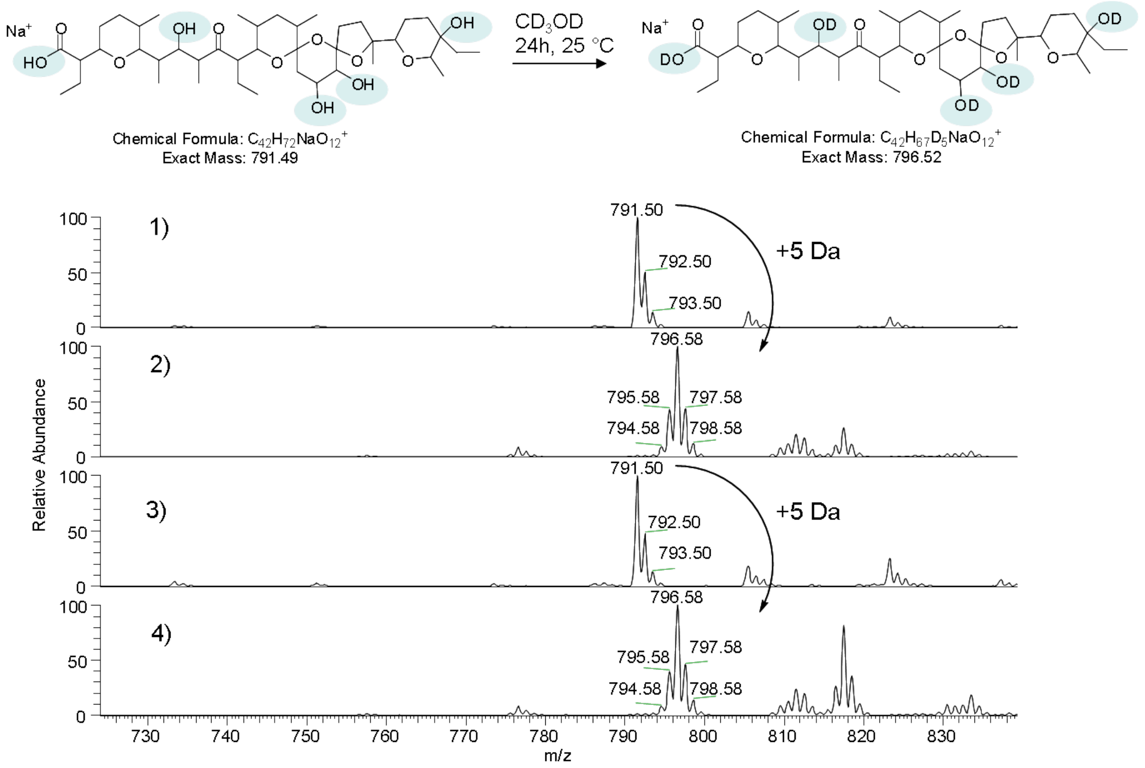

Figure S17. Comparison of MS spectrum of non-deuterated (spectra 1 and 3) and deuterated (spectra 2 and 4) $[M+Na]^+$ ions for compounds 2 (spectra 1 and 2) and 3 (spectra 3 and 4). 5 Da shift in the mass of molecular $[M+Na]^+$ ion at *m/z* 791.5 suggests the presence of five exchangeable protons in **2** and **3**.

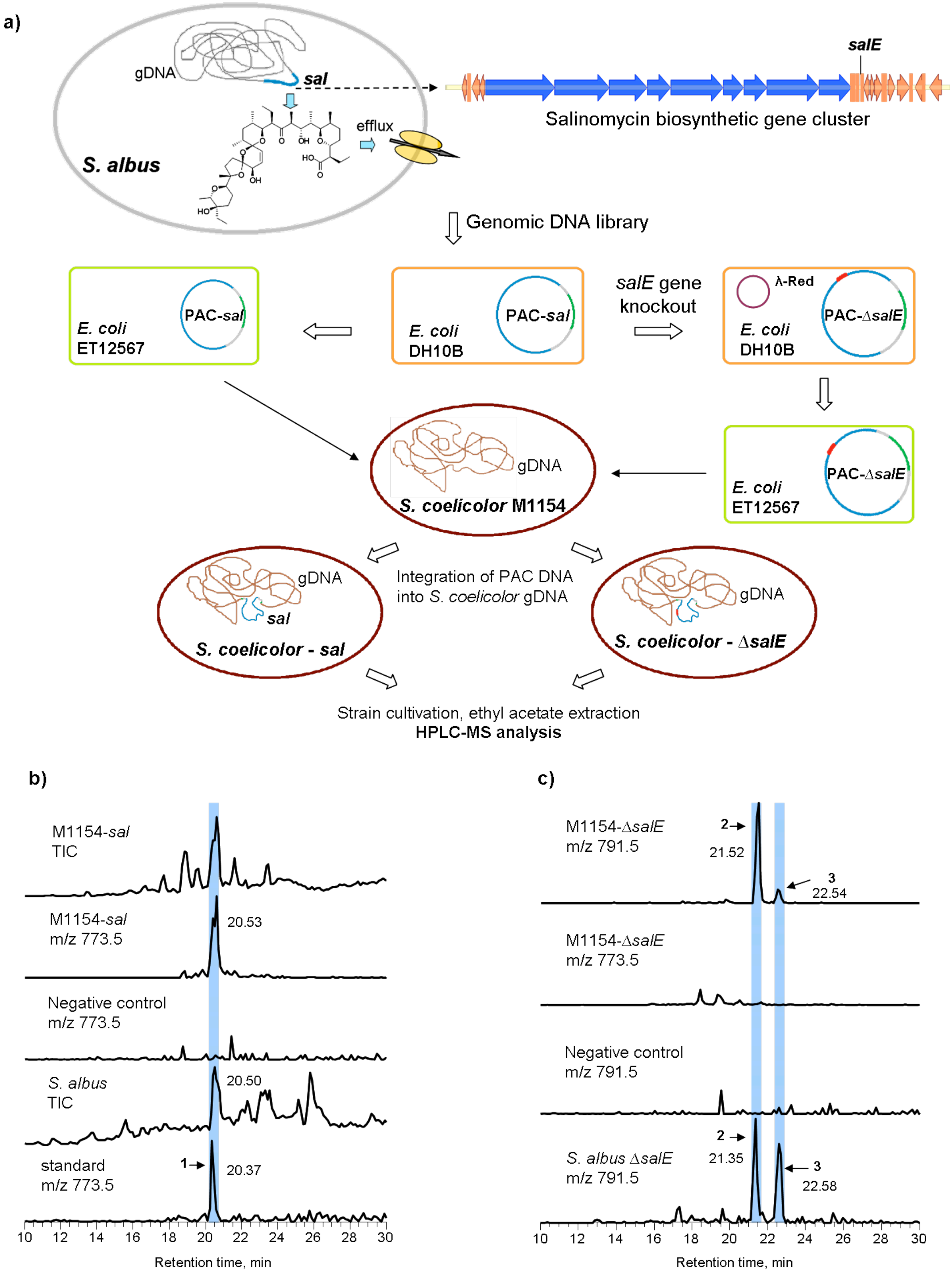

Figure S18. Heterologous expression of salinomycin biosynthetic gene cluster. (a) gDNA from salinomycin native producer *S. albus* was extracted to create a genomic library in PAC vector pESAC13; a clone containing the entire salinomycin biosynthetic gene cluster was used for conjugation with *S. coelicolor* M1154. In parallel this positive clone was used for deletion of the *salE* gene using λ-Red methodology. The resulting construct PAC-Δ*salE* was used for conjugation with *S. coelicolor* M1154. **(b)** HPLC-MS traces showing the production of **1** in *S. coelicolor* M1154, the peak at 20.53 min corresponding to salinomycin molecular ion at m/z $[M+Na]^+$ 773.5. **(c)** HPLC-MS traces showing the production of **2** and **3** in *S. coelicolor* M1154, the peaks at 21.52 min and 22.54 min corresponding to the molecular ions of **2** and **3** at m/z $[M+Na]^+$ 791.5. *S. coelicolor* M1154 with an integrated copy of pESAC13 was used as a negative control.

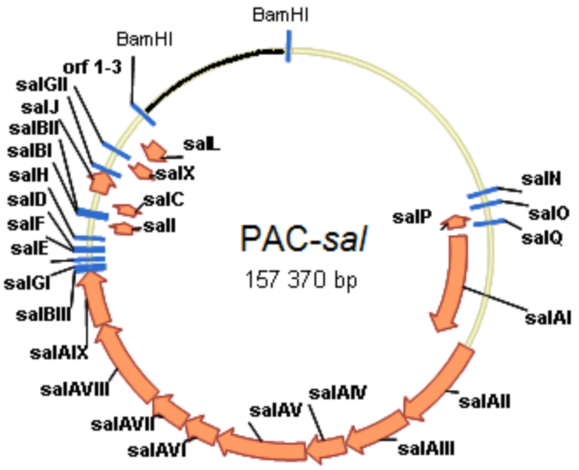

Figure S19. The map of the PAC vector containing the entire salinomycin gene cluster. The part of the vector originating from the pESAC13 vector is shown in black. The insert of 136,770 bp with the salinomycin biosynthetic gene cluster is cloned between two *BamHI* restriction sites. Genes involved in salinomycin biosynthesis are shown in blue and light red colors.

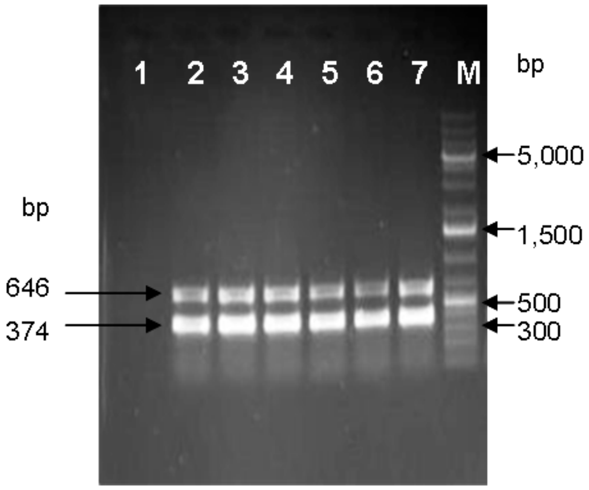

Figure S20. Confirmation of integration of the salinomycin gene cluster into the *S. coelicolor* M1154 genome. Lane 1 – negative control, gDNA from *S. coelicolor* M1154 into which the pESAC13 vector (without insert) was integrated had been used as a PCR template; Lanes 2, 3, 4 and 5 – PCR products for gDNA from *S. coelicolor* exconjugants potentially containing integrated PAC-*sal;* Lane 6 – positive control, PAC-*sal* from *E. coli* DH10B cells was used as a PCR template; Lane 7 - positive control, gDNA purified from *S. albus* WT was used as a PCR template. Expected band sizes: 374 bp for the beginning of the biosynthetic cluster primer pair, 646 bp for the end primer pair.

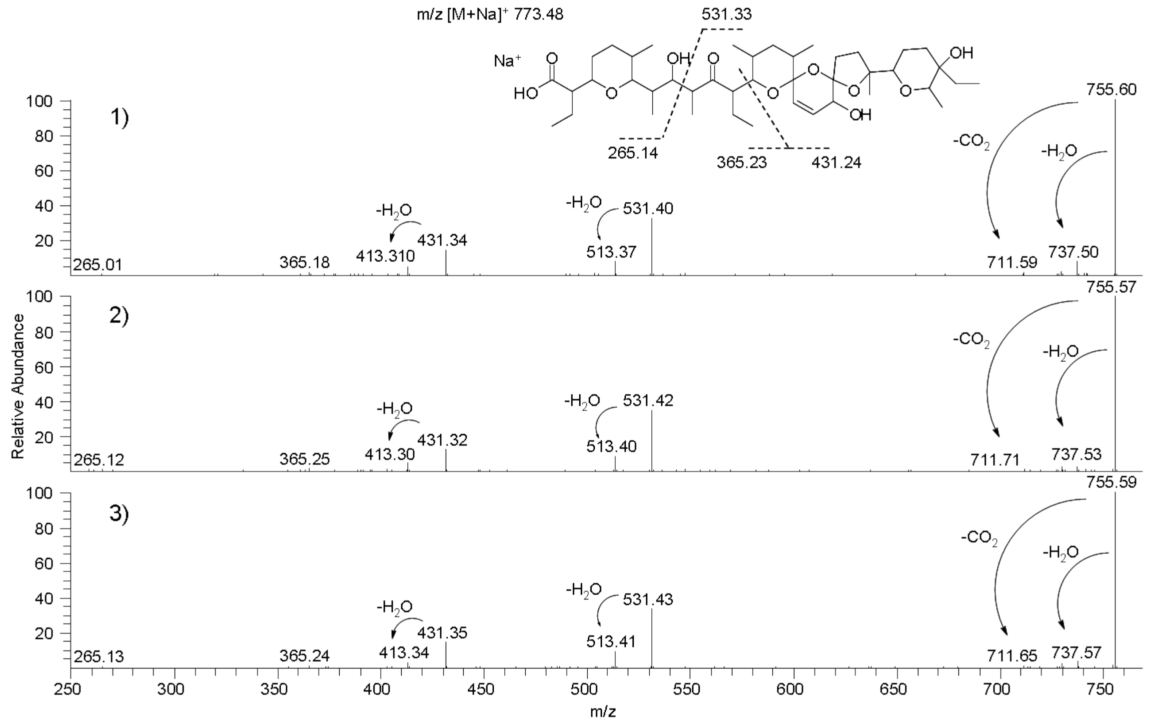

Figure S21. ESI-MS/MS analysis of m/z 773.5 [M+Na]$^+$ ions of compound 1 produced by *S. coelicolor* M1154 (spectrum 1), *S. albus* wild-type (spectrum 2), and salinomycin standard (spectrum 3). Identical MS/MS profiles suggest that the same compound in produced in the heterologous host and the wild-type strain.

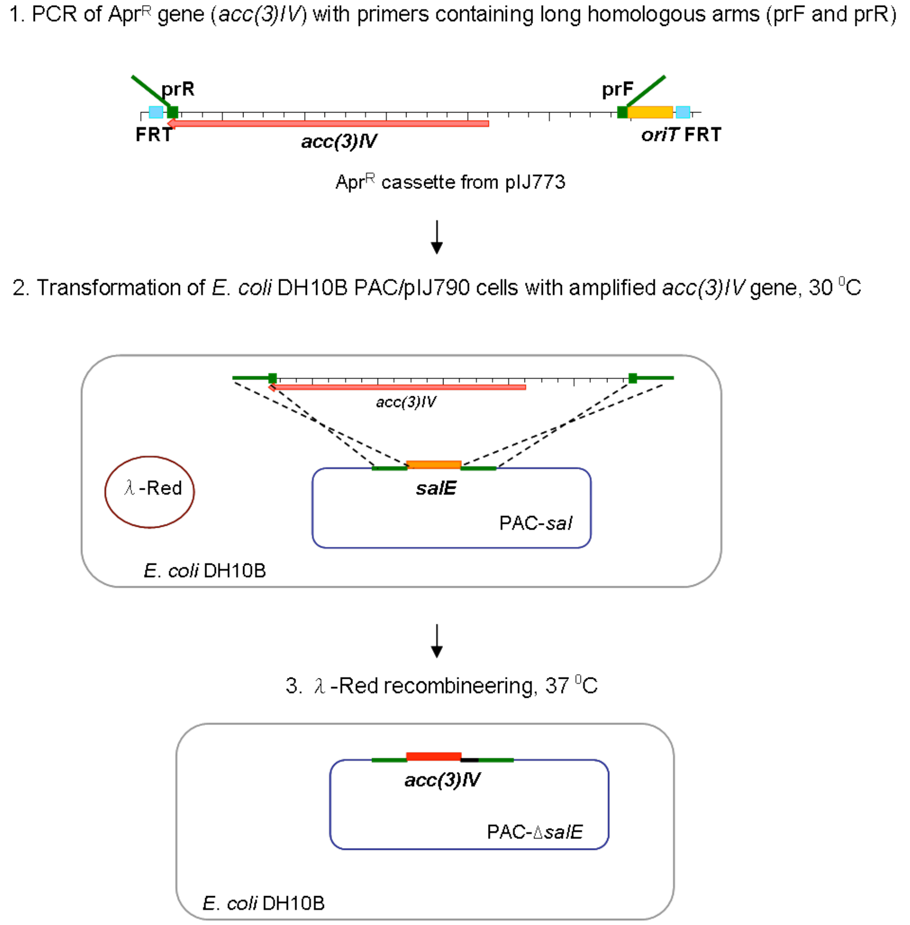

Figure S22. Schematic illustration of the use of λ-Red-based recombination to perform gene inactivation in the PAC clone.

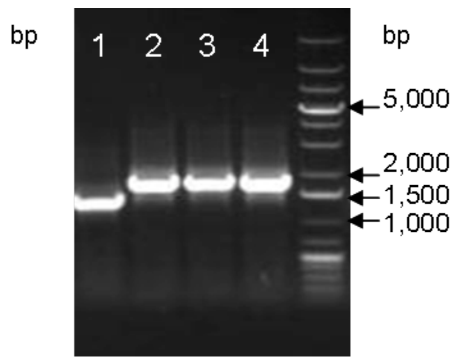

Figure S23. Confirmation of the replacement of the *salE* gene in PAC-*sal* by the *acc(3)IV* gene conferring apramycin resistance. Lane 1 - negative control PAC-*sal* DNA was used as a PCR template; Lanes 2, 3, 4 – PCR product from PAC DNA of purified from *E. coli* after replacement of *salE* by *acc(3)IV*. If *acc(3)IV* gene has replaced *salE* gene a DNA band is expected at 1,754 bp. If the *salE* gene remained intact a 1,379 bp band should appear.

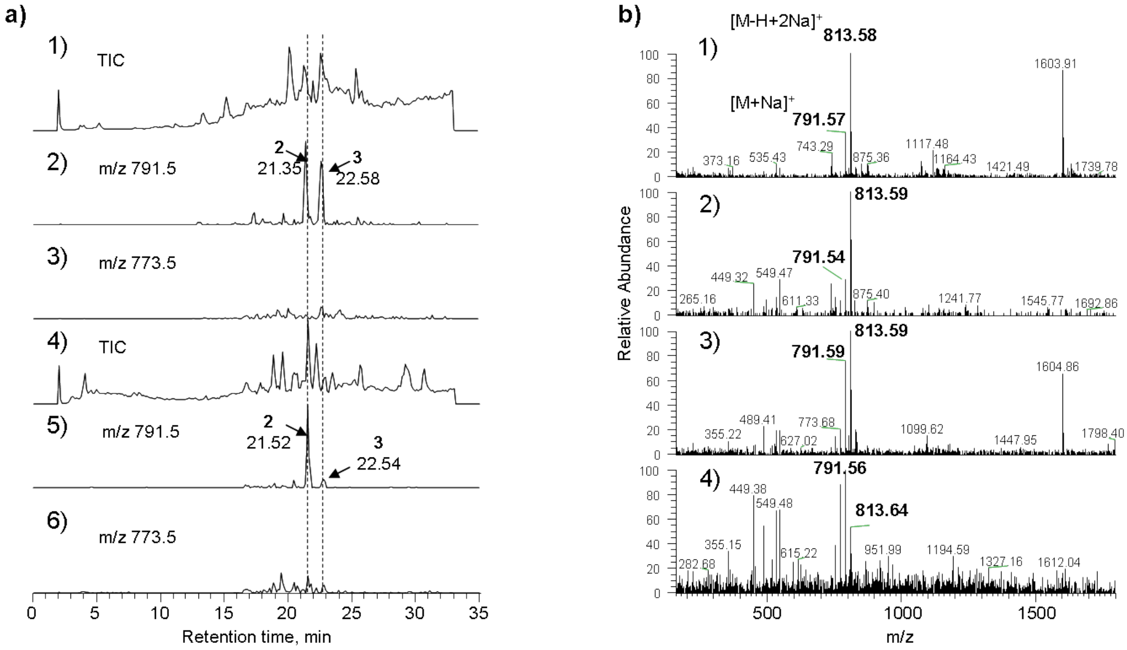

Figure S24. LC-ESI-MS analysis of metabolites in extracts of *S. albus* Δ*salE* mutant and of *S. coelicolor* M1154 after introduction of PAC-Δ*salE*. (a) 1 - LC-MS profile of ethyl acetate extract from *S. albus* Δ*salE* mutant, TIC; 2 - *S. albus* Δ*salE* mutant, SIC m/z 791.5. The peak at 21.35 min corresponds to **2** [M+Na]$^+$, at 22.58 min to **3** [M+Na]$^+$; 3 - *S. albus* Δ*salE* mutant, SIC m/z 773.5; 4 - LC-MS profile of ethyl acetate extract from *S. coelicolor* M1154 PAC-Δ*salE* mutant, TIC; 5 - *S. coelicolor* M1154 PAC-Δ*salE* mutant, SIC m/z 791.5. The peak at 21.35 min corresponds to **2** [M+Na]$^+$, at 22.54 min to **3** [M+Na]$^+$; 6 - *S. coelicolor* M1154 PAC-Δ*salE* mutant, SIC m/z 773.5. **(b)** ESI-MS analysis of **2** (spectra 1 and 3) and **3** (spectra 2 and 4) produced by *S. albus* Δ*salE* and *S. coelicolor* M1154 PAC-Δ*salE* mutant strains respectively.

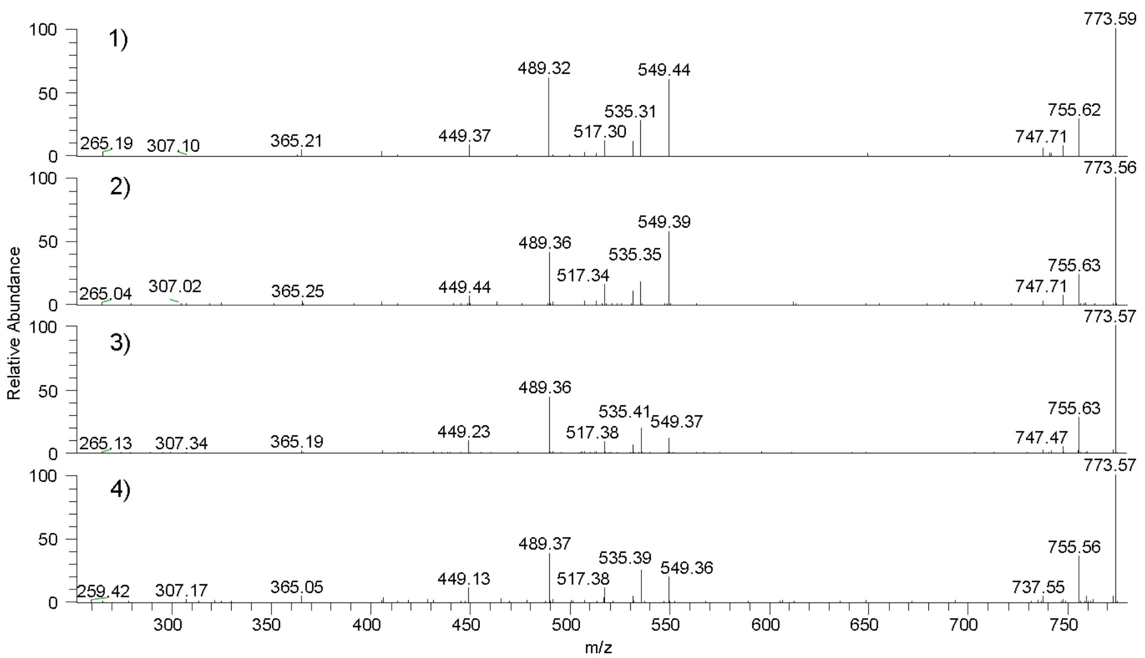

Figure S25. ESI-MS/MS analysis of m/z 791.5 $[M+Na]^+$ ions of compounds 2 (spectra 1 and 2) and 3 (spectra 3 and 4) produced by *S. albus* Δ*salE* mutant and *S. coelicolor* M1154 PAC-Δ*salE* mutant respectively. Identical MS/MS profiles suggest that the same compounds are produced by both strains.

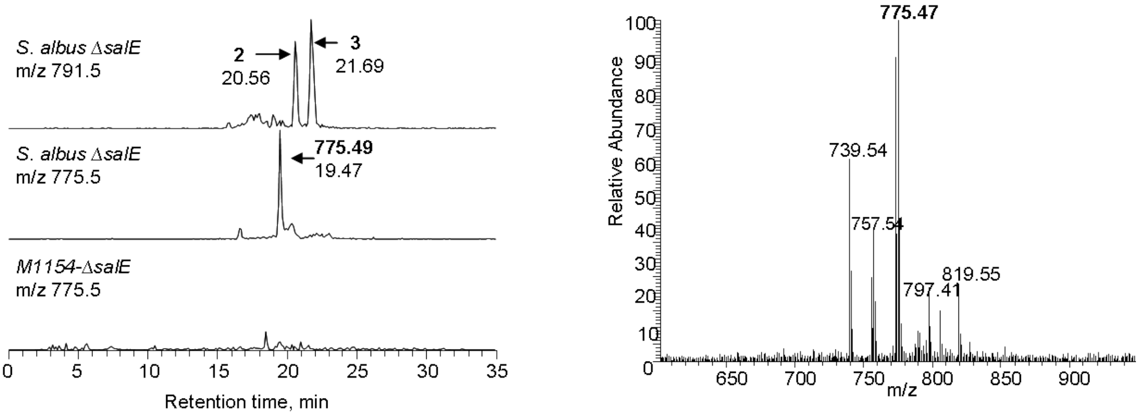

Figure S26. LC-ESI-MS analysis of the metabolite with m/z 775.5 detected in some culture extracts from the *S. albus* Δ*salE* strain.

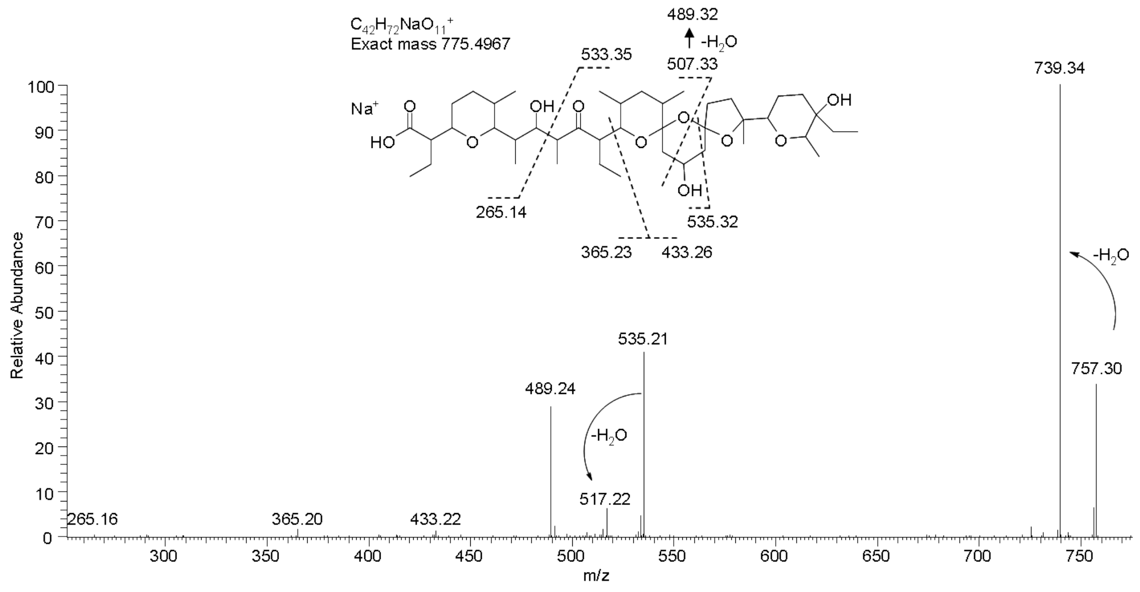

Figure S27. MS/MS spectrum of [M+Na]$^+$ m/z 775.5.

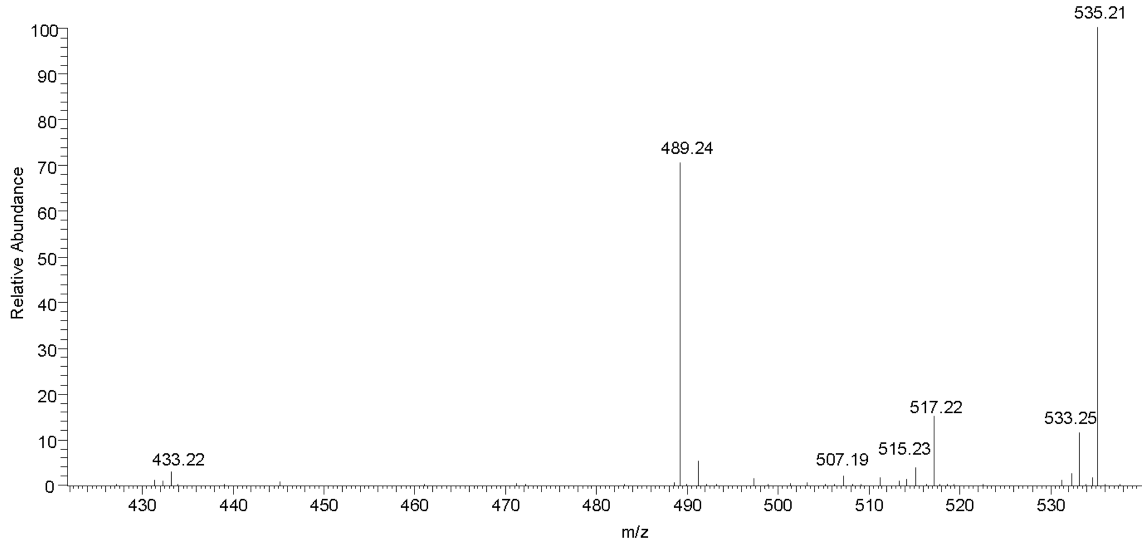

Figure S28. MS/MS spectrum of [M+Na]$^+$ m/z 775.5 (expansion of the region between m/z 420 and m/z 540 in Figure S27).

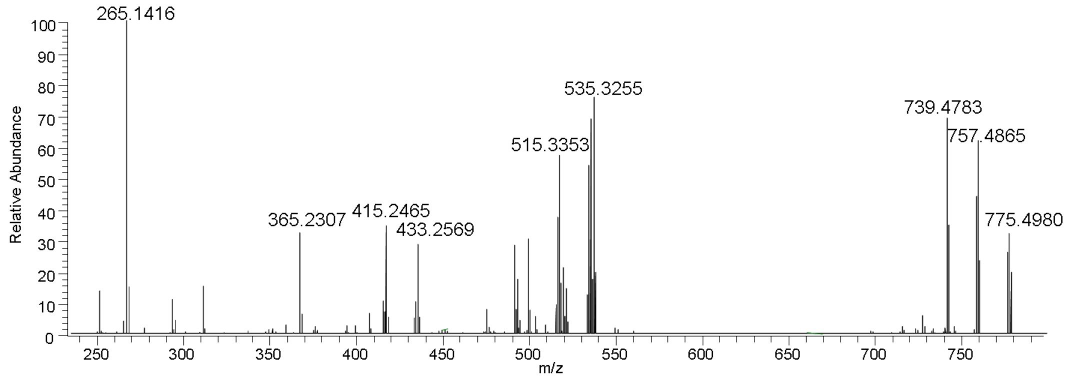

Figure S29. High-resolution MS/MS spectrum of [M+Na]$^+$ m/z 775.5 ion.

3. Supplementary Tables

Table S1. Bacterial strains used in this study.

Strain	Genotype/Characteristics	Reference
E. coli		
DH10B	F⁻ mcrA Δ(*mrr-hsd*RMS-*mcr*BC), Φ80*lac*ZΔM15, Δ l*ac*X74 *rec*A1 *end*A1 *ara*D139 Δ (*ara leu*)7697 *gal*U *gal*K *rpsL nup*G λ–<br>Host for general DNA manipulations	Invitrogen
ET12567 (pUZ8002)	(F⁻ *dam*-13::Tn9 *dcm*-6 *hsd*M *hsd*R recF143 zjj-202::Tn10 *gal*K2 *gal*T22 *ara*14 *pac*Y1 *xyl*-5 *leu*B6 *thi*-1)<br>Donor strain for conjugation between *E. coli* and *Streptomyces*	[7]
ET12567	(F⁻ *dam*-13::Tn9 *dcm*-6 *hsd*M *hsd*R recF143 zjj-202::Tn10 *gal*K2 *gal*T22 *ara*14 *pac*Y1 *xyl*-5 *leu*B6 *thi*-1)<br>Donor strain for conjugation between *E. coli* and *Streptomyces* in triparental mating	[7]
TOPO10 (pR9604)	Helper strain in triparental mating	[4]
S. albus		
DSM 41398	wild type strain producing salinomycin	DSMZ
DSM 41398 Δ*salE*	*salE* gene in-frame deletion mutant	this work
DSM Δ*salE*-pIB-*salE*	*salE* gene in-frame deletion mutant complemented with *salE* gene	this work
S. coelicolor		
M1154	Heterologous host for salinomycin production	[8]
M1154-pESAC13	Negative control for heterologous expression	this work
M1154-PAC-*sal*	Heterologous host with integrated into the genome the entire salinomycin biosynthetic gene cluster	this work
M1154-PAC-Δ*salE*	Heterologous host with integrated into the genome the entire salinomycin biosynthetic gene cluster with inactivated *salE* gene	this work

Table S2. Plasmids used in this work.

Plasmid	Genotype/Characteristics	Reference
pYH7	*E.coli-Streptomyces* shuttle vector	[9]
pHLΔ*salE*	*salE* gene disruption construct in which a 621 bp internal fragment of *salE* gene was deleted in-frame	this work
pIB139	*ϕC31* site integrative vector	[10]
pIB-*salE*	SalE complementation construct in which 831 bp *salE* gene is cloned into pIB139 (*NdeI*/*EcoRV*)	this work
pESAC13	*bla, neo, tsr, parA, parB, sacB,oriT* PAC library	[4]
PAC-*sal*	pESAC13 based PAC vector with cloned salinomycin biosynthetic gene cluster	this work
PAC-Δ*salE*	pESAC13 based PAC vector with cloned salinomycin biosynthetic gene cluster with inactivated *salE* gene	this work
pIJ773	FRT-*oriT*-*acc(3)IV*-FRT cassette, used for the amplification of *acc(3)IV* gene	[11]
pIJ790	λ-RED (*gam, bet, exo*), *cam, araC,* rep101^{ts}, oriR101, P araBAD	[11]

Table S3. Oligonucleotide primers used in this work.

Name	Sequence (written in 5' – 3' direction)
Primers used for PAC library screening	
salPAC beg_F	CGAGAGGAAGGTACGGACCGG
salPAC beg_R	CTGGTCTTCGACCTCACCGGCAA
salPAC cen_F	GAAGCTCTCCGACGCCGAGGCCA
salPAC cen_R	ACGTGCTGTTCGAGGATGACGTG
salPAC end_F	GAAGAGCCGCTTGCGCAGCTGGGA
salPAC end_R	CGAGCGTCCCGTAGTCGAGGCGTT
Primers used for salE gene in frame deletion	
SalE_1F	TGATCAAGGCGAATACTTCATATGGGAGCAGTCCGTACAACGGGCCCT
SalE_1R	TCATGATCTTGCTCGACTCGCTGACCGTGACCGCCA
SalE_2F	TCAGCGAGTCGAGCAAGATCATGAACGCGATACCGG
SalE_2R	CCGCGCGGTCGATCCCCGCATATGTGCACTAGTCACGAATCTTCGAGG
Primers used for salE gene inactivation in PAC vector	
salE_to_Apr cas_PAC_F	GTACCGACCTCACGAAACGGAAAACGGTAATCCATGGAGACGAAGCC GGCGCTGACGCCGTTGGATACACCAAG
salE_to_Apr cas_PAC_R	GGCTCAGAAGGAGTAGCGCATCACGCGGTTGTAGTGCCGCAGCGCCGG TATTTATGAGCTCAGCCAATCGACTGG
Primers used for PCR confirmation of salE gene deletion	
PCR salE_f	GCAGGCCTGGCAGGAGCTCAG
PCR salE_r	CACCGGCATGTGCGTCCACGC
Primers used for cloning of salE gene into pIB139 vector	
salE_NdeI_F	GAC**CATATG**GAGACGAAGCCGGCGCA
salE_EcoRV_R	GCG**GATATC**TCAGAAGGAGTAGCGCATCAC

* The underlined letters represent 51 nucleotide extensions with sequence homology.

Table S4. Deduced function of genes of salinomycin cluster.[6]

Name of orf	Length (aa)	Proposed Function	Similarity with	Identity/ Similarity (%)
salN	327	putative regulator	putative regulatory protein, *S. kanamyceticus*, ADU56285	44/60
salO	160	putative regulator	conserved hypothetical protein, *S.* sp. *C*, ZP_07291838	67/80
salP	572	3-hydroxybutyryl-CoA dehydrogenase	predicted protein, *S.* sp. C, ZP_07291839	69/78
salQ	342	putative 3-oxoacyl-(acyl carrier protein) synthase	3-oxoacyl-[acyl-carrier-protein] synthase III, *S.* sp. *C*, ZP_07291840	77/86
salAI	4925	polyketide synthase (KSQ, AT, ACP, KS, AT, DH, KR ACP, KS, AT, DH, ER, KR, ACP)	modular polyketide synthase, *S.* sp. *C*, ZP_07291842	71/79
salAII	3917	polyketide synthase (KS, AT, DH, KR, ACP, KS, AT, DH, ER, KR, ACP)	modular polyketide synthase, *S.* sp. *C*, ZP_07291842	64/74
salAIII	2692	polyketide synthase (KS, AT, ACP, KS, AT, KR, ACP)	modular polyketide synthase, *S.* sp. *C*, ZP_07291843	73/81

salAIV	1642	polyketide synthase (KS, AT, KR, ACP)	modular polyketide synthase, *S.* sp. *C*, ZP_07291844	72/80
salAV	3750	polyketide synthase (KS, AT, DH, ER, KR, ACP, KS, AT, KR, ACP)	modular polyketide synthase, *S.* sp. *C*, ZP_07291845	76/84
salAVI	1435	polyketide synthase (KS, AT, DH, ACP)	modular polyketide synthase, *S.* sp. *C*, ZP_07291845	65/76
salAVII	1644	polyketide synthase (KS, AT, KR, ACP)	modular polyketide synthase, *S. bingchenggensis* BCW-1, ADI04505	59/70
salAVIII	3724	polyketide synthase (KS, AT, KR, ACP, KS, AT, DH, ER, KR, ACP)	modular polyketide synthase, *S.* sp. *C*, ZP_07291849	67/77
salAIX	2312	polyketide synthase (KS, AT, KR, ACP, KSX)	modular polyketide synthase, *S.* sp. *C*, ZP_07291850	68/76
salBIII	128	epoxide hydrolase/cyclase	predicted protein, *S.* sp. *C*, ZP_07291851	75/85
salGI	265	putative type II thioesterase	thioesterase , *S.* sp. *C*, ZP_07291852	77/83
salE	271	putative O-methyltransferase	O-methyltransferase-like protein, *Saccharopolyspora erythraea* NRRL 2338, YP_001108765	39/61
salF	93	ferredoxin	ferredoxin, *S.* sp. ATCC 55098, ADU56364	37/47
salD	393	cytochrome P450	cytochrome P450, *Micromonospora* sp. ATCC 39149, ZP_04607068	52/68
salH	319	putative salinomycin export	ABC transporter ATP-binding protein, *S.* sp. *C*, ZP_07291854	76/84
salI	546	putative salinomycin export	antibiotic ABC transporter efflux pump, *Thermomonospora curvata* DSM 43183, YP_003302161	51/66
salBI	149	epoxide hydrolase/cyclase	predicted protein, *S.* sp. *C*, ZP_07291856	91/97
salBII	153	epoxide hydrolase/cyclase	NigBI, *S.* sp. *C*, ZP_07291858	82/90
salC	484	epoxidase	monensin epoxidase, *S.* sp. *C*, ZP_07291857	74/82
salJ	906	regulatory	predicted protein, *S.* sp. *C*, ZP_07291859	62/73
salGII	253	putative type II thioesterase	predicted protein, *S.* sp. *C*, ZP_07291861	72/82
salX	597	non-ribosomal peptide synthase C, PCP	peptide synthetase, *S. anulatus*, ADG27359	48/61
orf1	74	unknown	hypothetical protein, *S. bingchenggensis* BCW-1, ADI04514	54/63
orf2	73	unknown	hypothetical protein, *Mycobacterium colombiense* CECT 3035, ZP_08716349	69/75
orf3	73	unknown	conserved hypothetical protein, *S.* sp. *C*, ZP_07284641	72/78
salL	849	regulatory	predicted protein, *S. sp. C*, ZP_07291859	62/73
orf4	606	acyl-CoA synthetase	palmitoyl-CoA synthetase, *S. sp.* ACZ65474.1	46/58
orf5	572	acyl-CoA dehydrogenase	acyl-CoA dehydrogenase, *S. sp.* ZP_05511694.1	49/59
orf6	96	peptide carrier protein	peptide carrier protein, *S. sp.* ZP_05511693.1	62/72
orf7	279	4'-phosphopantetheinyl transferase	putative 4'-phosphopantetheinyl transferase, *S. sp.* ZP_05511687.1	53/63

Table S5. MS data of 2 and 3 measured by HR-ESI-Orbitrap MS.

Compound	Formula	calc. m/z $[M+Na]^+$	det. m/z $[M+Na]^+$	Error [ppm]
**2**	$C_{42}H_{72}O_{12}Na^+$	791.4916	791.4921	0.63
**3**	$C_{42}H_{72}O_{12}Na^+$	791.4916	773.4926	1.26

Table S6. MS/MS data of compound 2 at m/z $[M+Na]^+$ 791.5 measured by HR-ESI-Orbitrap MS.

Formula	calc. m/z $[M+Na]^+$	det. m/z $[M+Na]^+$	Error [ppm]
$C_{42}H_{72}O_{12}Na^+$	791.4916	791.4917	0.13
$C_{42}H_{70}O_{11}Na^+$	773.4810	773.4811	0.13
$C_{42}H_{68}O_{10}Na^+$	755.4705	755.4707	0.26
$C_{41}H_{72}O_{10}Na^+$	747.5018	747.5023	0.67
$C_{42}H_{66}O_{9}Na^+$	737.4599	737.4603	0.54
$C_{29}H_{50}O_{8}Na^+$	549.3398	549.3396	-0.36
$C_{28}H_{48}O_{8}Na^+$	535.3241	535.3240	-0.19
$C_{28}H_{46}O_{7}Na^+$	517.3136	517.3136	0.00
$C_{27}H_{48}O_{7}Na^+$	507.3292	507.3293	0.20
$C_{27}H_{46}O_{6}Na^+$	489.3187	489.3185	-0.41
$C_{23}H_{38}O_{7}Na^+$	449.2510	449.2508	-0.45
$C_{23}H_{36}O_{6}Na^+$	431.2404	431.2401	-0.70
$C_{19}H_{34}O_{5}Na^+$	365.2298	365.2293	-1.37
$C_{15}H_{24}O_{5}Na^+$	307.1516	307.1511	-1.63
$C_{14}H_{24}O_{4}Na^+$	279.1567	279.1562	-1.79
$C_{13}H_{22}O_{4}Na^+$	265.1410	265.1407	-1.13

Table S7. MS/MS data of compound 3 at m/z $[M+Na]^+$ 791.5 measured by HR-ESI-Orbitrap MS.

Formula	calc. m/z $[M+Na]^+$	det. m/z $[M+Na]^+$	Error [ppm]
$C_{42}H_{72}O_{12}Na^+$	791.4916	791.4914	-0.25
$C_{42}H_{70}O_{11}Na^+$	773.4810	773.4808	-0.26
$C_{42}H_{68}O_{10}Na^+$	755.4705	755.4705	0.00
$C_{41}H_{72}O_{10}Na^+$	747.5018	747.5019	0.13
$C_{42}H_{66}O_{9}Na^+$	737.4599	737.4601	0.27
$C_{29}H_{50}O_{8}Na^+$	549.3398	549.3394	-0.73
$C_{28}H_{48}O_{8}Na^+$	535.3241	535.3238	-0.56
$C_{28}H_{46}O_{7}Na^+$	517.3136	517.3133	-0.58
$C_{27}H_{48}O_{7}Na^+$	507.3292	507.3292	0.00
$C_{27}H_{46}O_{6}Na^+$	489.3187	489.3184	-0.61
$C_{23}H_{38}O_{7}Na^+$	449.2510	449.2507	-0.67
$C_{23}H_{36}O_{6}Na^+$	431.2404	431.2399	-1.16
$C_{19}H_{34}O_{5}Na^+$	365.2298	365.2292	-1.64
$C_{15}H_{24}O_{5}Na^+$	307.1516	307.1510	-1.95
$C_{14}H_{24}O_{4}Na^+$	279.1567	279.1562	-1.79
$C_{13}H_{22}O_{4}Na^+$	265.1410	265.1407	-1.13

Table S8. MS/MS data of m/z 775.5 metabolite measured by HR-ESI-Orbitrap MS.

Formula	calc. m/z $[M+Na]^+$	det. m/z $[M+Na]^+$	Error [ppm]
$C_{42}H_{72}O_{11}Na^+$	775.4967	775.4980	1.68
$C_{42}H_{70}O_{10}Na^+$	757.4861	757.4865	0.53
$C_{42}H_{68}O_9Na^+$	739.4756	739.4783	3.65
$C_{29}H_{50}O_7Na^+$	535.3241	535.3255	2.62
$C_{29}H_{48}O_6Na^+$	515.3343	515.3353	1.94
$C_{23}H_{38}O_6Na^+$	433.2561	433.2569	1.85
$C_{23}H_{36}O_5Na^+$	415.2455	415.2465	2.41
$C_{19}H_{34}O_5Na^+$	365.2298	365.2307	2.46
$C_{13}H_{22}O_4Na^+$	265.1410	265.1416	2.26

NMR Analysis

Careful analysis of the edited HSQC, TOCSY, HSQC-TOCSY and COSY spectra identified 10 individual spin systems for both compounds **2** and **3** and enabled the assignment of the proton and carbon NMR spectra. COSY and HMBC correlations then enabled the 2D structure to be determined as shown in Figure S30 where the C18/C19 double bond of salinomycin had been hydrated as proposed.

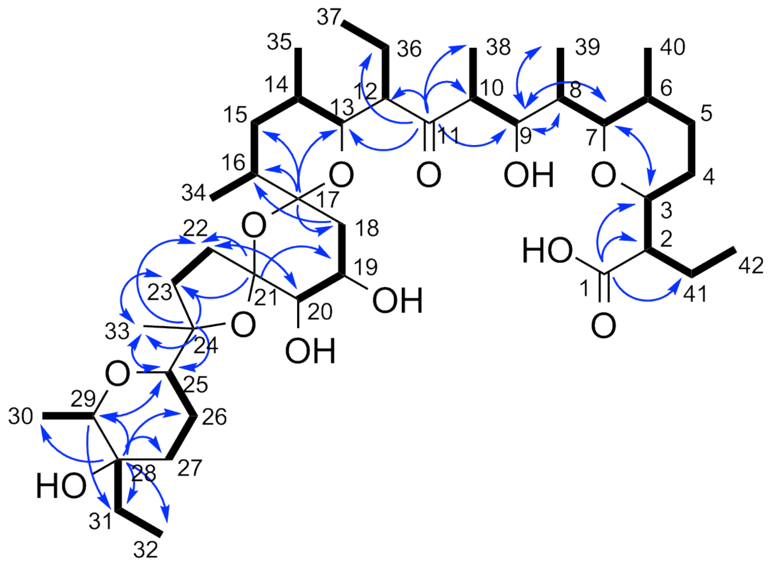

Figure S30. Proposed 2D structure for 2 and 3 showing spin systems in bold and key HMBC correlations used to connect them. Arrows represent a correlation from the carbon at the origin of the arrow to a proton at the head.

Configuration of the *bis*-spiroacetal region in compounds 2 and 3

NOESY and ^1H-^1H coupling constant data was used to determine the stereochemistry of the newly formed hydroxyl centre at C19 and the configuration of the *bis*-spiroacetal region.

For compound **3** H15b appeared as an apparent quartet with a $^3J_{H\text{-}H}$ of 13Hz. This implied a diaxial relationship to both H14 and H16 and suggested that the B ring should be in a chair conformation with the substituents on C13, C14 and C16 equatorial. An NOE between H13 and H15b supported this conformation (Figure S31).

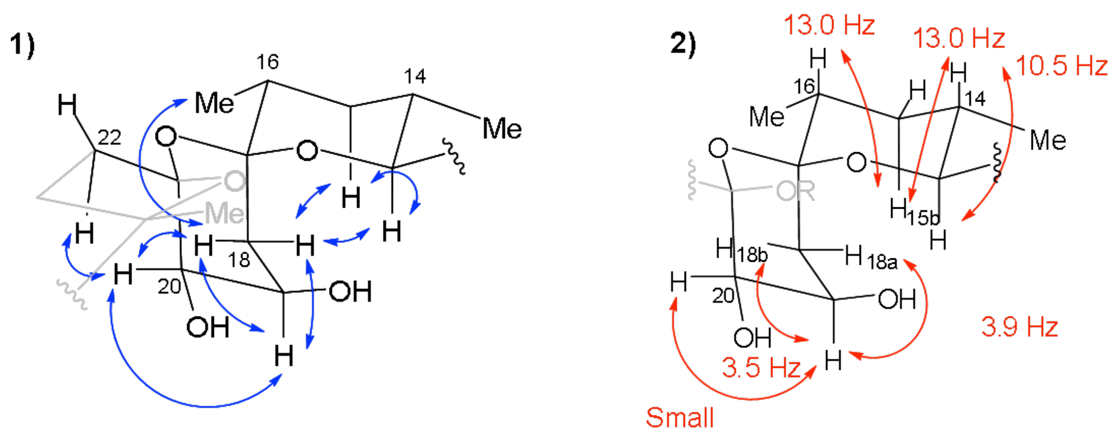

Figure S31. NMR analysis of *bis*-spiroacetal ring system in 3. 1 – Conformation of the BCD ring system showing key NOE correlations. 2 – Key coupling constants in the B and C rings.

NOE correlations between H13/H18a and H15b/H18a placed C18 on the bottom face of the B ring in the axial position with H18a pointing into the middle of the B ring. Analysis of the coupling constants in the C18-C20 region revealed small couplings for H18a/H19 (3.9 Hz) and H18b/H19 (3.5 Hz) consistent with a gauche relationship between these pairs of protons and suggesting that H19 should be equatorial. Both H19 and H20 appeared broadened by their attached hydroxyl groups but neither appeared to show a large coupling, again consistent with an equatorial orientation for H19, NOE correlations from H19 to both H18a and H18b confirmed this. The configuration of C20 was determined by an NOE correlation between H20 and H18b which placed both these protons in an axial position.

A further NOE correlation between H20/H22a suggested that C22 should be equatorial in relation to the C ring and thus confirmed the configuration of C21.

For compound **2** a similar analysis of the B ring led to conclusion that it was again in a chair conformation with an axial orientation of the C13, C14 and C16 substituents. In this case however NOE's were observed between H16/H18b and H34/H18a suggesting that C18 was in an equatorial position relative to the B ring and that C17 had the opposite configuration to **3** and **1** (salinomycin sodium salt, purchased from Sigma Aldrich). It was also observed that the ^{13}C chemical shift for C18 changes from 26.00 in **3** to 40.10 in **2**.

Analysis of the C ring was initially hindered by coalescence of the H18a and H18b signals, this problem was solved by switching the solvent to methanol. A series of 6 - 8 Hz couplings in the C18-C20 region immediately indicated that this ring did not adopt a chair conformation. A twist boat type structure satisfied the observed NOE correlations; in particular a strong NOE between H13/H20 that could not be accounted for by any other conformation. This correlation also served to place the C20 hydroxyl group on the top face of the ring in an *R* configuration the same as that in **2** and **1**. H19 exhibited a strong NOE to H18b and a weak one to H18a suggesting that the C19

hydroxyl was also R configured. This agreed with the observed coupling constants in this region all of which indicate torsion angles of around 30˚ or 150˚, see Figure S32.

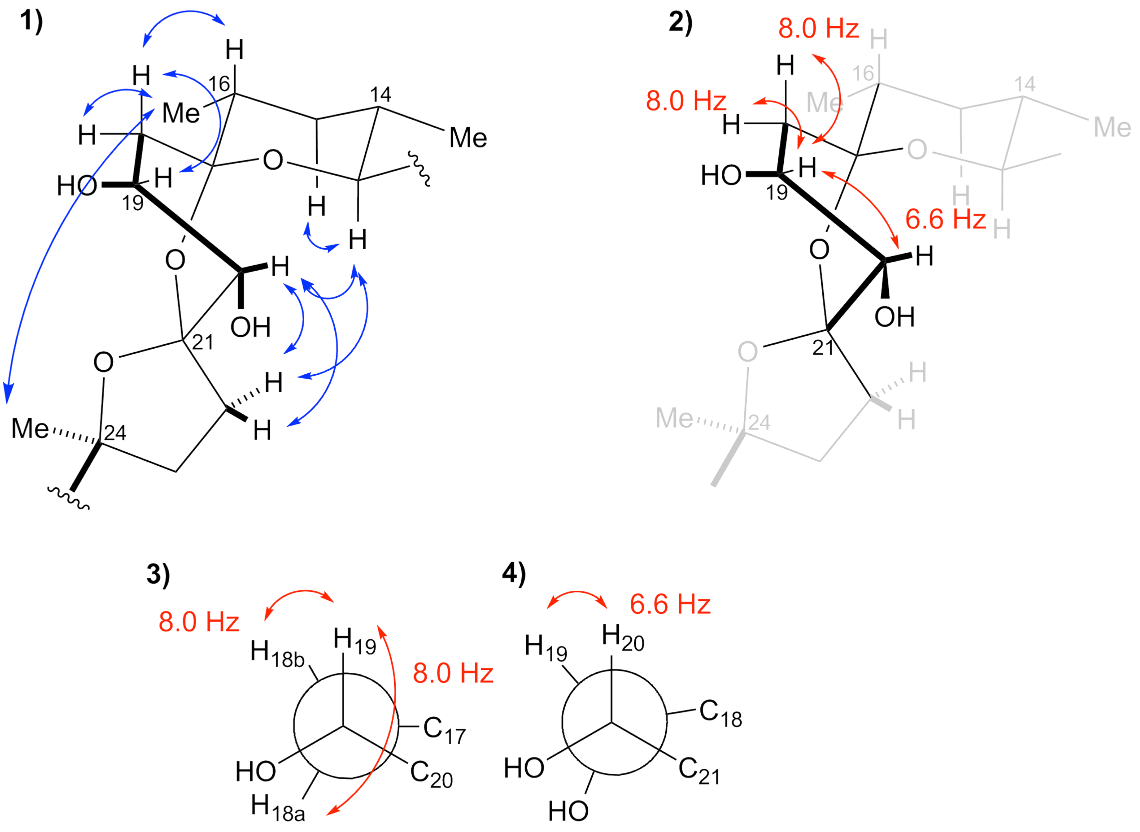

Figure S32. NMR analysis of *bis*-spiroacetal ring system in 2. 1 - Proposed conformation of the BCD ring system showing key NOE correlations. 2 - Key coupling constants in the C18-C20 region. 3 - Conformation about the C18-C19 bond. 4 - Conformation about the C19-C20 bond.

The possibility of the C19 hydroxyl being S configured cannot be entirely ruled out as no NOE was observed between H19/H20. This configuration however would be expected to lead to a large (8- 12 Hz) and a small (0-4 Hz) coupling constant for H19-H18a/b which does not fit with the observed 8 Hz coupling observed. The absence of the H19-H20 NOE can possibly be rationalised by the proximity of the H19 and H20 signals meaning that the NOE cross peak could be obscured by the diagonal. A degree of flexibility in this region is also possible and the observed coupling constants could reflect the existence of multiple conformers although this is thought to be unlikely.

As with **3** an NOE correlation between H20/H22a and H20/H22b placed C22 on the same face of the C ring as H20 and confirmed the configuration at C21 as being the same as in **1**. A further NOE between H33 and H34 confirmed the configuration of C24 in **2**, again the same as that in **1.**

Stereochemistry of the A ring

Analysis of this region was hampered somewhat by the convergence of the H4a and H5a protons meaning NOE's to these protons could be due to either or both. It is proposed that both are oriented axially and the NOE's observed are due to those shown in Figure S33. A series of NOE correlations between H2/H5a/H7 served to place these substituents in axial orientations on the bottom face of the ring. This implies a *trans* relationship across the tetrahydropyran oxygen between the C3 and C7 substituents. NOE's between H6/H7 and H4a/H40 along with the lack of an NOE between H7/H40 suggested that the C6 methyl group was axially oriented and thus the configuration of this ring is indeed the same as for **1**. Analysis of the coupling constants that could be determined suggested that the $^3J_{H3\text{-}H2}$ coupling constant was 11.0 Hz in agreement with the conformation shown in Figure S33 where H2 is oriented into the ring and is hence *anti* to H3. H3 appears as a broadened doublet of doublets (J = 11.0, 4.4 Hz), so the H3-H4 coupling constants must both be small, again consistent with the equatorial orientation of H3.

H7 showed two coupling constants (10.1, 2.0 Hz), and the H6-H7 coupling was assigned as 2.0 Hz based upon the NOE's shown in Figure S33 and the appearance of H6 in rows extracted from TOCSY and NOESY spectra, a narrow multiplet with no large couplings apparent.

Comparison with the NOE's and coupling constants observed in the spectra for a sample of authentic **1** showed good agreement with the observed data for **2** and **3** supporting the conclusion that the configuration of the A ring is unchanged.

The C2 configuration is also assumed not to have changed from the natural product. The H2-H3 coupling constant is almost identical for **1** and both **2** and **3**. This would be expected to change should the C2 configuration be different, as the conformation about this bond would be different in order for the C1 carboxylic acid to participate in intramolecular hydrogen bonding.

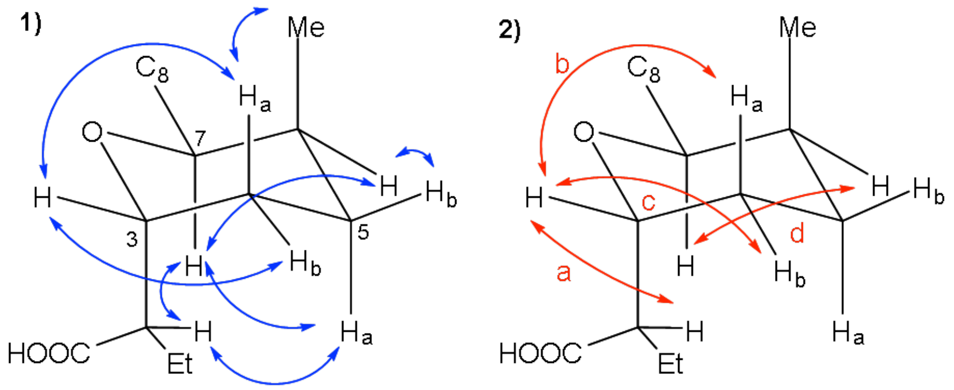

Figure S33. Conformation of the A ring. 1 - Key NOE correlations (observed in both **2, 3,** and **1**). 2 - Selected coupling constants in CD_3CN (numbers are given in Table S9).

Table S9. Selected coupling constants in CD_3CN in the A ring of 1, 2, 3.

**Bond**	$^3J_{H\text{-}H}$ **2** (Hz)	$^3J_{H\text{-}H}$ **3** (Hz)	$^3J_{H\text{-}H}$ **1** (Hz)
a) H2-H3	11.0	10.8	11.0
b) H3-H4a[a]	0[b]	0[b]	0[b]
c) H3-H4b[a]	4.4	4.6	4.7
d) H6-H7	2.0	0[b]	2.2

[a] Assignments interchangeable. [b] Too small to be resolved

Stereochemistry of the E ring

A series of NOE correlations H25/H27a/H30 helped to place all these protons on the same face of the E ring and confirm the configuration of the C25 and C29 stereocentres. The axial orientation of H25 was confirmed by a large (10.5 Hz) coupling to H26a. NOE's from H30 to H31 and H32 implied the C28 ethyl substituent was on the bottom face in an equatorial position and thus the E ring had the same configuration as for **1**. Comparison with the spectra for an authentic sample again showed all these NOE correlations to be present (Figure S34).

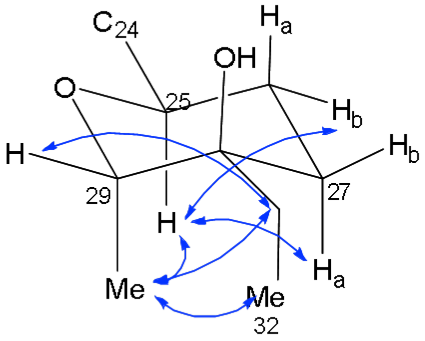

Figure S34. Conformation of the E ring showing key NOE correlations (observed in both **2, 3,** and **1**).

Stereochemistry of the C7-C13 region

Comparison of the NOE correlations and coupling constants in this region with an authentic sample of **1** helped determine that the stereochemistry of **2** and **3** was unchanged with respect to the natural product.

Conformations about each bond were determined based on the observed coupling constants and NOE correlations as shown in Figure S35 and were compared to an authentic sample of **1**. While the data do not rule out the existence of other conformers or configurations the good match for the data observed for **1**, particularly the coupling constant data, suggests that the configuration in this region is the same.

The strong hydrogen bonding network present in **1** is the dominant factor in determining its conformation. As a result if the configuration of one of the centres were to have changed the

conformation of that region is expected to change in order to maintain the hydrogen bonding network, and this would result in a significant change in the coupling constants and NOE's observed in this region.

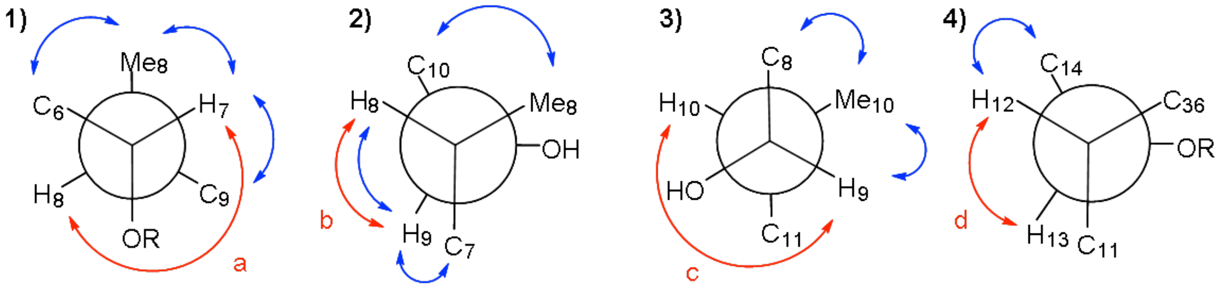

Figure S35. NOE correlations (blue) (observed in both 2, 3, and 1) and coupling constants (red) in the C7-C10 region. 1 - Conformation about the C7-C8 bond. 2 - Conformation about the C8-C9 bond. 3 - Conformation about the C9-C10 bond. 4 - Conformation about the C12-C13 bond. (The numbers are given in Table S10).

Table S10. Selected coupling constants in CD_3CN in the C7-C10 region of 1, 2, 3.

**Bond**	$^{3}J_{H\text{-}H}$ **2** (Hz)	$^{3}J_{H\text{-}H}$ **3** (Hz)	$^{3}J_{H\text{-}H}$ **1** (Hz)
a) H7-H8	10.1	8.7	10.1
b) H8-H9	0^{a}	0^{a}	0^{a}
c) H9-H10	9.7	9.8	10.0
d) H12-H13	0^{a}	0^{a}	0^{a}

[a] Too small to be resolved

Table S11. NMR data for 2.

Atom	δ_c (ppm)[a]		δ_H (ppm) CD_3CN[b, c]		δ_H (ppm) CD_3OD[b]		COSY correlations	Spin System	HMBC correlations[c, d]	NOESY correlations[c, e]
1	178.45	C							2, 3, 4a (w), 41	
2	48.59	CH	3.01	td (10.0, 6.0)	3.04	brs	3, 41a, 41b	A	3, 4a, 4b/41, 42	3, 4a/5a, 7, 41, 42
3	75.87	CH	3.92	dd (11.0, 4.4	4.00	dd (10.3, 5.5)	2, 4a, 4b	A	2, 4a/5a, 4b/5b/41, 7	2, 4a/5a, 4b
4a	20.47	CH_2	1.90[f]	m	1.96	m	3, 4b/5b	A	2, 3, 5a	2, 4b/5b, 7, 40
4b			1.44[f]	m	1.54	m	4a/5a	A		3, 4a/5a
5a	26.58	CH_2	1.90[f]	m	1.94	m	4b/5b	A	3, 4a, 4b, 7, 40	2, 4b/5b, 7, 40
5b			1.46[f]	m	1.53	m	4a/5a	A		4a/5a, 6
6	28.71	CH	1.83	m	1.87	m	7, 40	A	4a/5a, 4b/5b, 40	4b/5b, 7, 39, 40
7	72.50	CH	3.62	dd (10.1, 2.0)	3.75	brd (9.9)	6, 8	A	3, 5b, 8, 9, 40, 39	2, 4a/5a, 6, 8, 9, 39
8	37.11	CH	1.47	m	1.54	m	7, 39	A	7, 9, 10, 39	9, 38, 39
9	68.01	CH	4.30	d (9.7)	4.23	d (10.1)	10	B	7, 8 (w), 10, 38, 39	7, 8, 10, 20, 38
10	50.20	CH	2.61	dq (9.0, 7.2)	2.89	qd (7.9, 6.8)	9, 38	B	9, 12, 38	10, 12, 13, 38, 39
11	212.98	C							9, 10, 12, 13, 36a, 36b, 38	
12	54.38	CH	2.66	dd (11.0, 1.8)	2.76	dd (9.7, 2.6)	13, 36a, 36b	C	10, 13, 14, 36a, 36b, 37	10, 13, 14, 35, 36a, 36b
13	73.16	CH	3.52	d (10.4)	3.66	d (10.4)	12, 14	D	12, 14, 15a, 15b, 18, 35	12, 14, 15b, 20, 22b, 35
14	33.08	CH	1.64	m	1.72	m	15b, 35	D	12, 13, 15a, 15b, 16, 35	12, 13, 16, 35,
15a	26.58	CH_2	1.39	m	1.43	m	15b, 16	D	13, 14, 16, 34, 35	15b, 35
15b			1.28	q (13.0)	1.36	m	14, 15a, 16	D		13, 15a, 34,
16	39.41	CH	1.48	m	1.57	m	15a, 15b, 34	D	14, 15a, 15b, 18, 34	14, 18, 34
17	99.31	C							12, 13, 15a, 15b, 16, 18, 20 (w), 22a (w)	
18a	40.13	CH_2	2.05	d (8.0)	2.23	dd (12.0, 8.0)	19	E	16, 19, 20	18b, 19 (w), 34[g]
18b			2.05	d (8.0)	1.98	dd (12.0, 7.5)	19	E		16, 18a, 19[g]
19	64.37	CH	4.04	q (7.1)	4.16	brs	18, 20	E	18, 20	18
20	68.57	CH	3.97	d (6.6)	3.99	brs	19	E	18, 19, 22a, 22b	13, 18, 22a, 22b
21	110.34	C							16 (w), 19, 20(w), 22a, 22b, 23a, 23b	
22a	35.43	CH_2	2.45	dt (13.3, 8.6)	2.34	m	22b, 23a, 23b	F	20, 23a, 23b	20, 22b, 23a, 25
22b			1.82	m	2.06	ddd (12.9, 8.2, 4.7)	22a, 23a, 23b	F		20, 22a, 23b,
23a	32.03	CH_2	2.09	m	2.21	m	22a, 22b, 23b	F	22a, 22b, 25, 33	22a, 22b, 23b, 25
23b			1.64	m	1.77	m	22a, 22b, 23a	F		23a, 33
24	87.05	C							20 (w), 22a, 22b, 23a, 23b, 25, 33	
25	71.29	CH	3.50	d (10.5)	3.56	brd (11.1)	26a, 26b	G	23a, 23b/26a/27a, 27b, 29, 33	22a, 23a, 27a, 26b, 30, 33
26a	22.24	CH_2	1.59	m	1.78	m	25, 27b, 26b	G	25 (w), 27a, 27b,	
26b			1.40	m	1.49	m	27a/26a, 27b	G		25, 33
27a	30.09	CH_2	1.59	m	1.65	m	27b, 26b	G	25, 26b, 31	25
27b			1.50	m	1.34	m	26a/27a, 26b	G		
28	71.29	C							27b, 26b, 29, 30, 31, 32	
29	78.03	CH	3.70	q (7.0)	3.84	brs	30	H	25, 27b, 30, 31	25 (w), 30, 31, 32
30	14.89	CH_3	1.16	d (7.0)	1.23	d (6.9)	29	H	29	25, 29, 31, 32
31	31.92	CH_2	1.27	m	1.36	m	32	I	33, 32	29, 30, 32
32	6.69	CH_3	0.83	t (7.5)	0.93	m	31	I	31	29, 31
33	25.05	CH_3	1.22	s	1.35	m		J	23a, 23b, 25	23b, 25, 34
34	16.98	CH_3	0.84	d (6.6)	0.92	m	16	D	16, 15a, 15b	15b, 16, 18, 30, 33
35	18.11	CH_3	0.86	d)6.6)	0.93	m	14	D	14, 15a, 15b	13, 14, 15a,
36a	17.57	CH_2	1.81	m	1.90	m	12, 36b, 37	C	12, 13, 37	12, 13, 36b, 37
36b			1.32	m	1.46	m	12, 36a, 37	C		12, 36a, 37
37	13.21	CH_3	0.74	t (7.5)	0.87	t (7.4)	36	C	12, 36a, 36b	12, 36a, 36b
38	13.35	CH_3	0.79	d (7.2)	0.89	m	10	B	9 (w), 10	9, 8, 10, 12,
39	7.67	CH_3	0.70	d (6.8)	0.80	d (6.9)	8	A	7, 8, 9	6, 7, 8, 40
40	11.62	CH_3	0.94	d (6.8)	0.99	d (6.7)	6	A	7, 5a, 5b (w), 6 (w)	4a/5a, 6, 39
41a	23.34	CH_2	1.44	m	1.46	m	2, 42	A	2, 3, 42	2, 42
41b			1.40	m			2, 42	A		2, 42
42	12.28	CH_3	0.93	t (7.7)	0.98	t (6.6)	41a, 41b	A	2, 41	2, 41

[a] Measured in CD_3CN. [b] ^1H data reported as δ_H (ppm), multiplicity (coupling constants in Hz), δ_H values for signals listed as multiplets were determined from the HSQC spectrum. [c] (w) denotes a weak coupling. [d] HMBC correlations are listed for protons showing a correlation to that carbon. [e] In CD_3CN unless stated. [f] Protons 4a/5a and 4b/5b could not be resolved and cross peaks are potentially due to either or both protons. [g] In CD_3OD as signals converged in CD_3CN.

Table S12. NMR data for 3.

Atom	δ_C (ppm)		δ_H (ppm)[a]		COSY correlations[b]	Spin system	HMBC correlations[b, c]	NOESY correlations[b]
1	177.92	C					2, 41	
2	50.16	CH	2.85	td (9.9 5.3)	3, 41	A	3, 4a, 4b, 41, 42	3, 7, 5a, 41, 4b, 42
3	75.19	CH	3.85	dd (10.8, 4.6)	2, 4a, 4b	A	2, 4a/5a, 41	2, 4a/5a, 4b, 41, 42
4a[d]	20.75	CH_2	1.90	m	4b, 5b	A	2, 3, 5a, 5b, 6	2, 3, 4b, 5b, 6, 7, 40
4b			1.37	m	4a, 5a, 5b	A		2 (w), 3, 4a/5a, 7
5a[d]	26.85	CH_2	1.90	m	4b, 5b	A	3, 4a, 4b, 6, 7, 40	2, 3, 4b, 5b, 6, 7, 40
5b			1.48	m	4a, 4b, 5a	A		4a/5a, 6
6	28.63	CH	1.77	m	5a, 5b (w), 7, 40	A	4a/5a, 4b, 5b, 8, 40	4a/5a, 5b, 7, 39, 40
7	72.78	CH	3.55	d (8.7)	6, 8	A	3, 8, 9, 49, 40	2, 4b, 5a, 7, 41, 42
8	37.07	CH	1.43	m	7, 39	A	7, 9, 39	7, 9, 10 (w), 38, 39
9	69.42	CH	3.99	d (9.8)	10	B	7 (w), 10, 38, 39	7, 8, 10, 13, 38, 39 (w)
10	52.36	CH	2.72	dq (9.7, 7.1)	9, 38	B	9, 12, 38	8, 9, 12, 38, 39
11	216.93	C					9, 10, 12, 13, 36, 36b, 38	
12	56.02	CH	2.75	dd (11.1, 2.9)	13 (w), 36a, 36b	C	10, 13, 14, 36a, 36b, 37	10, 13, 14, 35, 36a, 36b, 37
13	75.78	CH	3.73	d (10.5)	12 (w), 14	D	12, 15a, 15b, 35, 36b	12, 14 (w), 15b, 18a, 35
14	33.41	CH	1.63	m	13, 15b, 35	D	21, 13, 15a, 15b, 35	12, 13, 35, 36b
15a	38.32	CH_2	1.59	m	15b	D	13, 14, 16, 34, 35	15b, 34, 35
15b			1.02	q (13.0)	14, 15a, 16	D		13, 15a, 18a
16	41.61	CH	1.56	m	15b, 34	D	15a, 15b, 34	34
17	102.84	C					12, 13 (w), 15b, 16, 18a, 18b, 19, 34	
18a	26.94	CH_2	2.28	dd (14.7, 3.9)	18b, 19	E	16, 19	13, 15b, 18b, 19
18b			1.59	dd (14.9, 3.5)	18a, 19	E		18a, 19, 20
19	68.97	CH	3.92	brs	18a, 18b, 20	E	18a, 18b	18a, 18b, 20
20	71.38	CH	3.40	brs	19	E	18a, 18b, 22a, 22b	18b, 19, 22a, 22b (w)
21	109.70	C					19, 20 (w), 22a, 22b, 23b	
22a	38.51	CH_2	2.29	m	22b, 23a, 23b	F	23a, 23b	20, 22b, 23b
22b			1.99	m	22a, 23a, 23b	F		20, 22a, 23b
23a	31.34	CH_2	2.13	m	22a, 22b, 23b	F	22a, 22b, 33	23b, 26b, 33
23b			1.63	m	22a, 22b, 23a	F		22a, 22b, 23a, 33
24	89.31	C					22a, 22b, 23b, 25, 33	
25	74.76	CH	3.73	d (8.7)	26a, 26b	G	26b, 29, 33	26b, 27a, 33, 30
26a	22.50	CH_2	1.66	m	25, 26b	G	27a, 27b	
26b			1.54	m	25, 26a	G		23a, 25
27a	30.02	CH_2	1.68	m		G	25, 26b (w), 31	25, 30, 31
27b			1.58	m		G		31
28	71.43	C					29, 30, 31, 32	
29	78.01	CH	3.77	q (6.5)	30	H	27b, 30, 31	30, 31, 32
30	15.13	CH_3	1.28	d (7.0)	29	H	29	29, 25, 27a
31	31.77	CH_2	1.33	q (7.4)	32	I	32	27a, 27b, 29
32	6.72	CH_3	0.87	t (7.8)	31	I	31	29
33	25.36	CH_3	1.37	s		J	22a, 23b	23a, 23b, 25
34	16.70	CH_3	0.78	d (6.7)	16	D	15a, 15b, 16	15a, 16, 20
35	18.06	CH_3	0.88	d (6.4)	14	D	14, 15a, 15b	12, 13, 14, 15a
36a	18.15	CH_2	2.00	m	12, 37	C	12, 13, 37	12, 36b, 37
36b			1.38	m	12, 37	C		12, 14, 36a, 37
37	13.24	CH_3	0.77	t (8.2)	36a, 36b	C	12, 36a, 36b	12, 13, 20, 36a, 36b
38	13.27	CH_3	0.74	d (7.2)	10	B	9 (w), 10	8, 9, 10
39	7.23	CH_3	0.68	d (6.9)	8	A	8, 9	6, 7, 8, 9, 10, 40
40	11.28	CH_3	0.92	d (7.0)	6	A	5a, 5b, 6, 7	4a/5a, 6, 7
41	24.11	CH_2	1.44	m	2, 42	A	2, 3, 42	2, 3, 42
42	12.32	CH_3	0.91	t (7.4)	41	A	2, 41	2, 3, 41

All data recorded in CD_3CN. [a] ^1H data reported as δ_H (ppm), multiplicity (coupling constants in Hz), δ_H values for signals listed as multiplets were determined from the HSQC spectrum. [b] (w) denotes a weak coupling. [c] HMBC correlations are listed for protons showing a correlation to that carbon. [d] Protons 4a and 5a could not be resolved and cross peaks are potentially due to either or both protons.

Table S13. NMR data for 1.

Atom	δ_C (ppm)	δ_H (ppm)[a]		NOESY correlations[b]
1	183.94			
2	50.64	2.76	m	3, 4a/5a, 7, 41b, 42
3	76.04	3.79	dd (11.0, 4.7)	2, 4a/5a, 4b, 41a, 41b
4a	19.64	1.85[c]	m	2, 3, 4b, 5b, 40[c]
4b		1.40	m	3, 4a/5a, 41a
5a	26.49	1.86[c]	m	2, 3, 4b, 5b, 40[c]
5b		1.43	m	5a, 40
6	27.94	1.79	m	7, 8, 39, 40
7	71.14	3.63	dd (10.1, 2.2)	2, 4a/5a, 6, 8
8	35.74	1.43	m	6, 7, 9, 38, 39
9	68.34	4.03	m	7, 8, 10, 38, 39
10	48.94	2.82	dq (10.0, 7.0)	9, 12, 38, 39
11	219.94			
12	55.94	2.75	m	10, 13, 14, 36a, 36b
13	75.94	3.70	d (10.1)	10, 12, 14 (w), 15b, 18, 39, 40
14	32.44	1.71	m	13 (w), 35, 36b
15a	38.14	1.69	m	15b, 34, 35
15b		1.18	q (12.3)	13, 15a, 18, 34, 35
16	40.44	1.64	m	34
17	98.94			
18	122.54	5.77	dd (10.9, 1.7)	13, 15b, 19, 20 (w), 34
19	130.04	6.05	d (10.9)	18, 20, 34
20	66.54	4.04	m	18 (w), 19, 22a
21	106.64			
22a	37.34	2.17	m	20, 22b, 23a, 23b
22b		1.97	m	22a, 23a, 23b
23a	32.04	1.88	m	22a, 22b
23b		1.82	m	22a, 22b
24	88.44			
25	74.14	3.52	dd (12.1, 2.5)	23a, 23b, 26a, 26b, 27, 30, 33
26a	19.44	2.21	m	25, 26a, 27, 32
26b		1.36	m	26a, 27
27a	28.64	1.57	m	26a, 26b, 31, 32
28	69.84			
29	76.94	4.03	m	30, 31, 32
30	14.04	1.24	d (6.9)	25, 29,
31	31.94	1.26	m	27, 29, 32
32	5.84	0.88	t (7.5)	26a, 27, 29, 31
33	26.58	1.61	s	23a, 23b, 25
34	15.34	0.70	d (7.3)	15a, 15b, 16, 18, 19,
35	16.84	0.90	d (6.4)	13, 14, 15a, 15b
36a	15.14	1.90	m	12, 36b, 37
36b		1.37	m	36a, 37
37	12.34	0.76	t (7.4)	12, 36a, 36b
38	11.91	0.78	d (7.1)	8, 9, 10, 39
39	6.14	0.70	d (7.3)	6, 7, 8, 9, 10, 38, 40
40	10.24	0.92	d (7.0)	4a/5a 5b, 6, 39
41a	23.14	1.33	m	2, 41b, 42
41b		1.23	m	2, 4b , 41a, 42
42	11.96	0.85	t (7.4)	2, 41a, 41b

All data recoreded in CD_3CN. [a] ^1H data reported as δ_H (ppm), multiplicity (coupling constants in Hz), δ_H values for signals listed as multiplets were determined from the HSQC spectrum. [b] (w) denotes a weak coupling. [c] Protons 4a and 5a overlap and cross peaks are potentially due to either or both protons.

Figure S36. ^1H NMR spectrum of 2. The spectrum was recorded in CD_3CN.

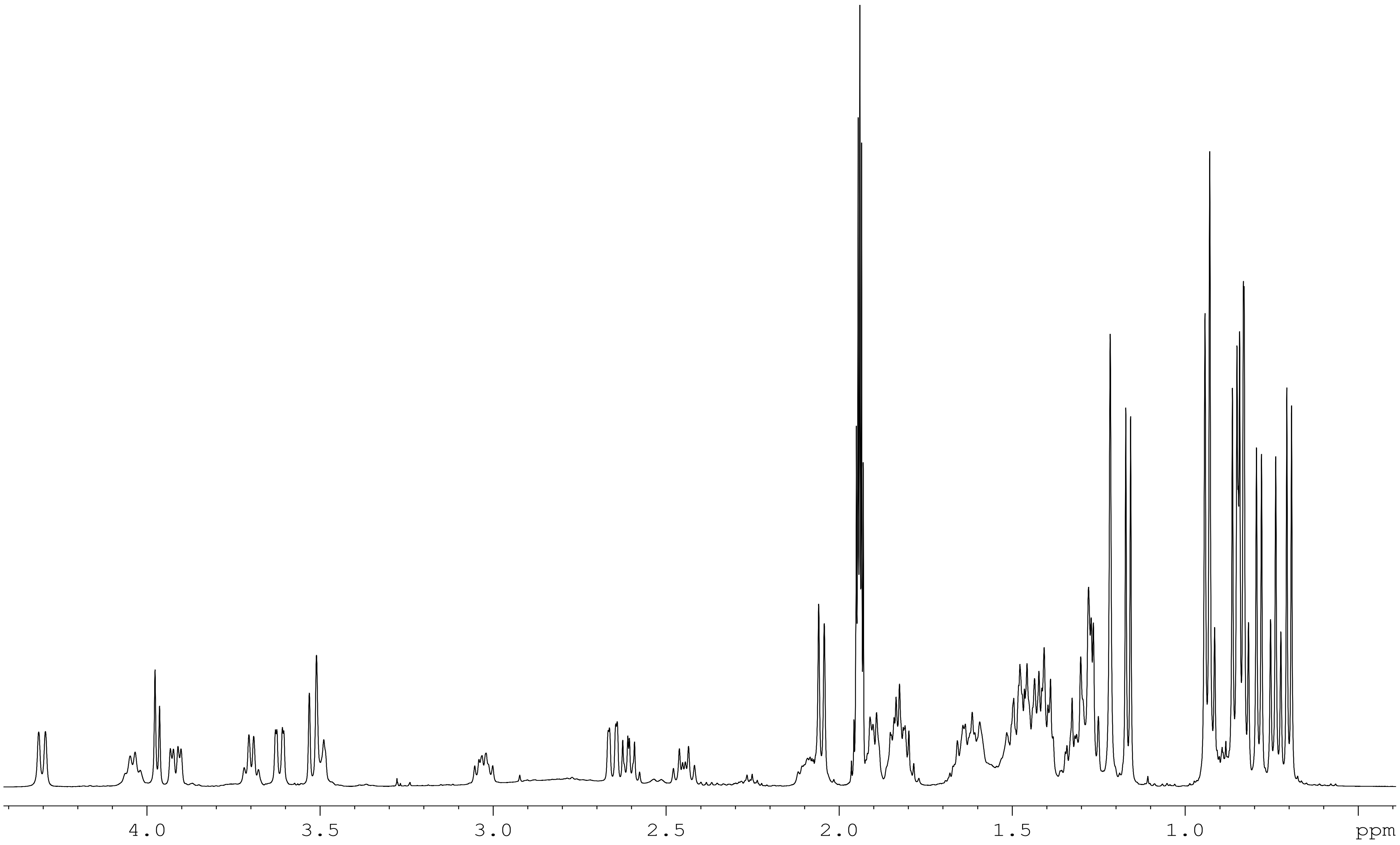

Figure S37. Expansion of the regions between 0.6 ppm and 2.2 ppm, and 2.4 ppm and 4.4 ppm in the ^{1}H NMR spectrum of 2 from Figure S36.

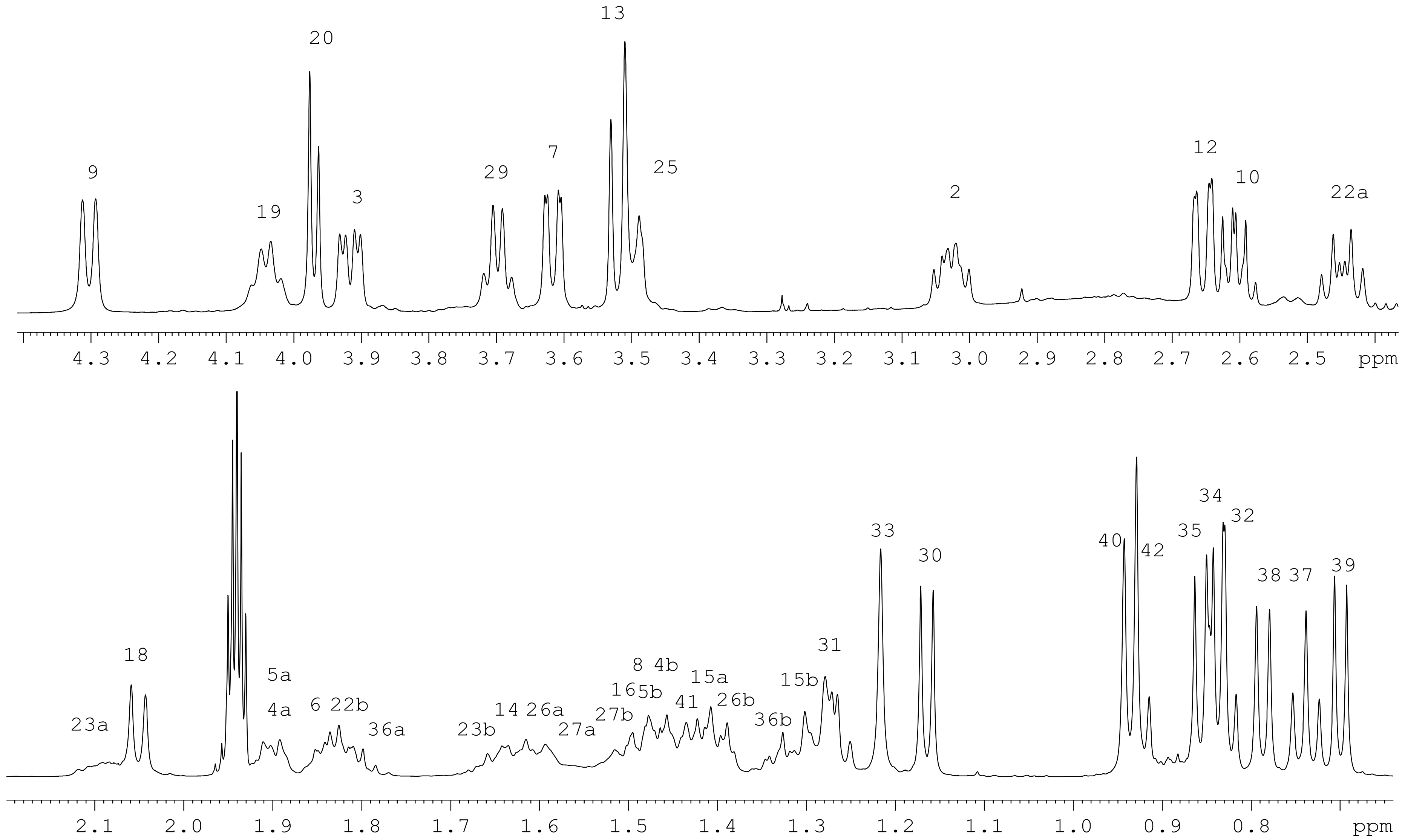

Figure S38. ^{13}C **NMR spectrum of 2. The spectrum was recorded in** CD_3CN**.**

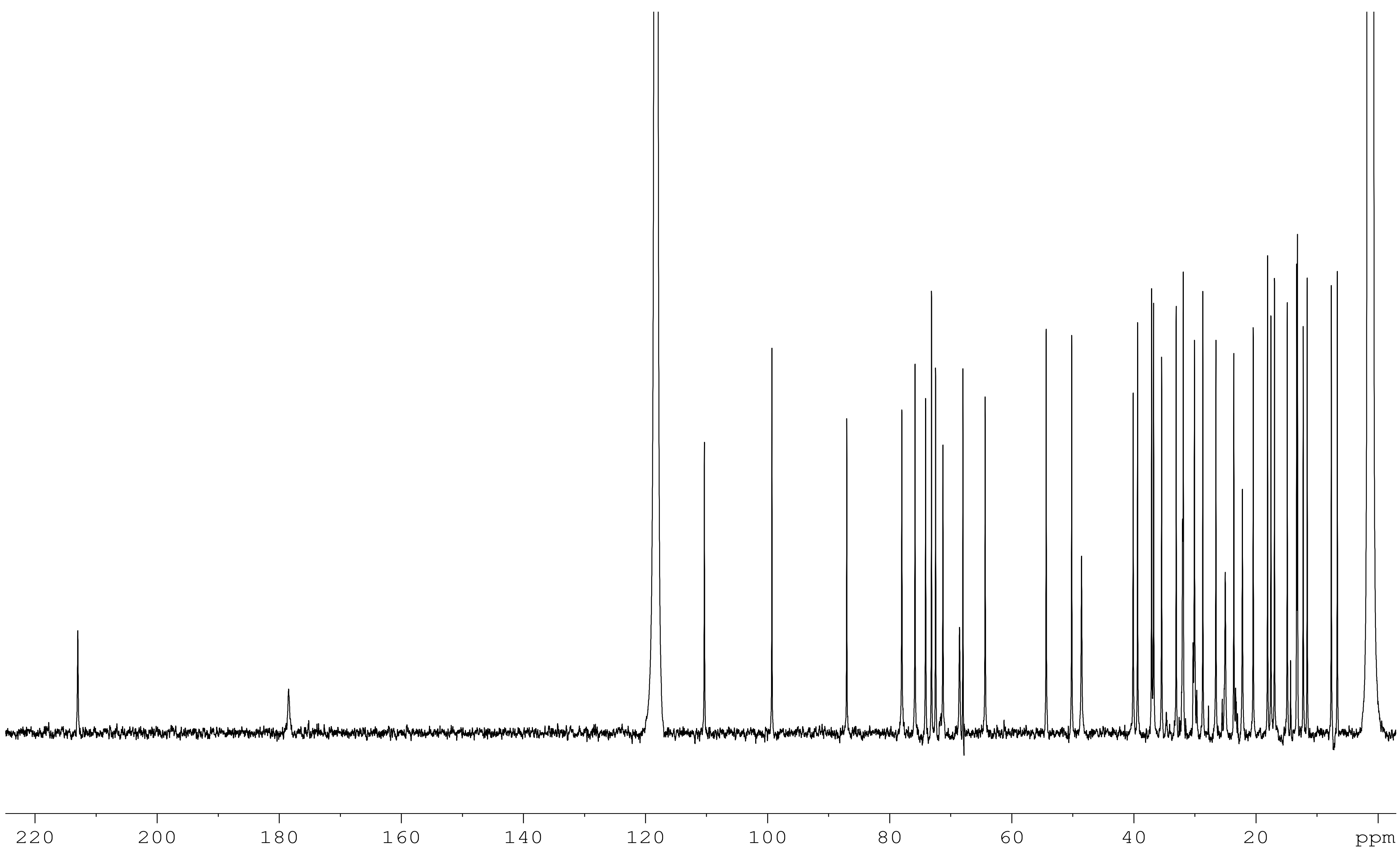

Figure S39. Expansion of the regions between 5 ppm and 45 ppm, and 45 ppm and 220 ppm in the ^{13}C NMR spectrum of 2 from Figure S38.

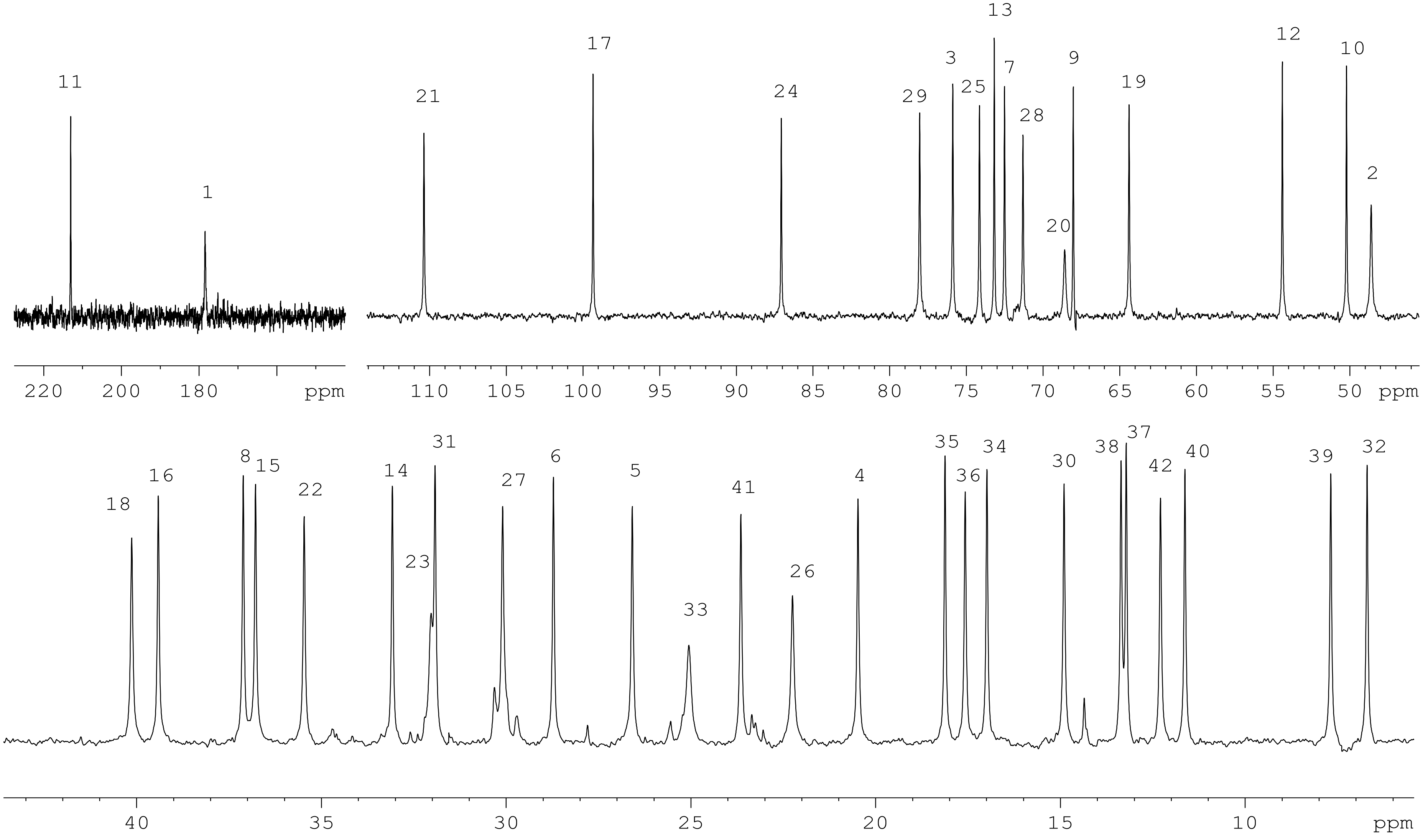

Figure S40. DQF-COSY NMR spectrum of 2. The spectrum was recorded in CD_3CN.

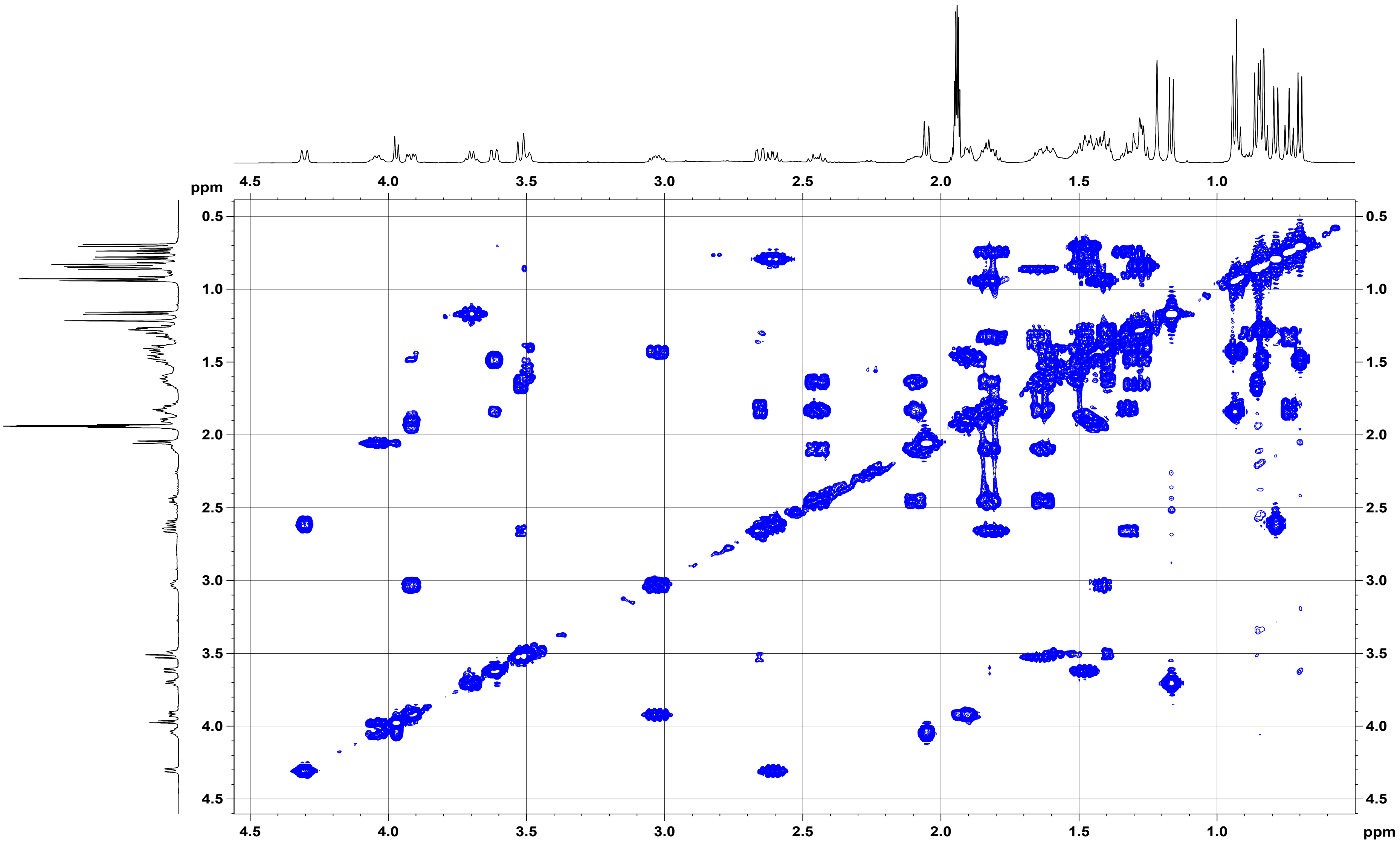

Figure S41. Edited HSQC NMR spectrum of 2. The spectrum was recorded in CD_3CN.

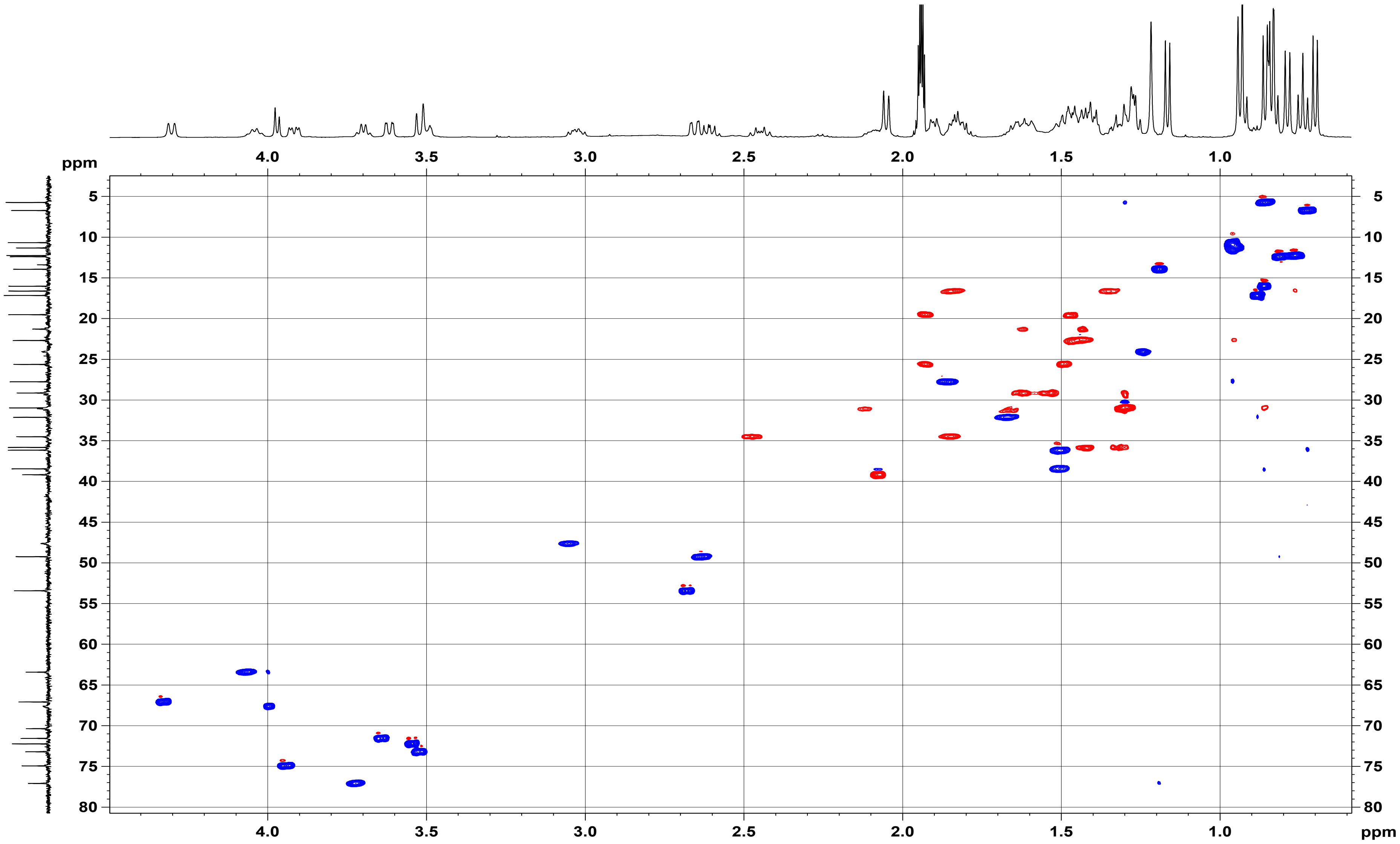

Figure S42. HMBC NMR spectrum of 2. The spectrum was recorded in CD_3CN.

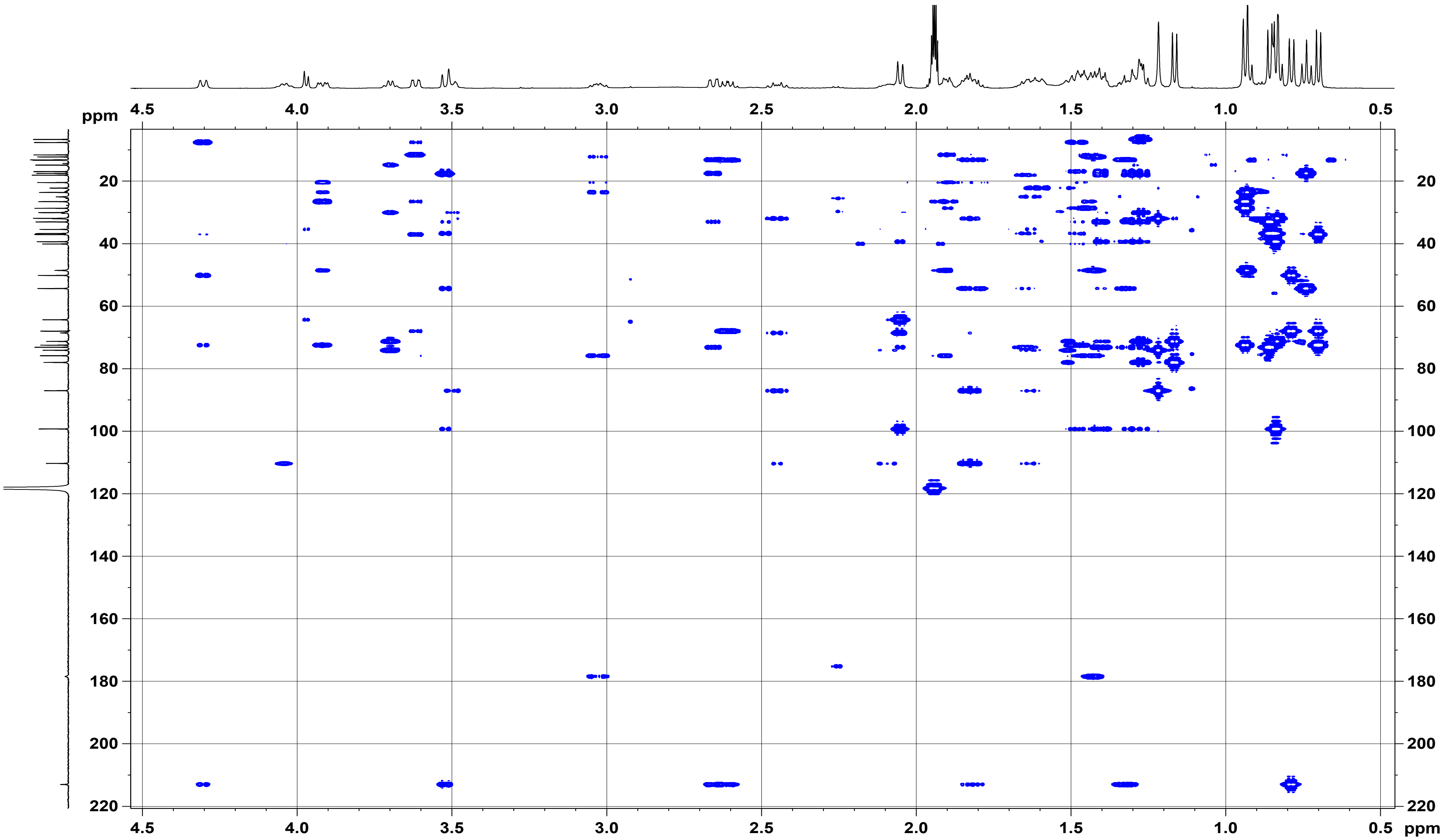

Figure S43. NOESY NMR spectrum of 2. The spectrum was recorded in CD_3CN.

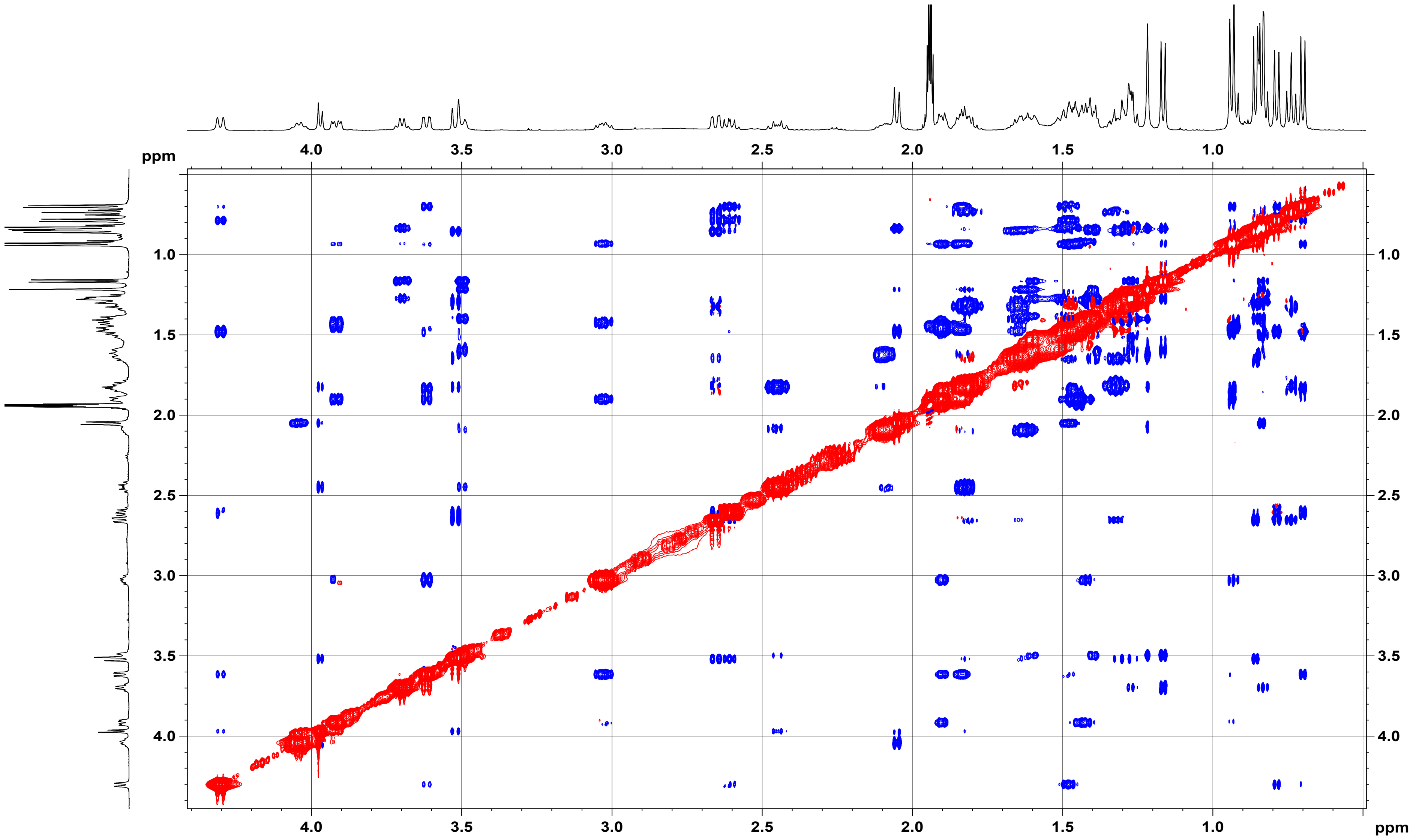

Figure S44. Edited HSQC-TOCSY NMR spectrum of 2. The spectrum was recorded in CD_3CN.

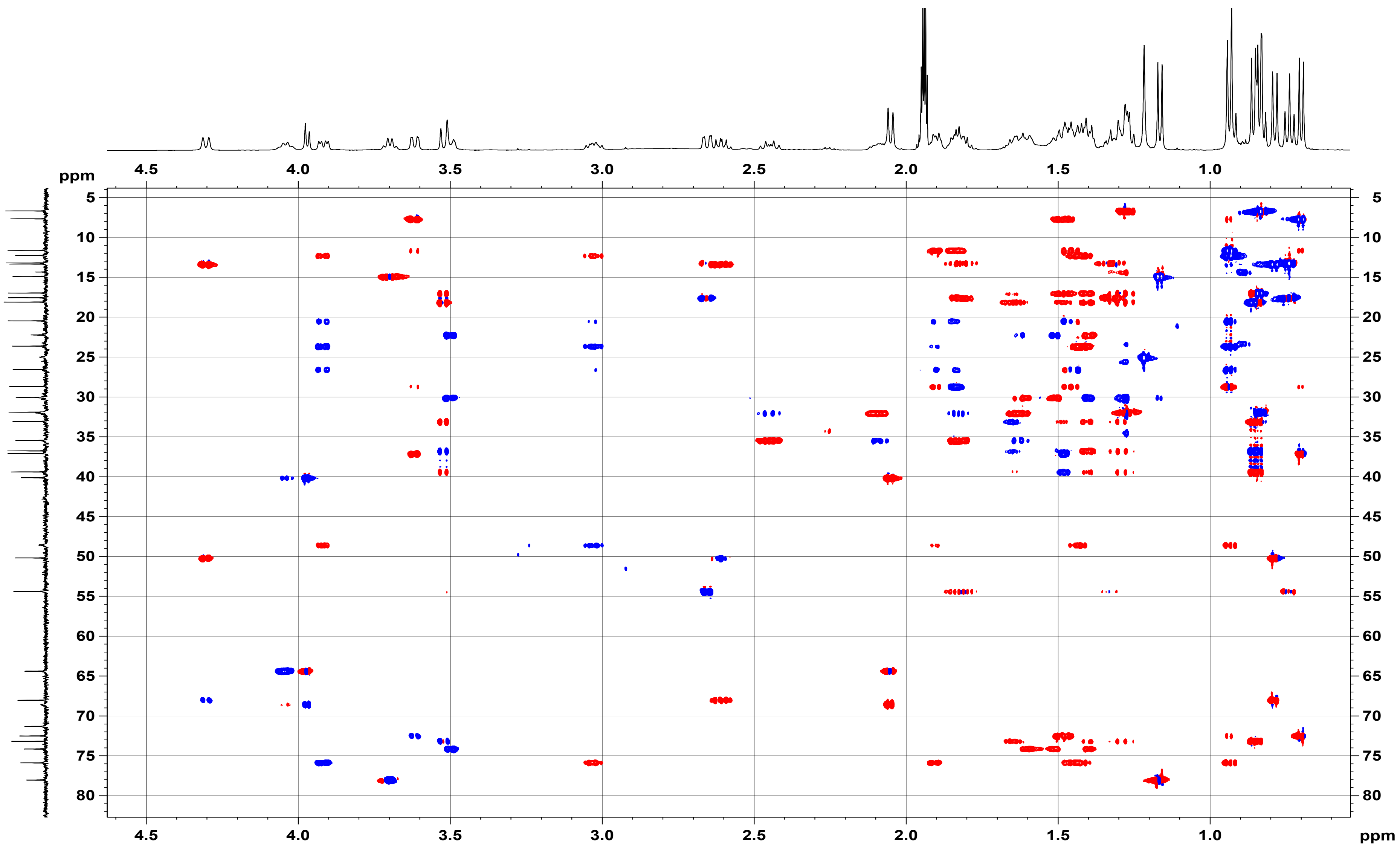

Figure S45. TOCSY NMR spectrum of 2. The spectrum was recorded in CD_3CN.

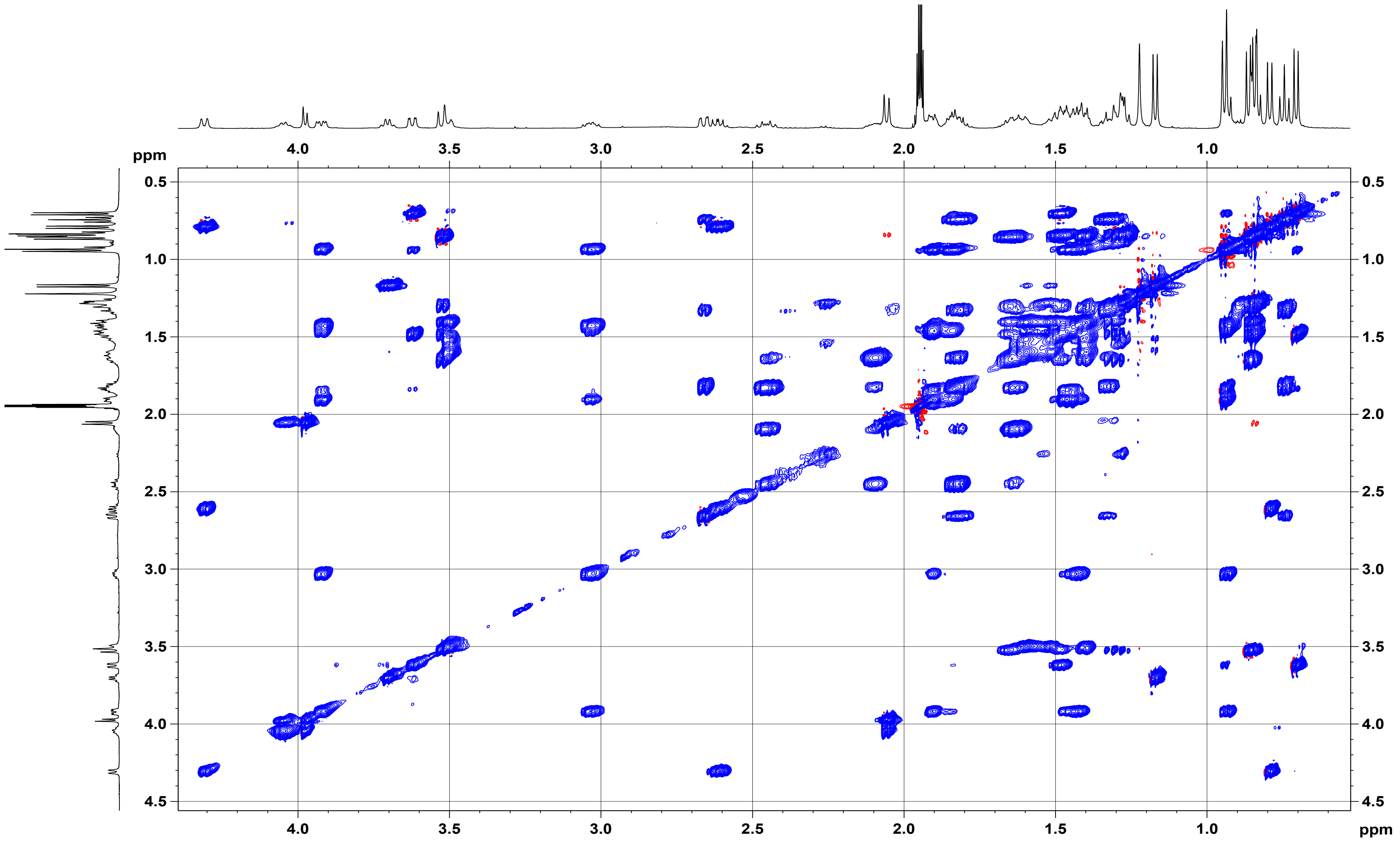

Figure S46. ^{1}H NMR spectrum of 2. The spectrum was recorded in CD_3OD.

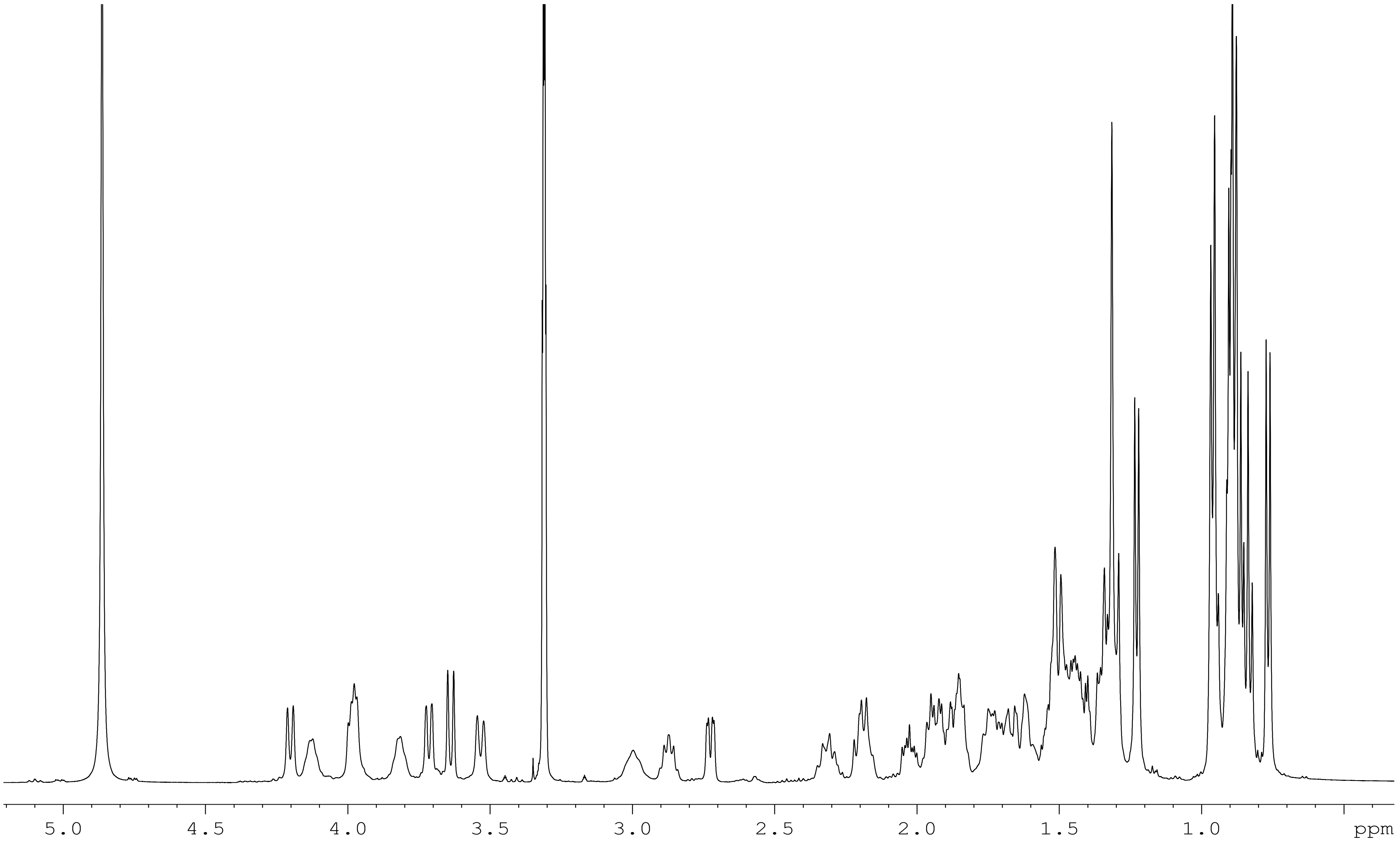

Figure S47. Expansion of the regions between 0.7 ppm and 2.1 ppm, and 2.1 ppm and 4.3 ppm in the ^{1}H NMR spectrum of 2 from Figure S46.

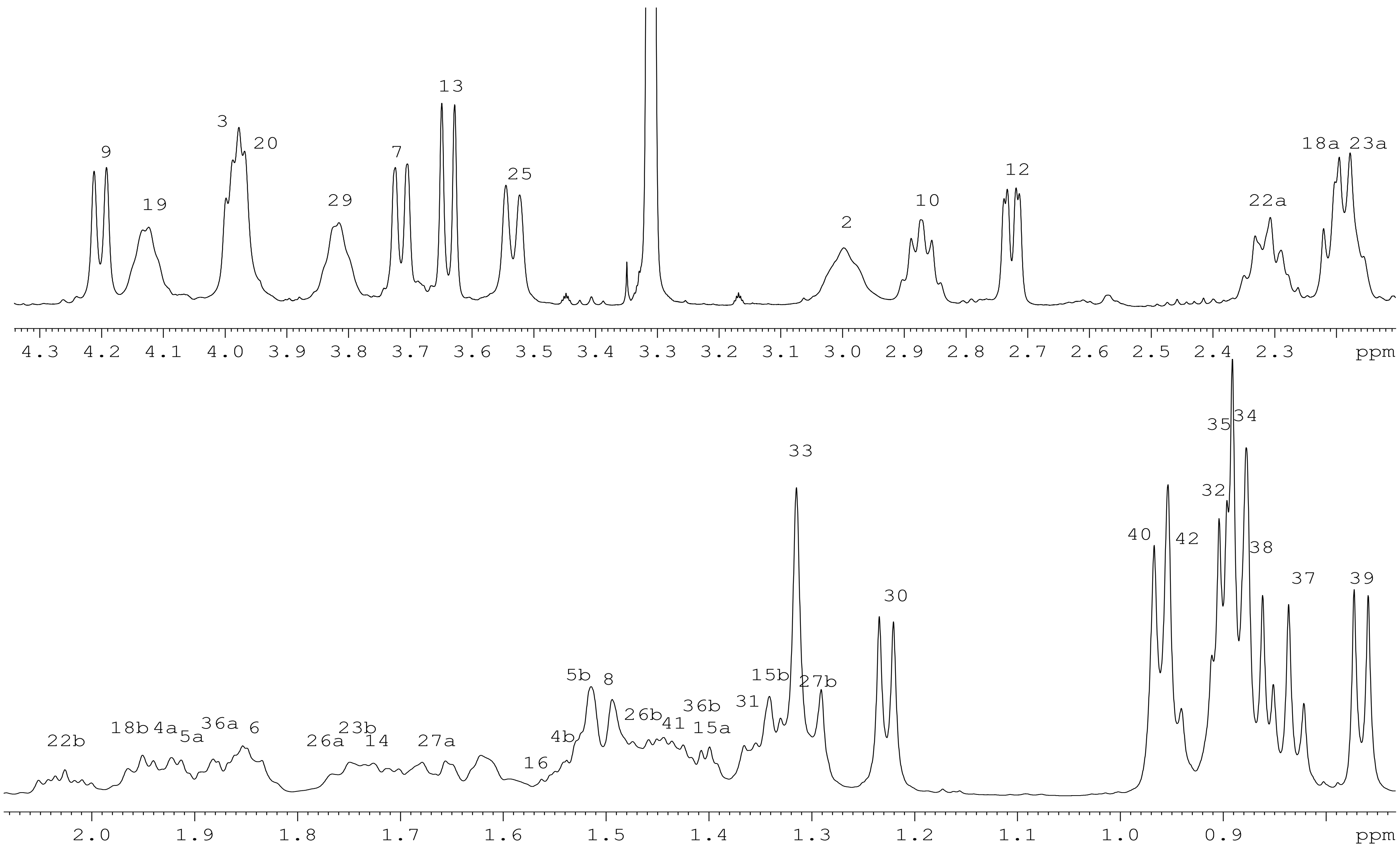

Figure S48. COSY NMR spectrum of 2. The spectrum was recorded in CD_3OD.

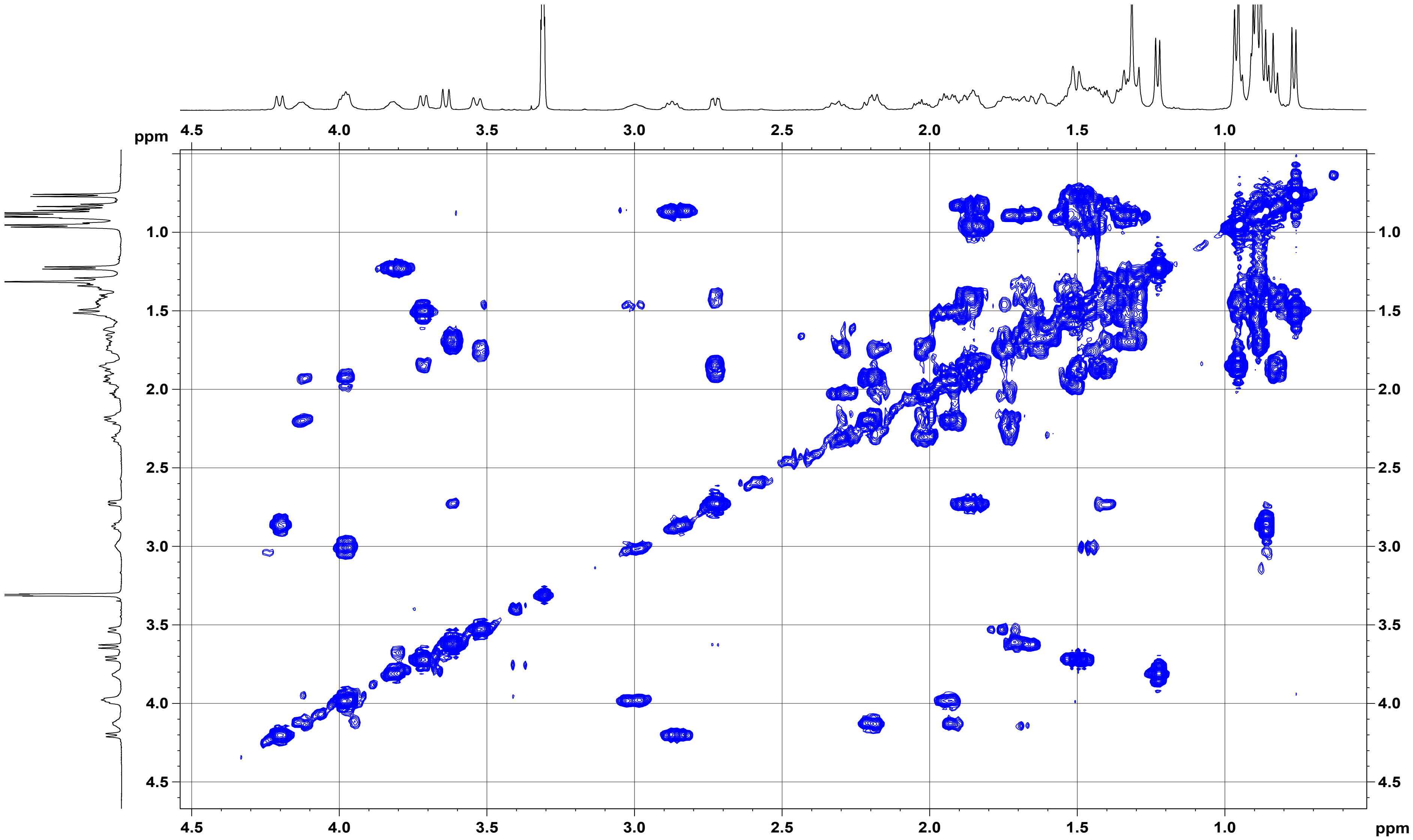

Figure S49. Edited HSQC NMR spectrum of 2. The spectrum was recorded in CD_3OD.

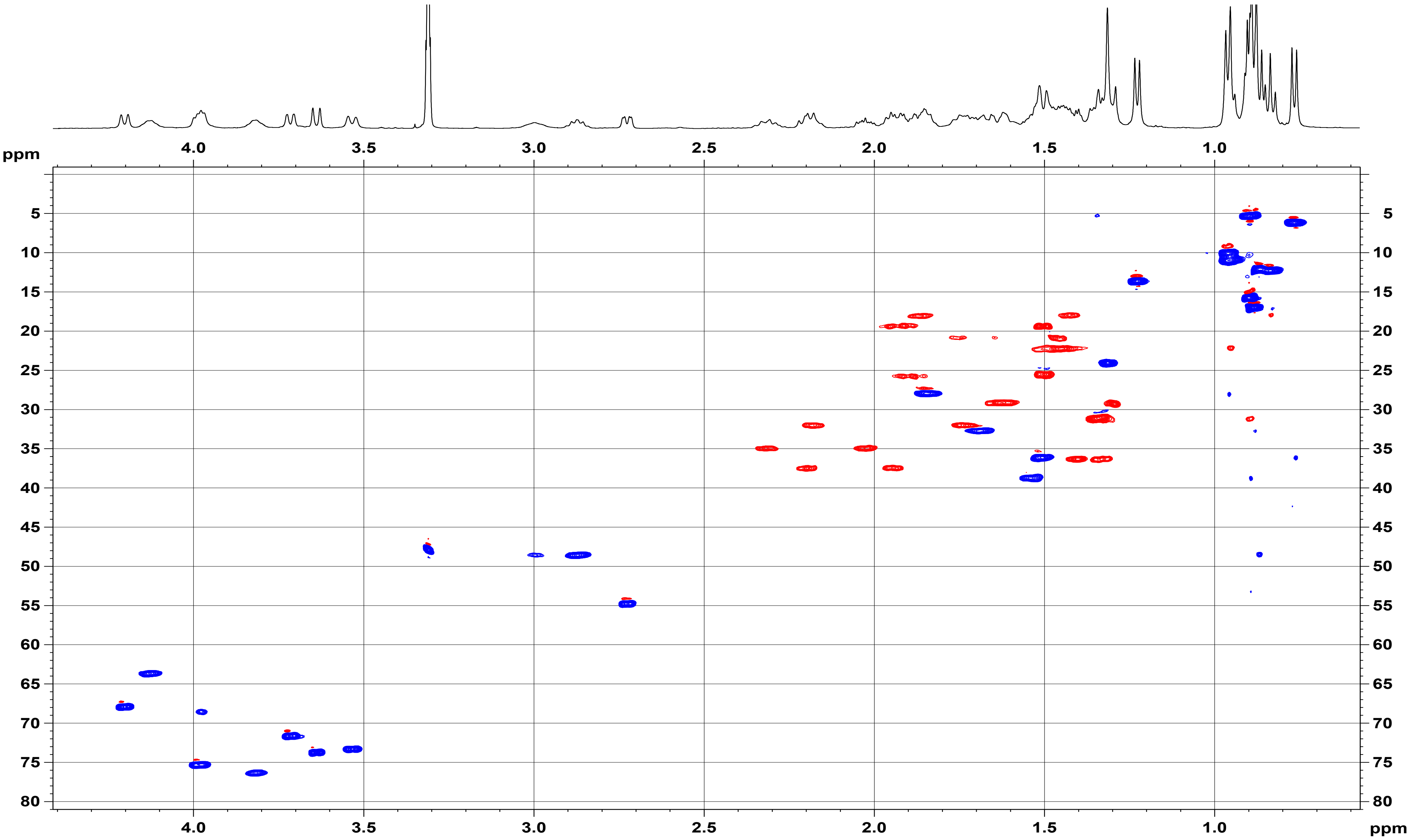

Figure S50. NOESY NMR spectrum of 2. The spectrum was recorded in CD_3OD.

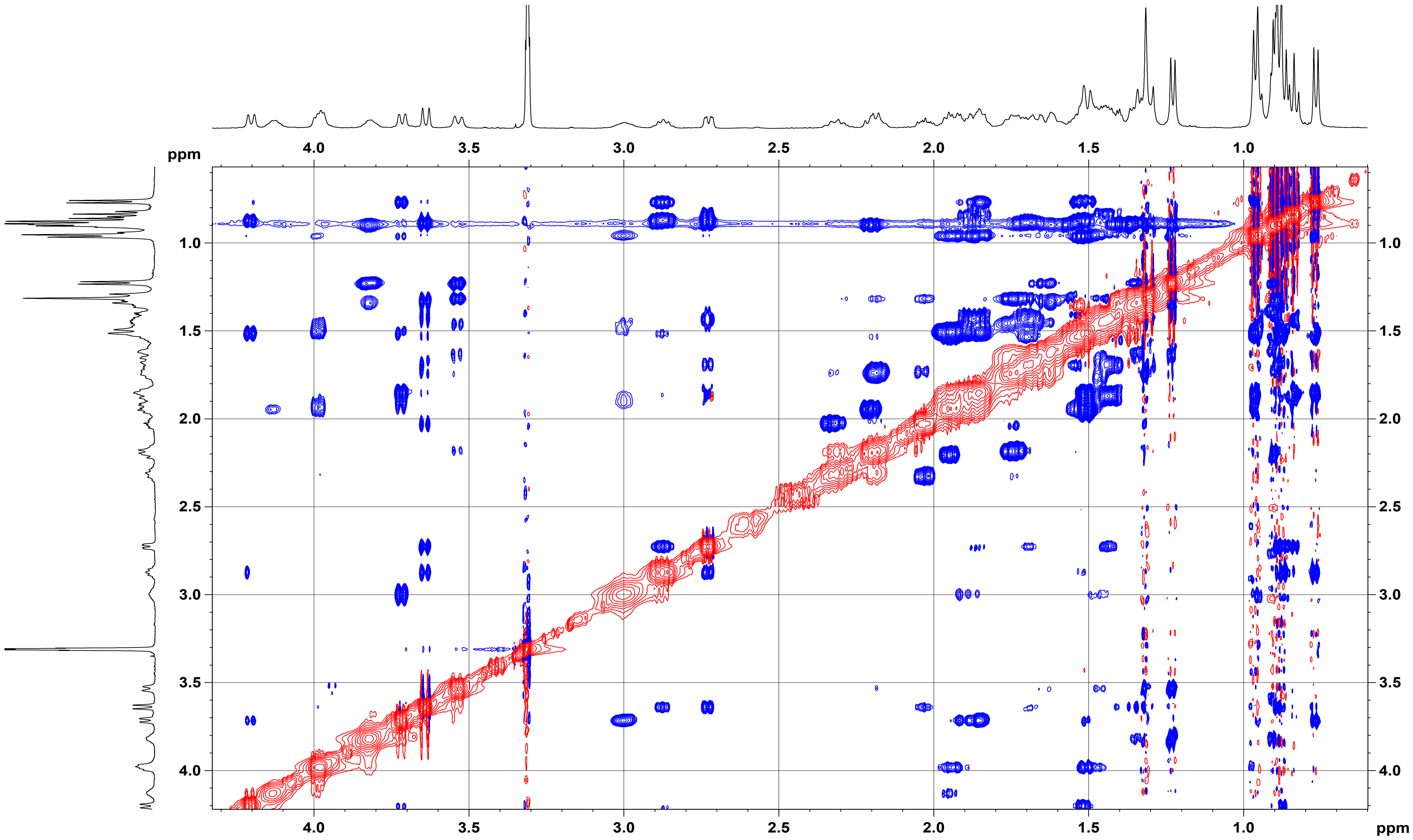

Figure S51. ^1H NMR spectrum of 3. The spectrum was recorded in CD_3CN.

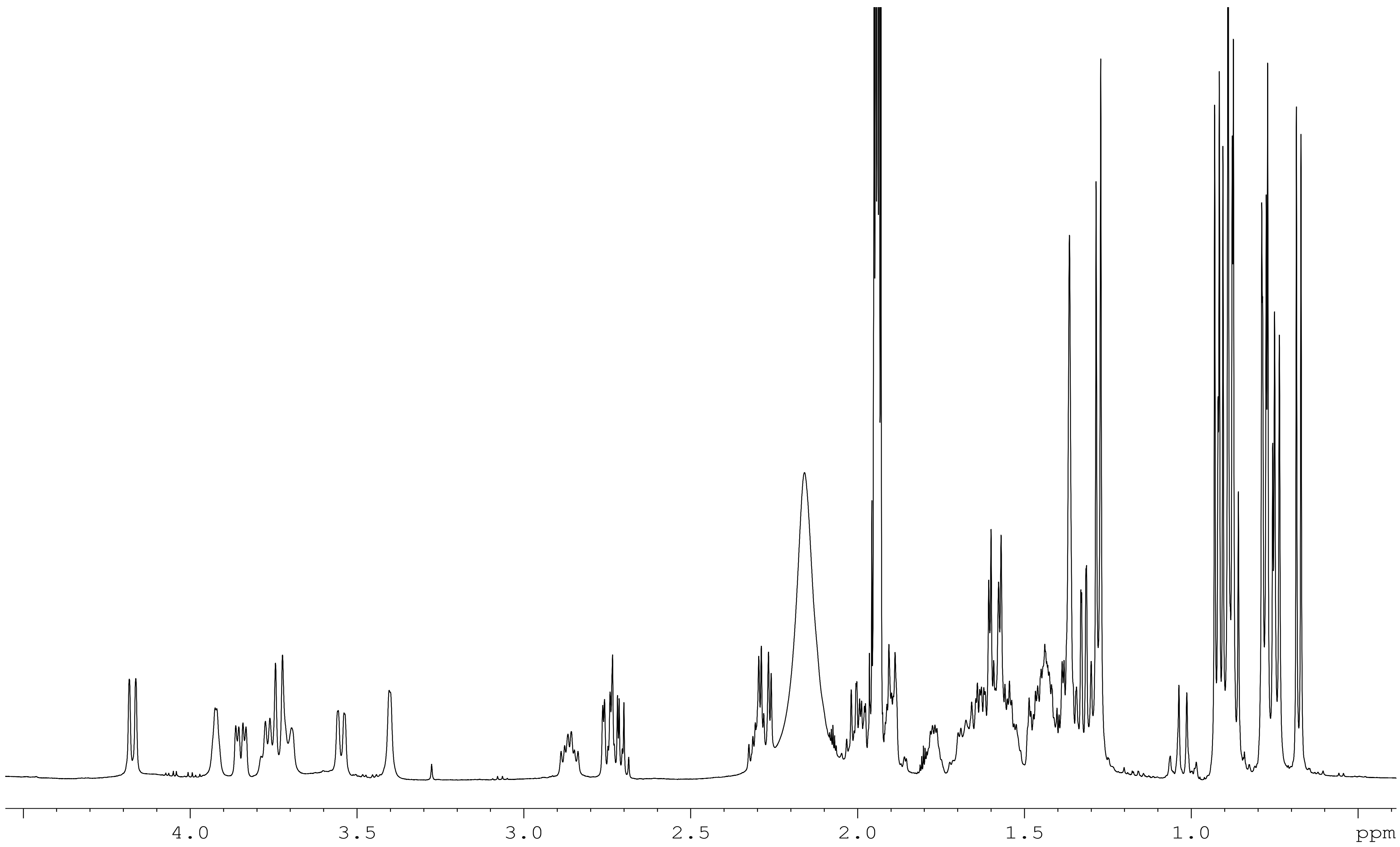

Figure S52. Expansion of the regions between 0.6 ppm and 2.2 ppm, and 2.2 ppm and 4.3 ppm in the ^{1}H NMR spectrum of 3 from Figure S51.

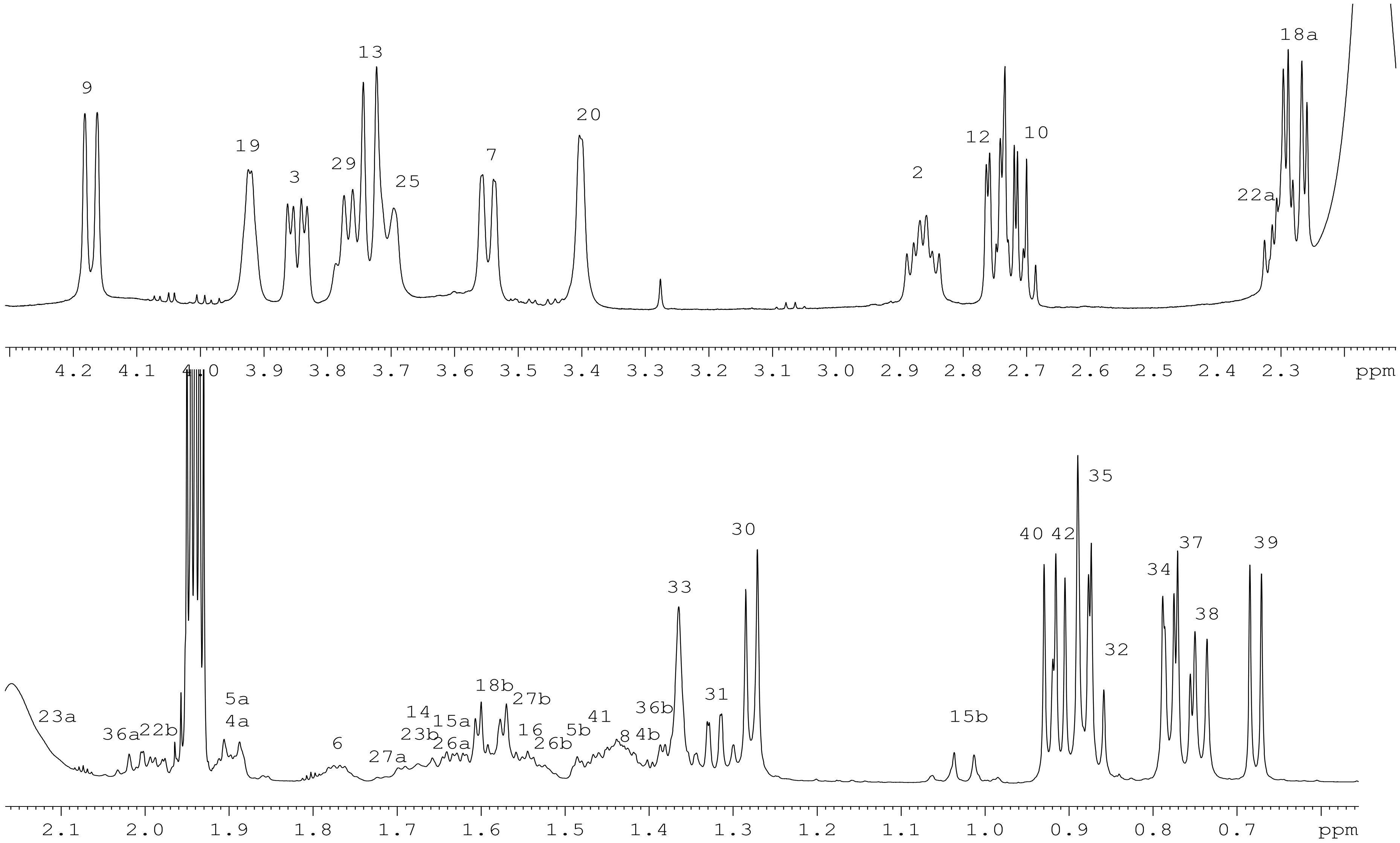

Figure S53. ^{13}C NMR spectrum of 3. The spectrum was recorded in CD_3CN.

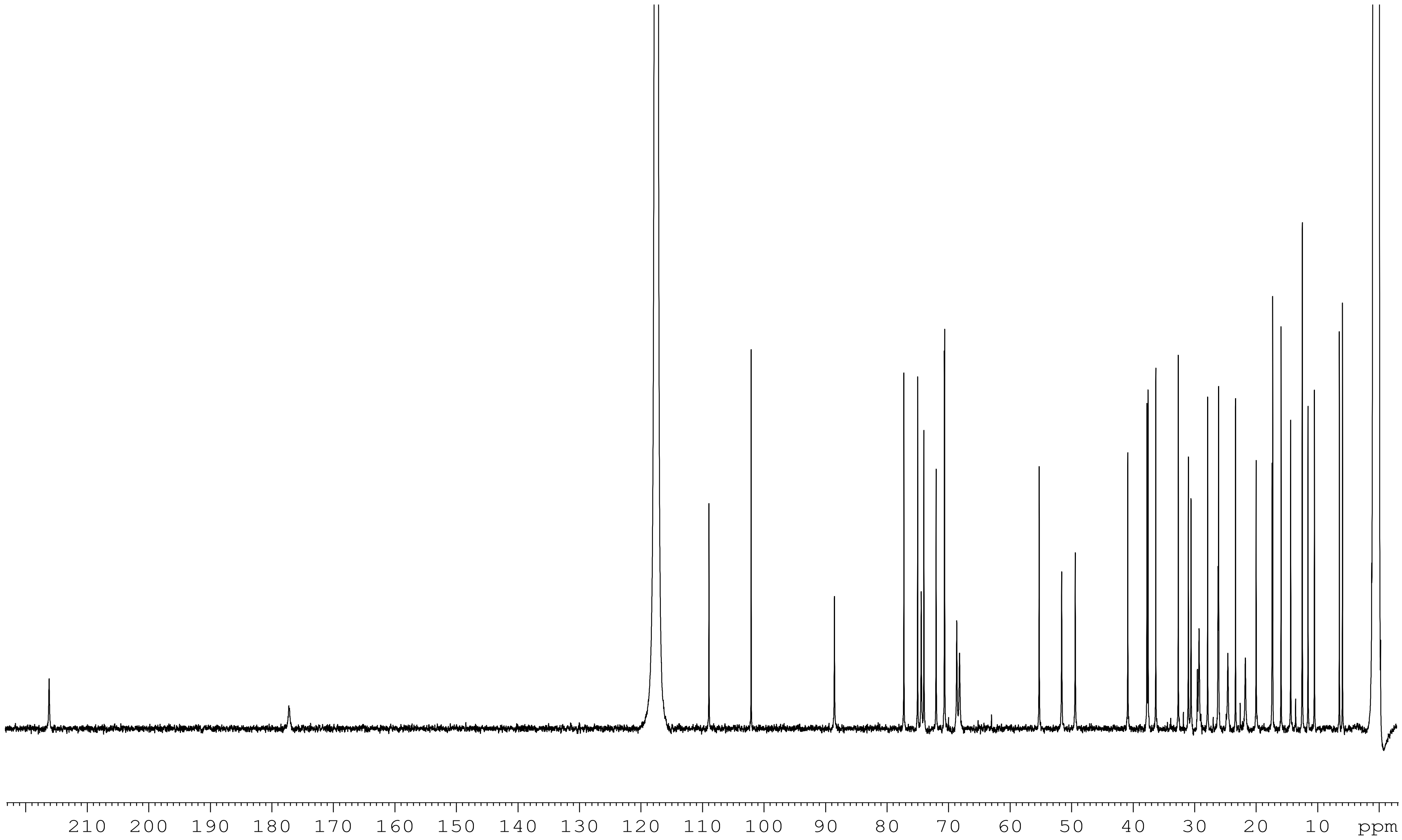

Figure S54. Expansion of the regions between 5 ppm and 43 ppm, and 43 ppm and 220 ppm in the ^{13}C NMR spectrum of 3 from Figure S53.

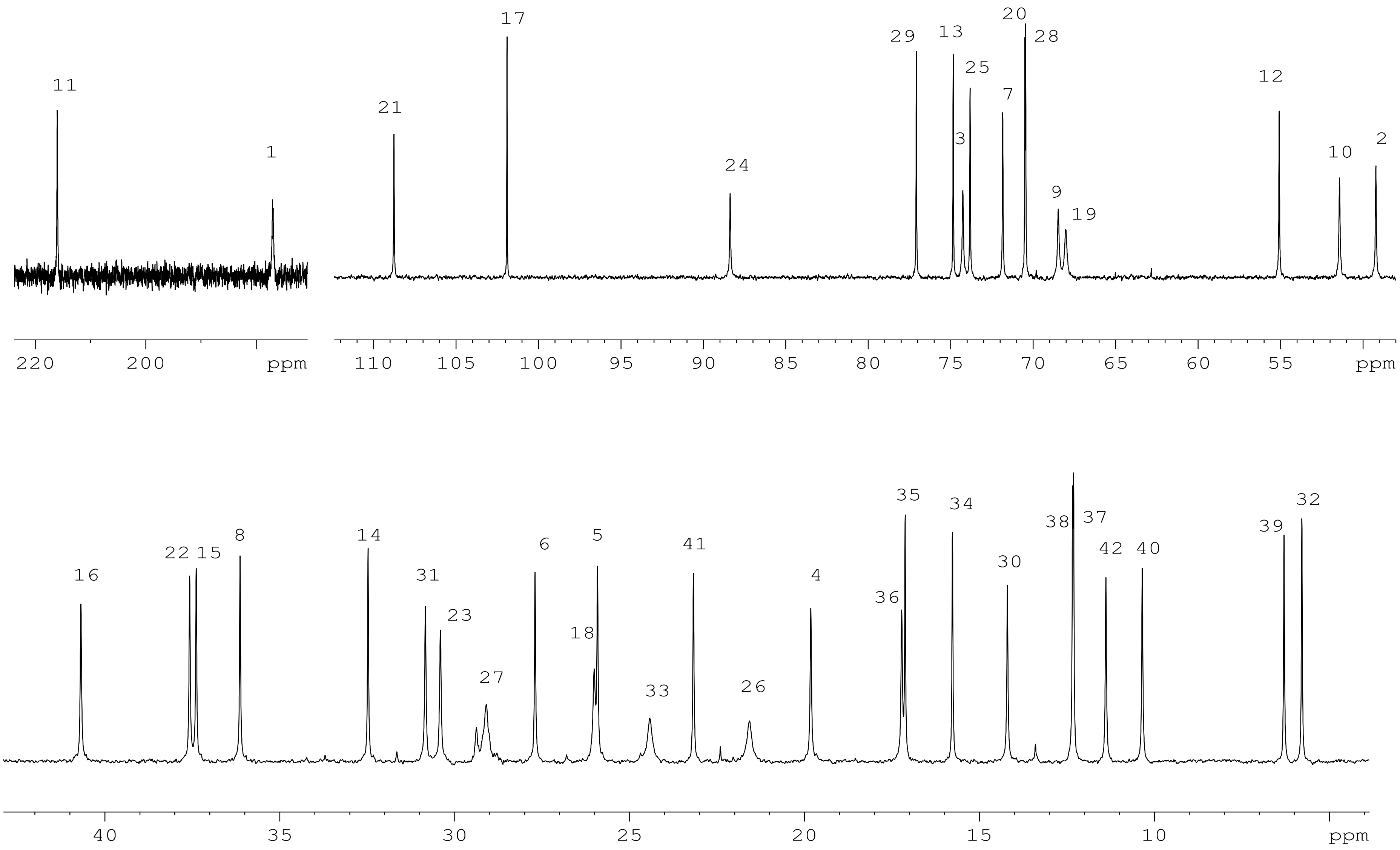

Figure S55. DQF-COSY NMR spectrum of 3. The spectrum was recorded in CD_3CN.

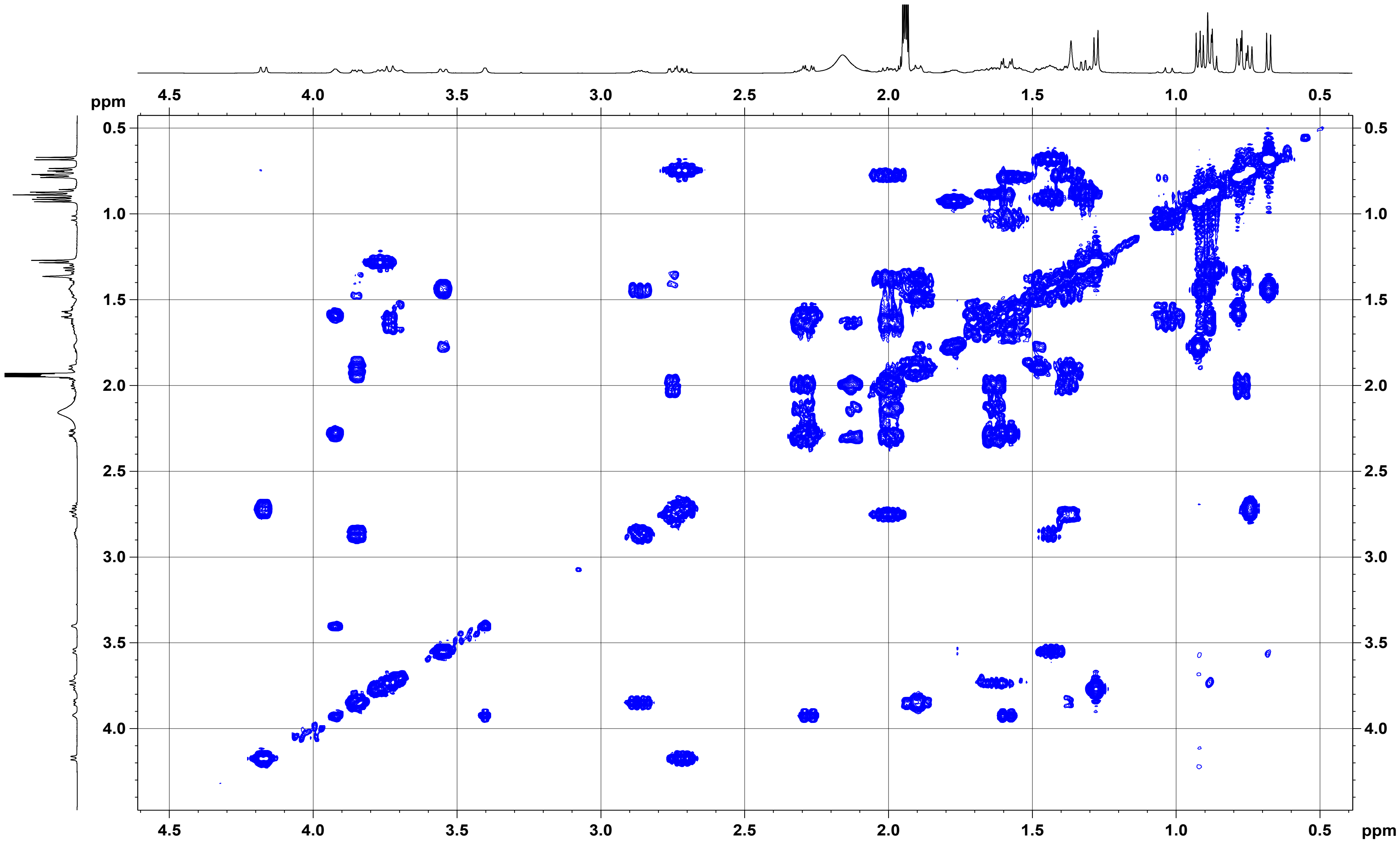

Figure S56. Edited HSQC NMR spectrum of 3. The spectrum was recorded in CD_3CN.

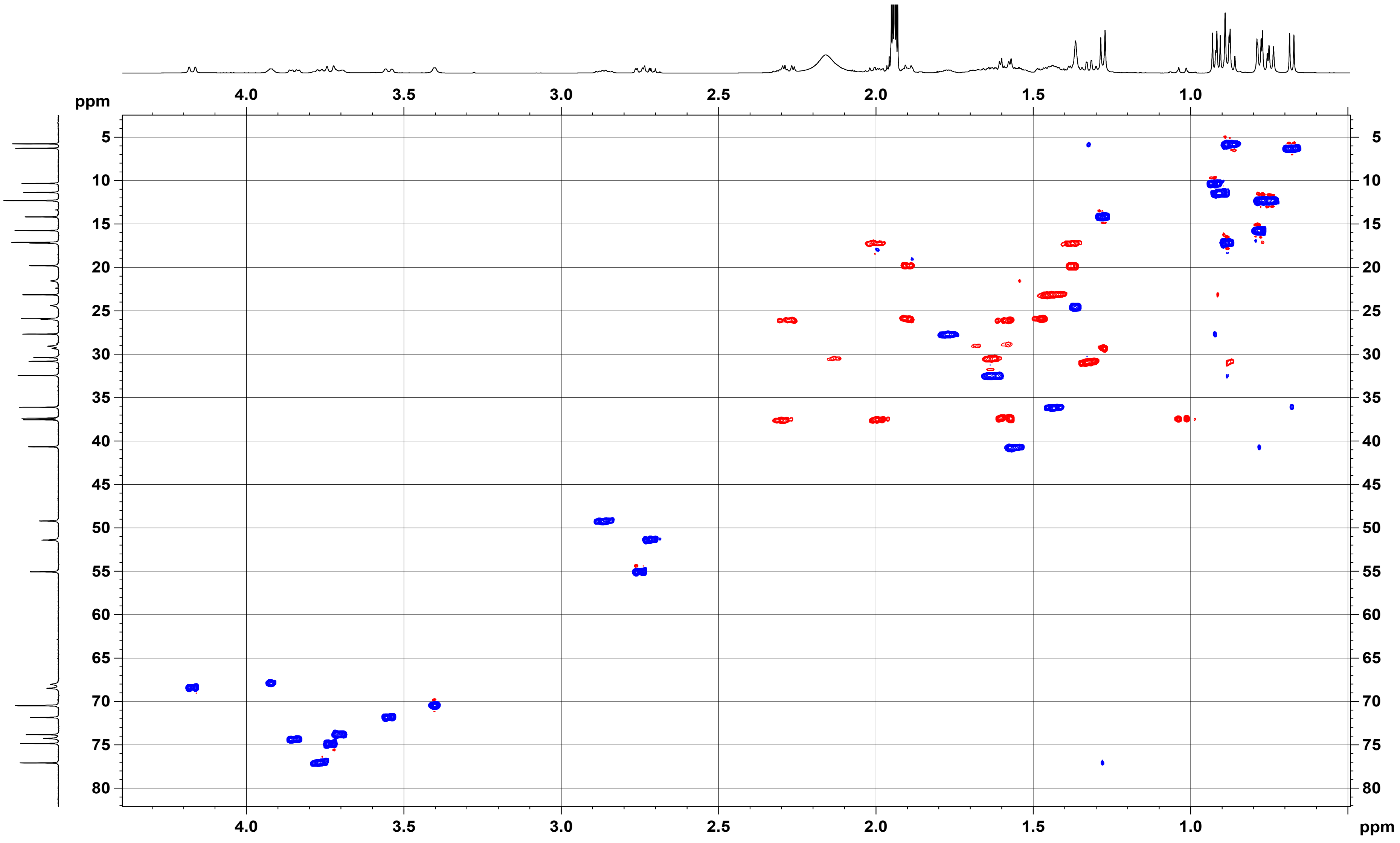

Figure S57. Edited HSQC-TOCSY NMR spectrum of 3. The spectrum was recorded in CD_3CN.

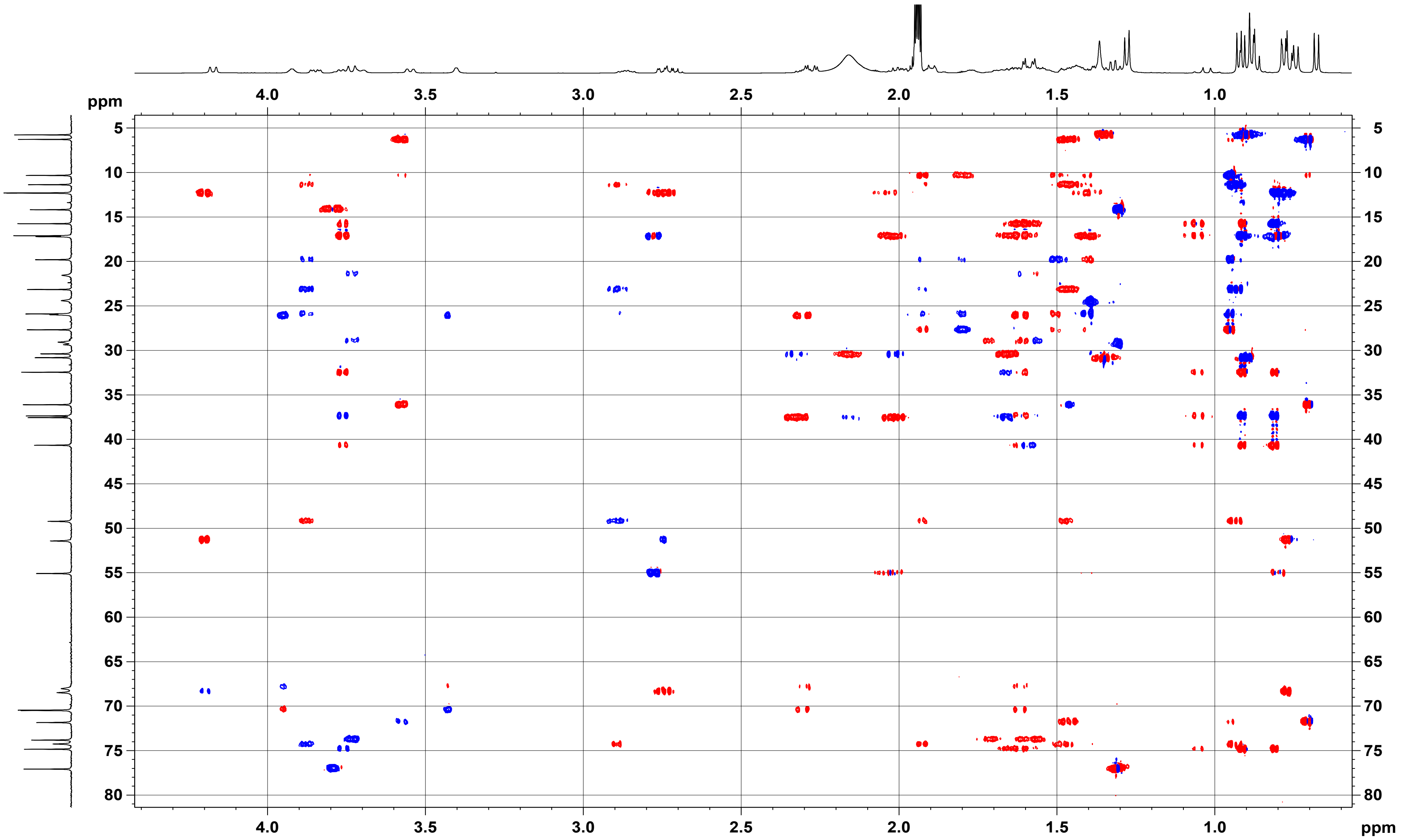

Figure S58. TOCSY NMR spectrum of 3. The spectrum was recorded in CD_3CN.

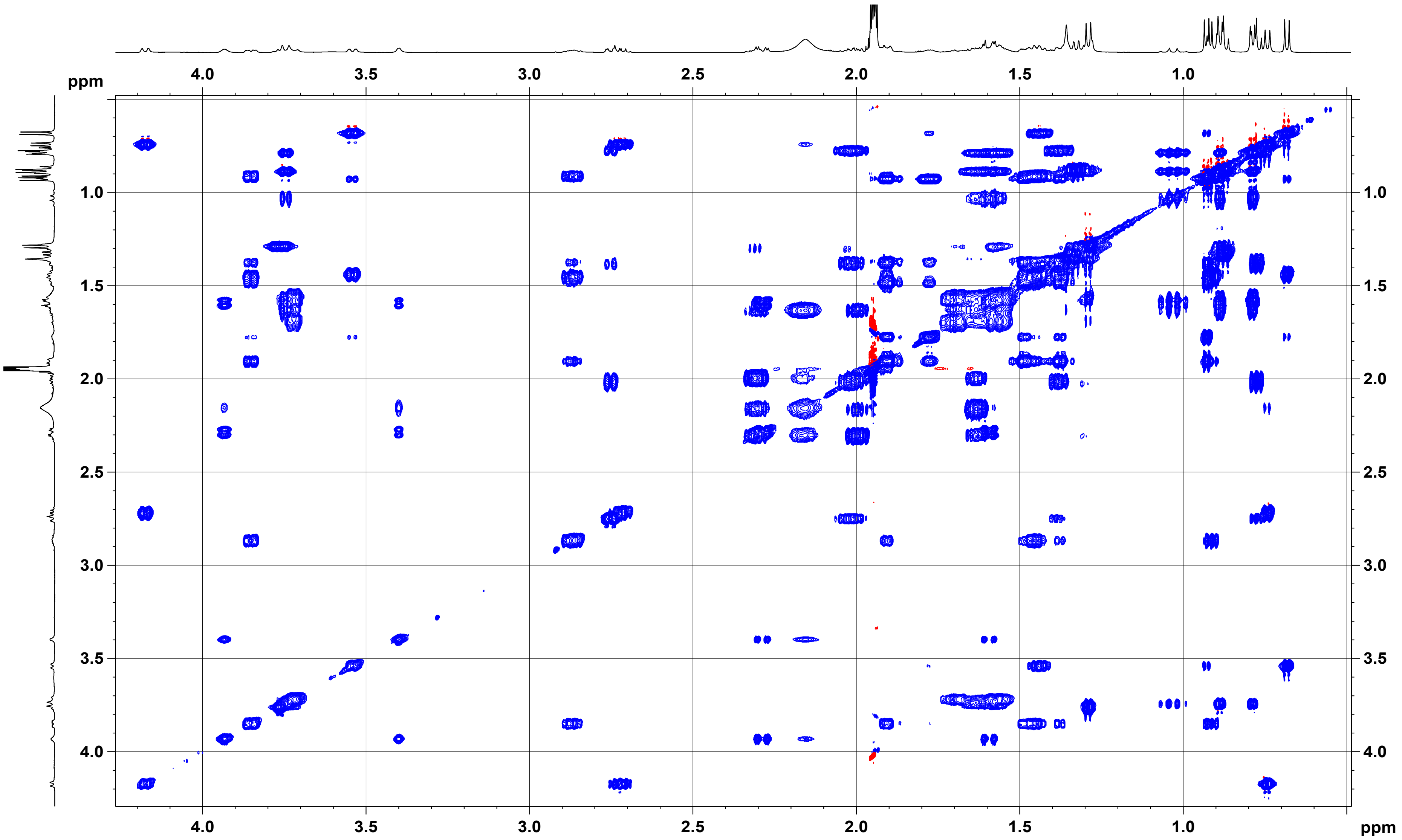

Figure S59. NOESY NMR spectrum of 3. The spectrum was recorded in CD_3CN.

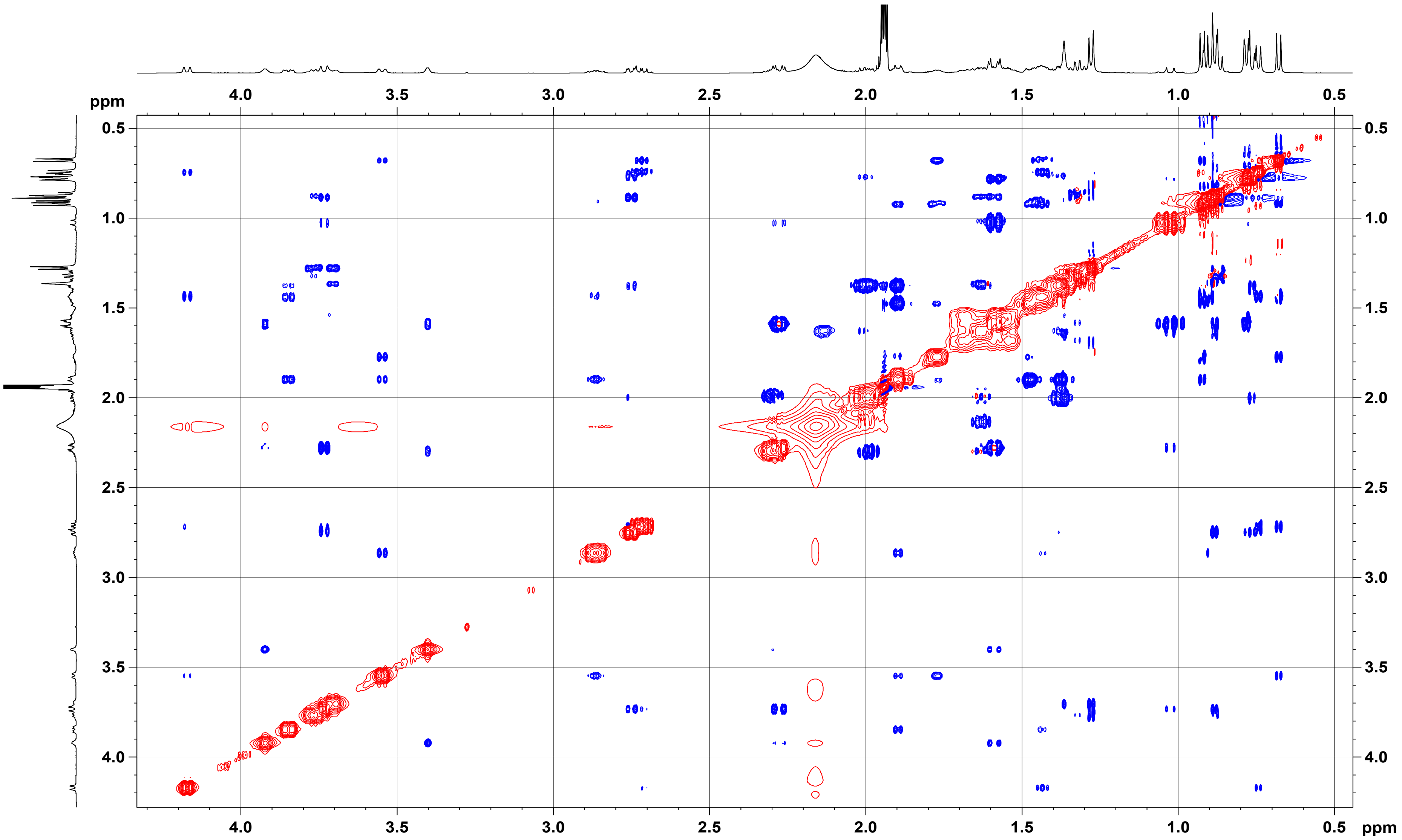

Figure S60. HMBC NMR spectrum of 3. The spectrum was recorded in CD_3CN.

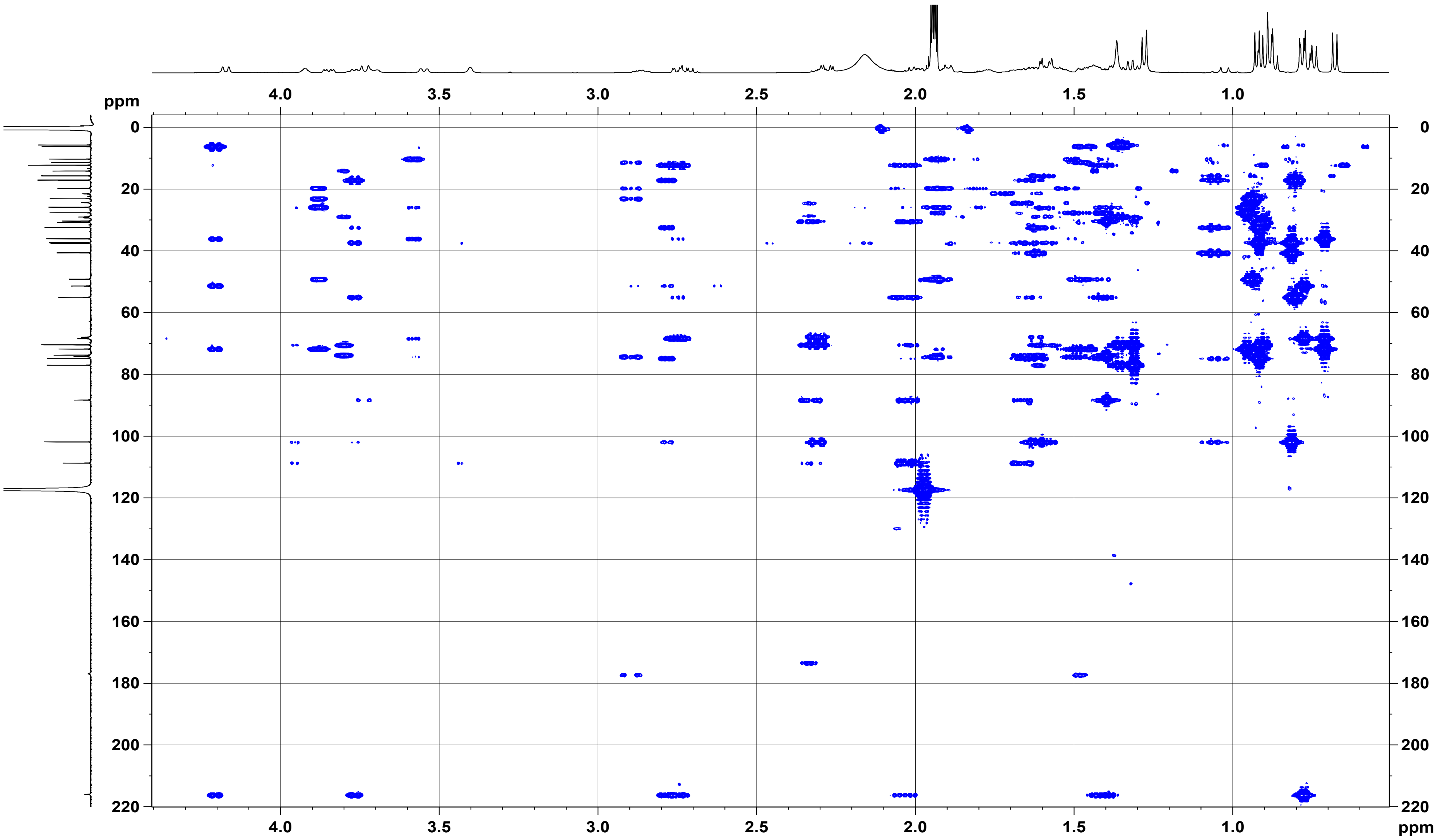

Figure S61. ^1H NMR spectrum of 1. The spectrum was recorded in CD_3CN.

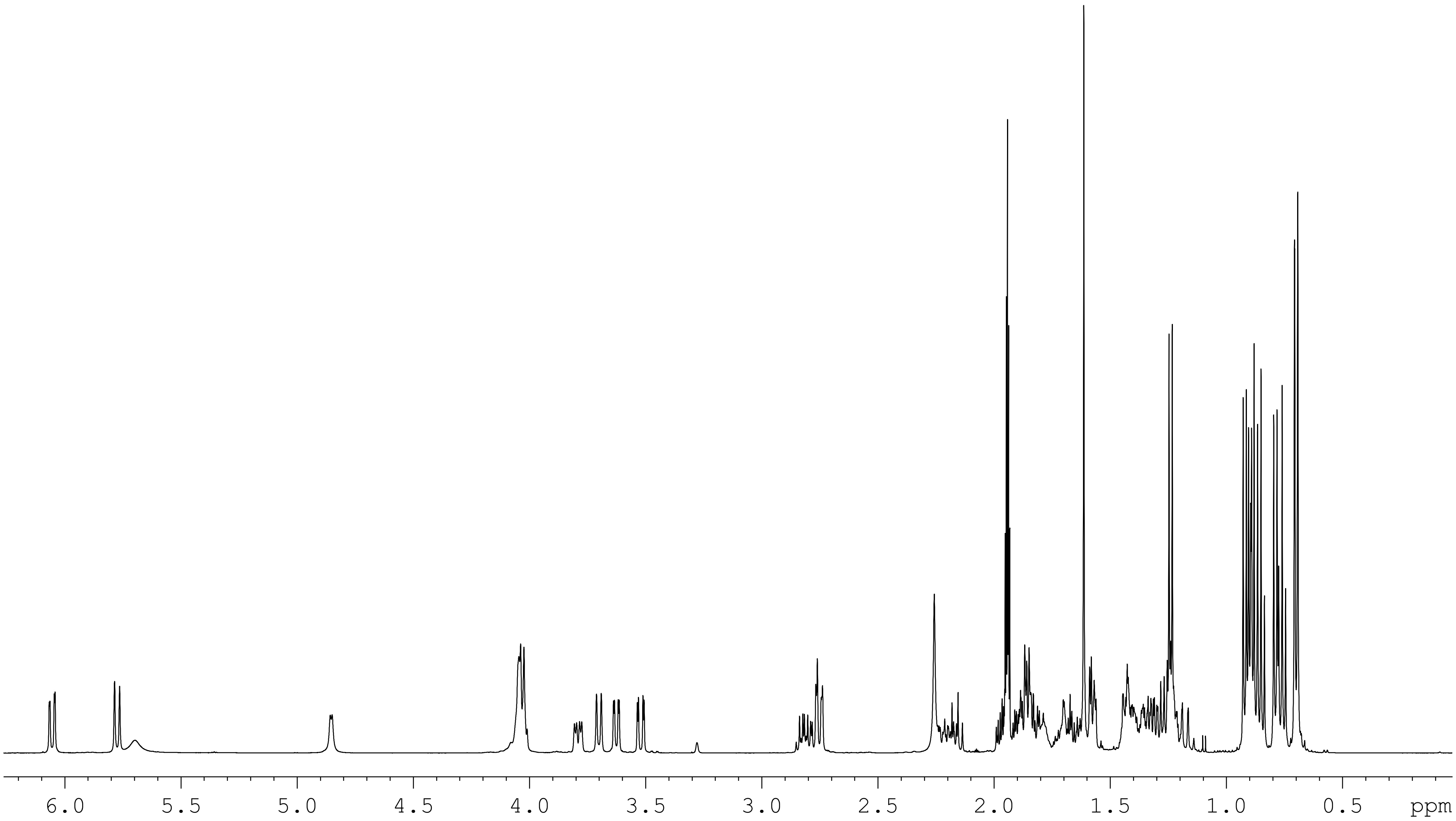

Figure S62. Expansion of the regions between 0.6 ppm and 2.3 ppm, and 2.2 ppm and 6.2 ppm in the ^{1}H NMR spectrum of 1 from Figure S61.

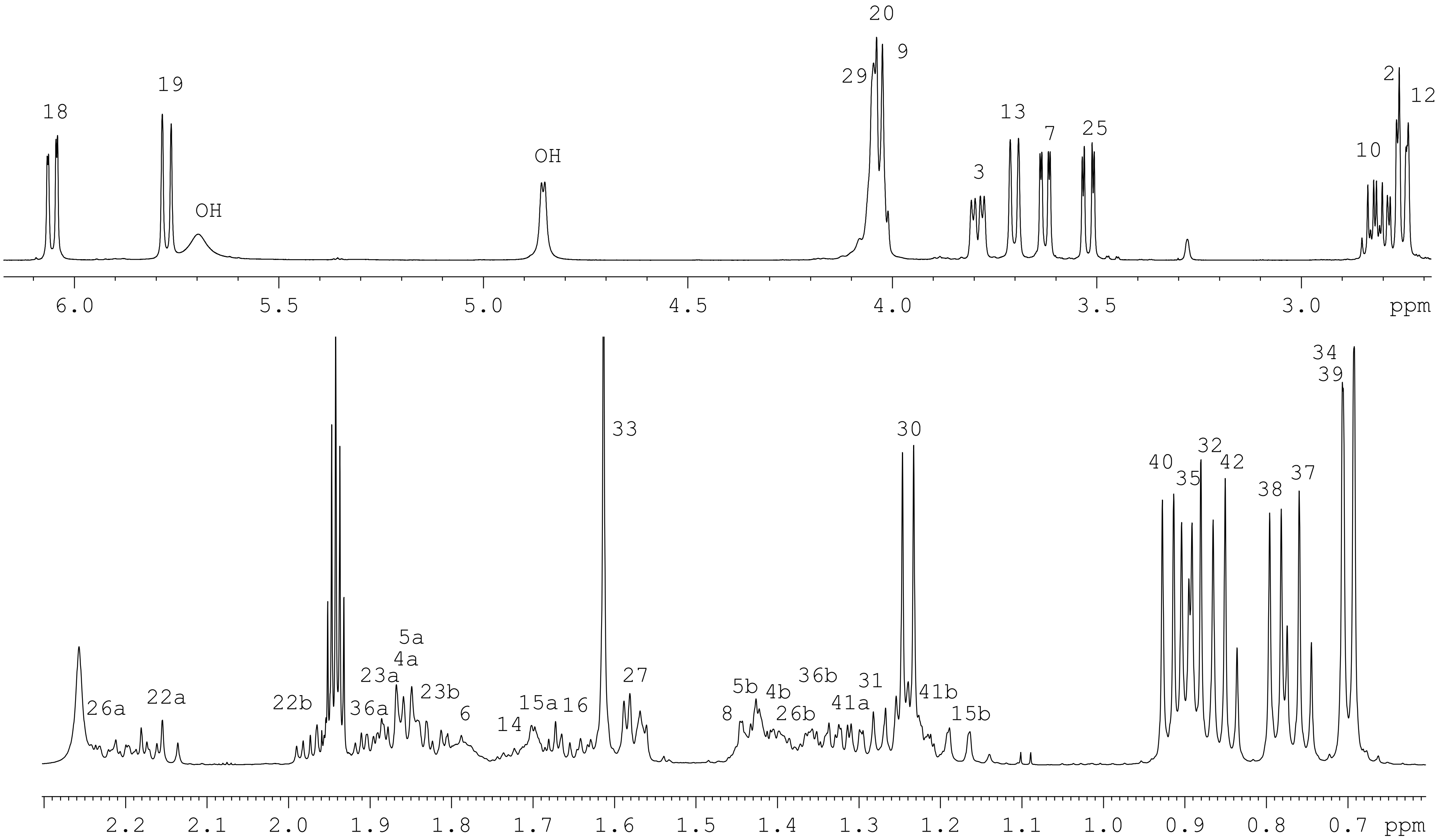

Figure S63. ^{13}C **NMR spectrum of 1. The spectrum was recorded in** CD_3CN**.**

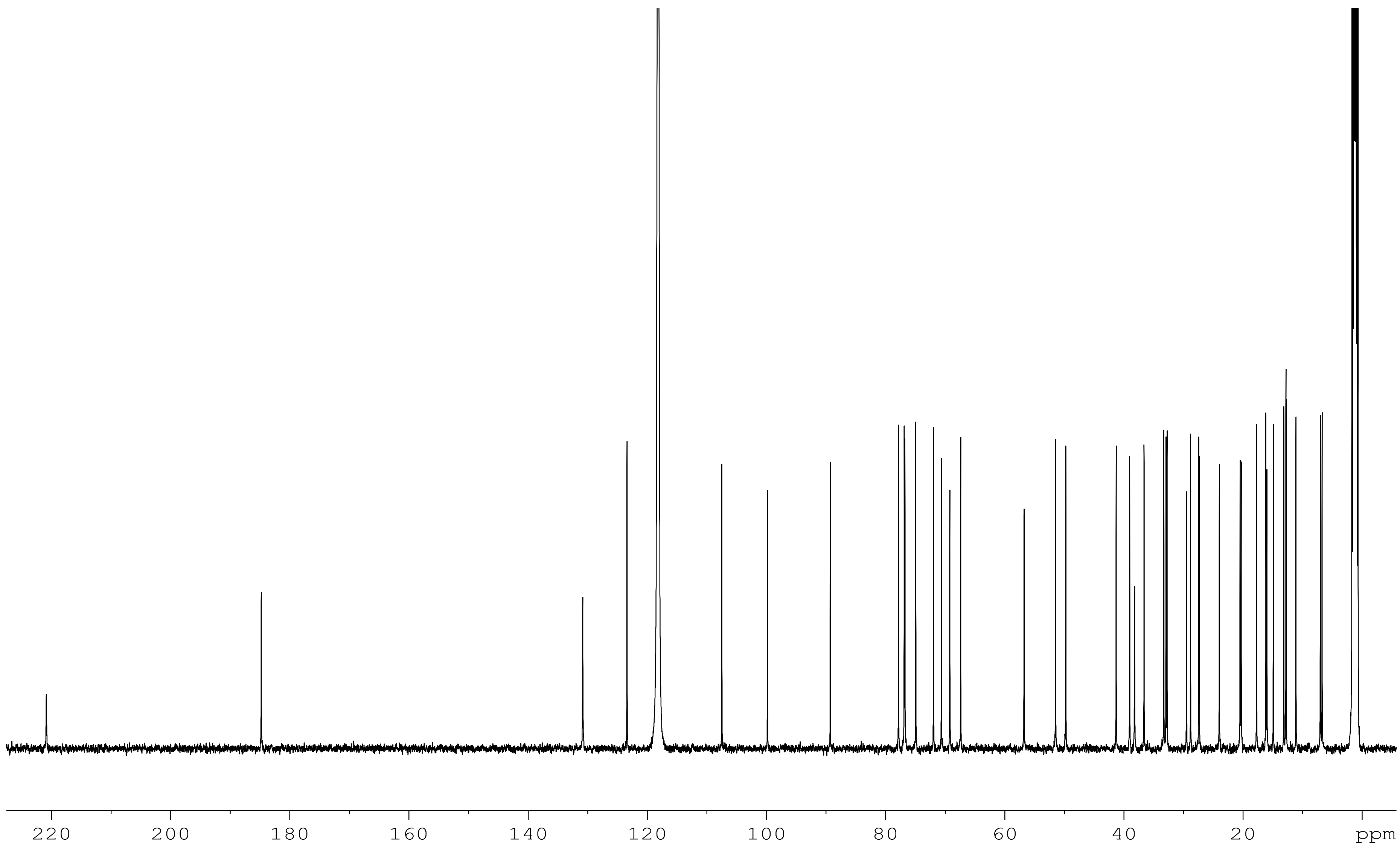

Figure S64. Expansion of the regions between 5 ppm and 60 ppm, and 65 ppm and 230 ppm in the ^{13}C NMR spectrum of 1 from Figure S63.

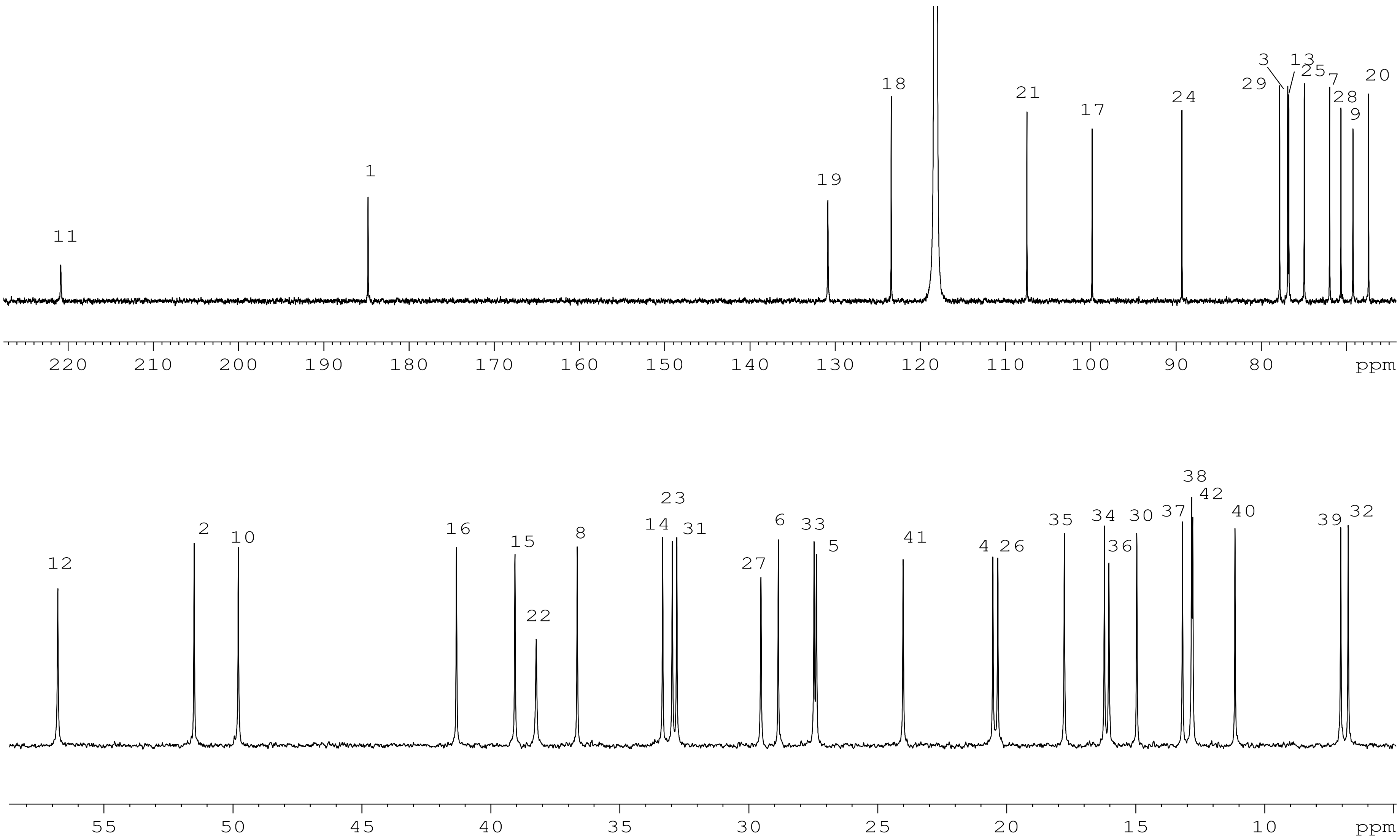

Figure S65. DEPT 135 NMR spectrum of 1. The spectrum was recorded in CD_3CN.

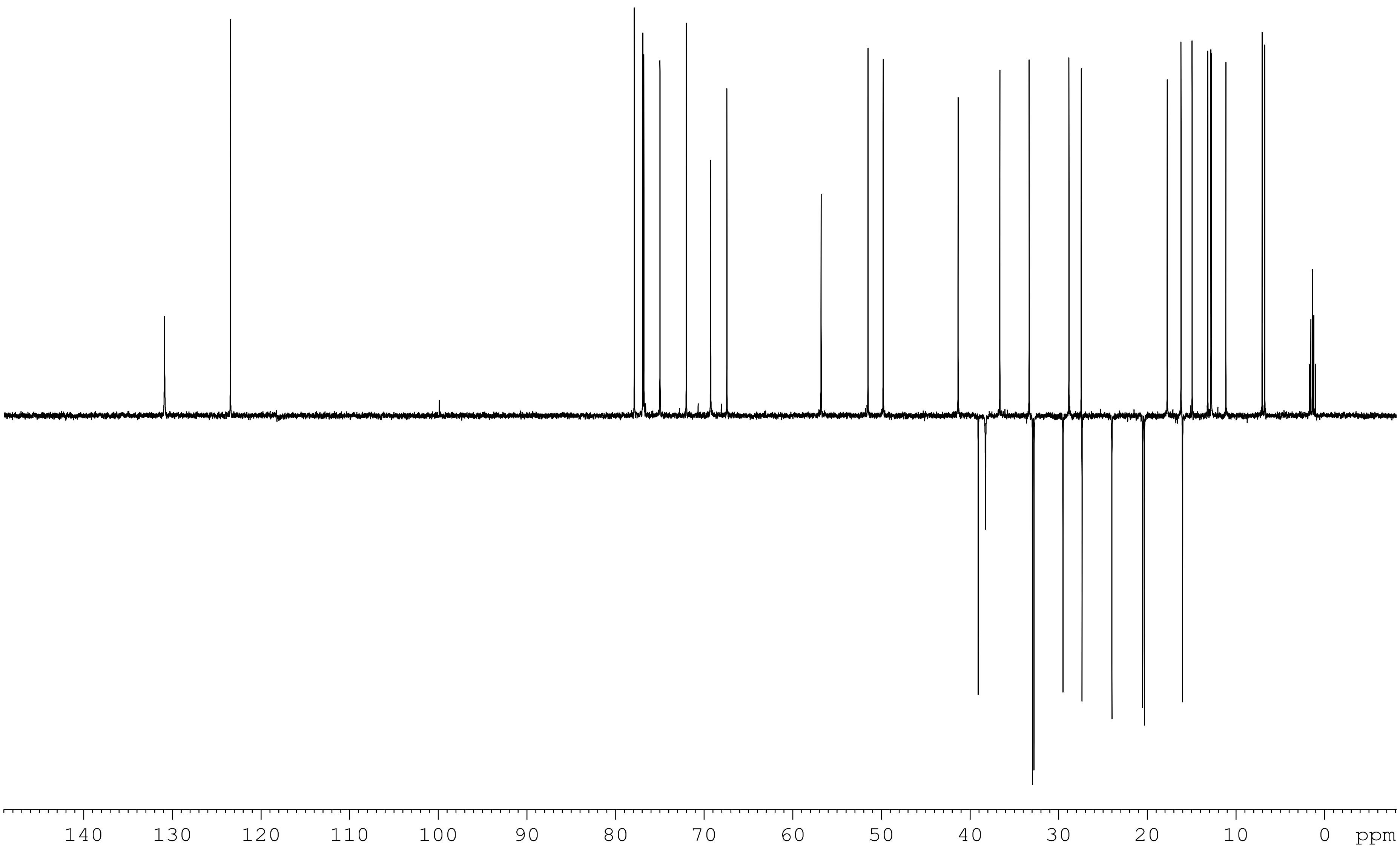

Figure S66. DQF-COSY NMR spectrum of 1. The spectrum was recorded in CD_3CN.

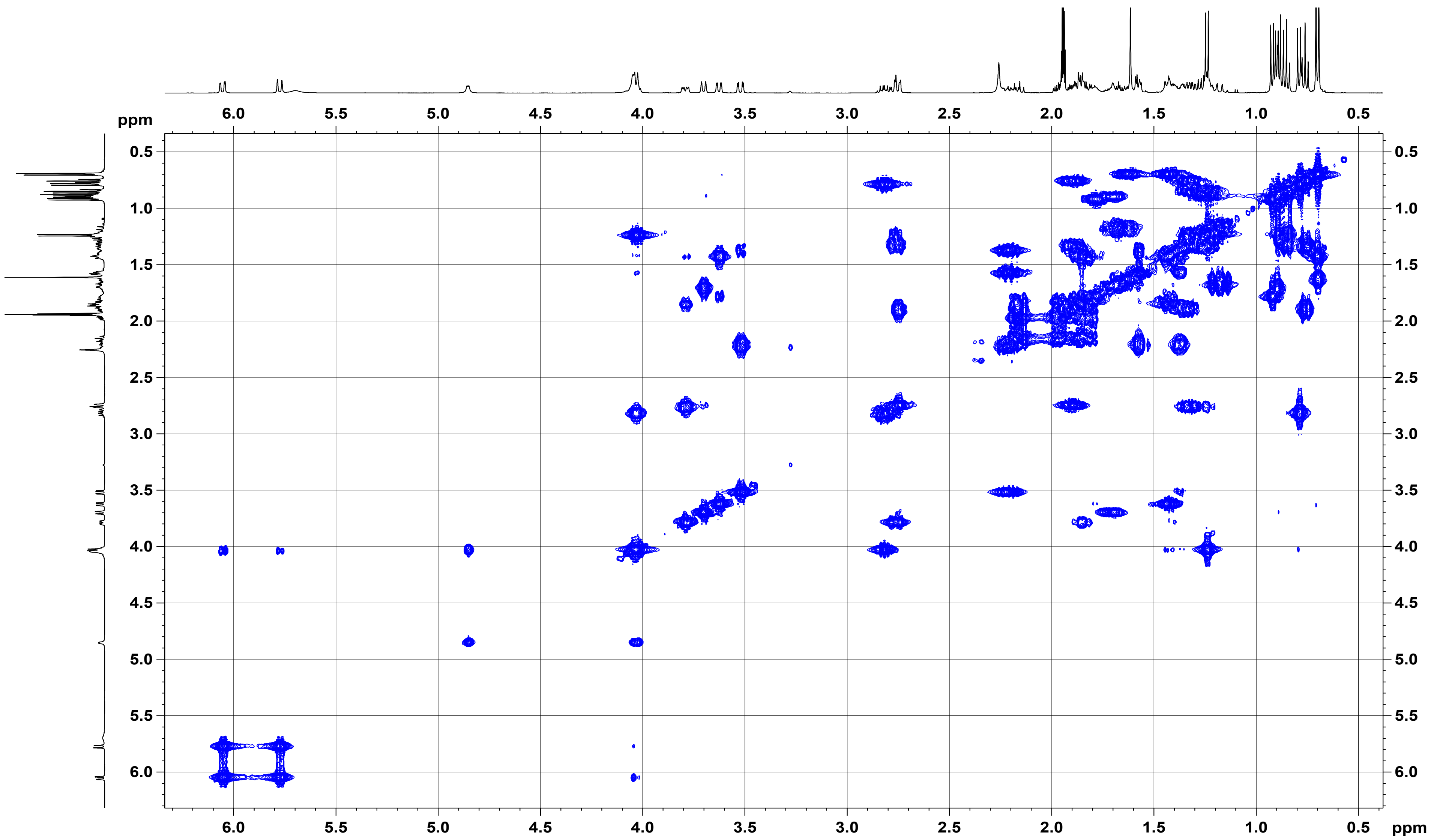

Figure S67. Edited HSQC NMR spectrum of 1. The spectrum was recorded in CD_3CN.

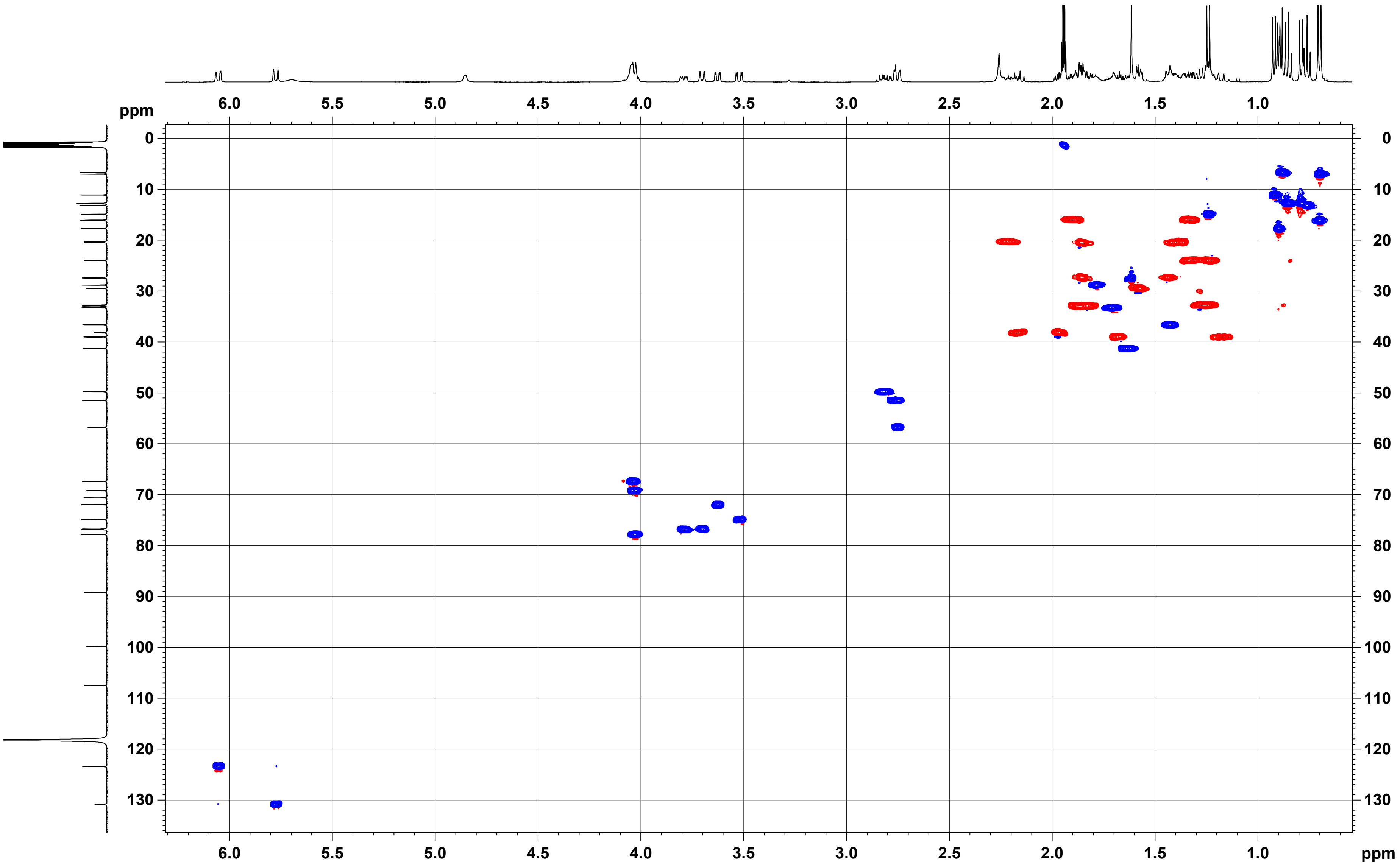

Figure S68. HMBC NMR spectrum of 1. The spectrum was recorded in CD_3CN.

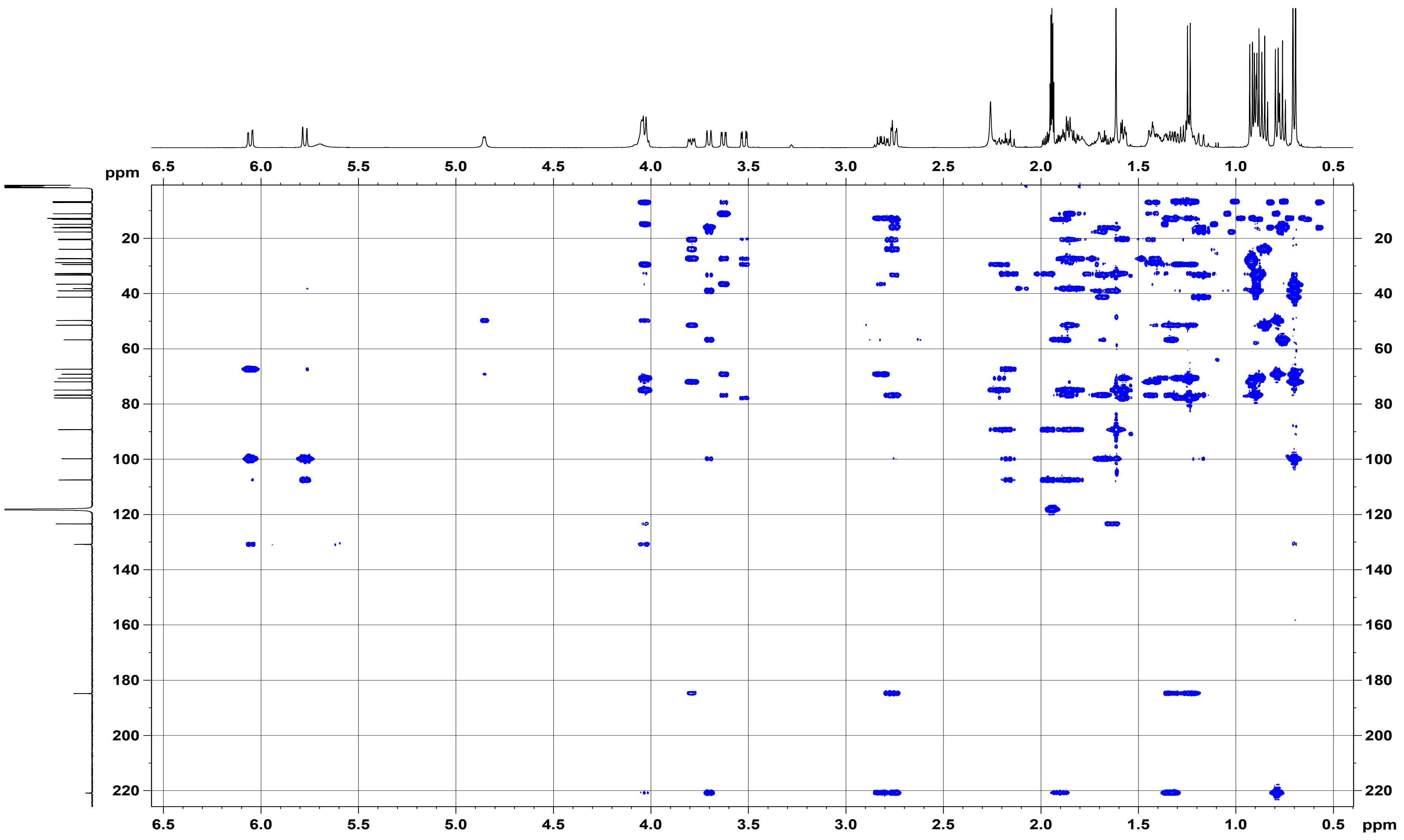

Figure S69. NOESY NMR spectrum of 1. The spectrum was recorded in CD_3CN.

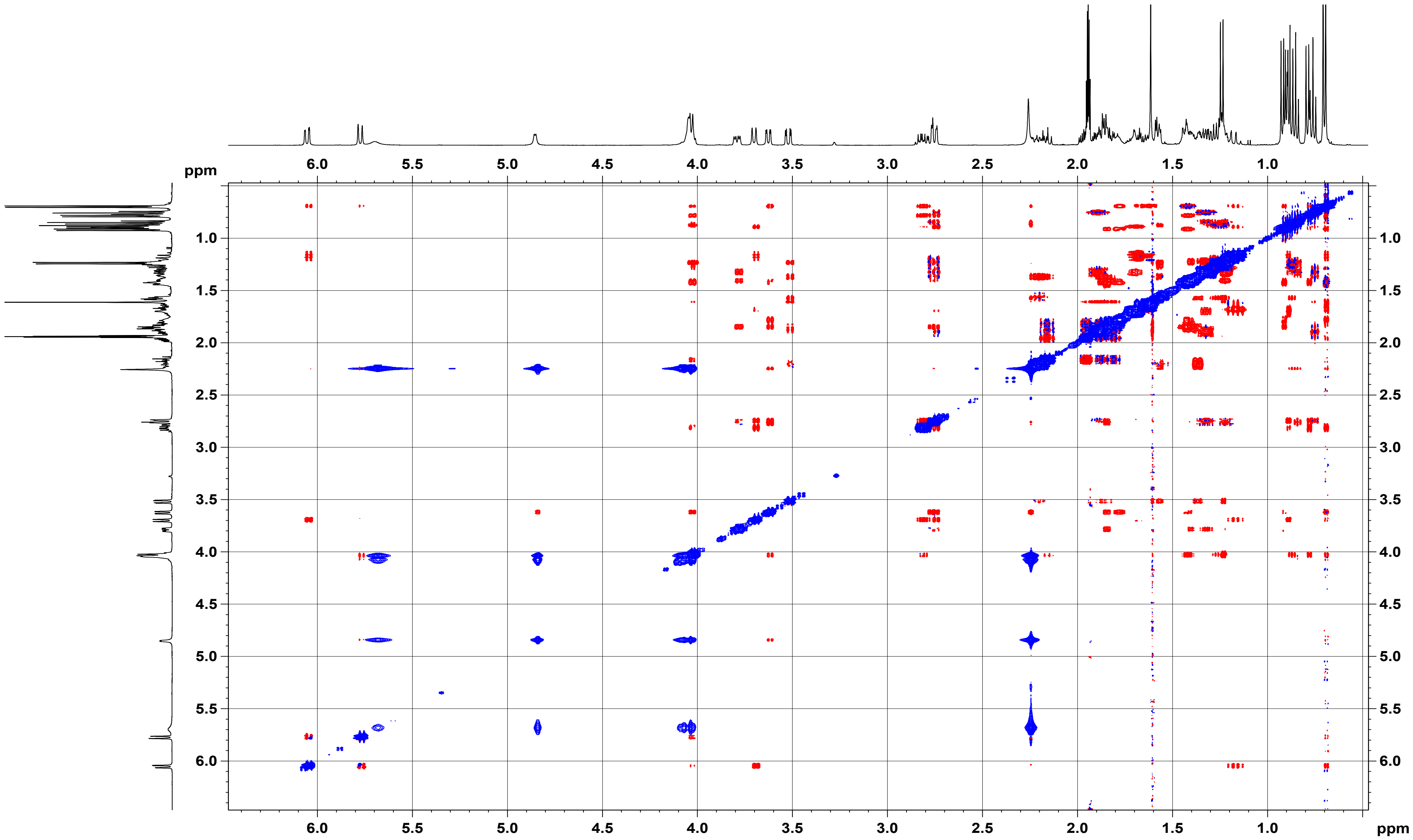

Figure S70. TOCSY NMR spectrum of 1. The spectrum was recorded in CD_3CN.

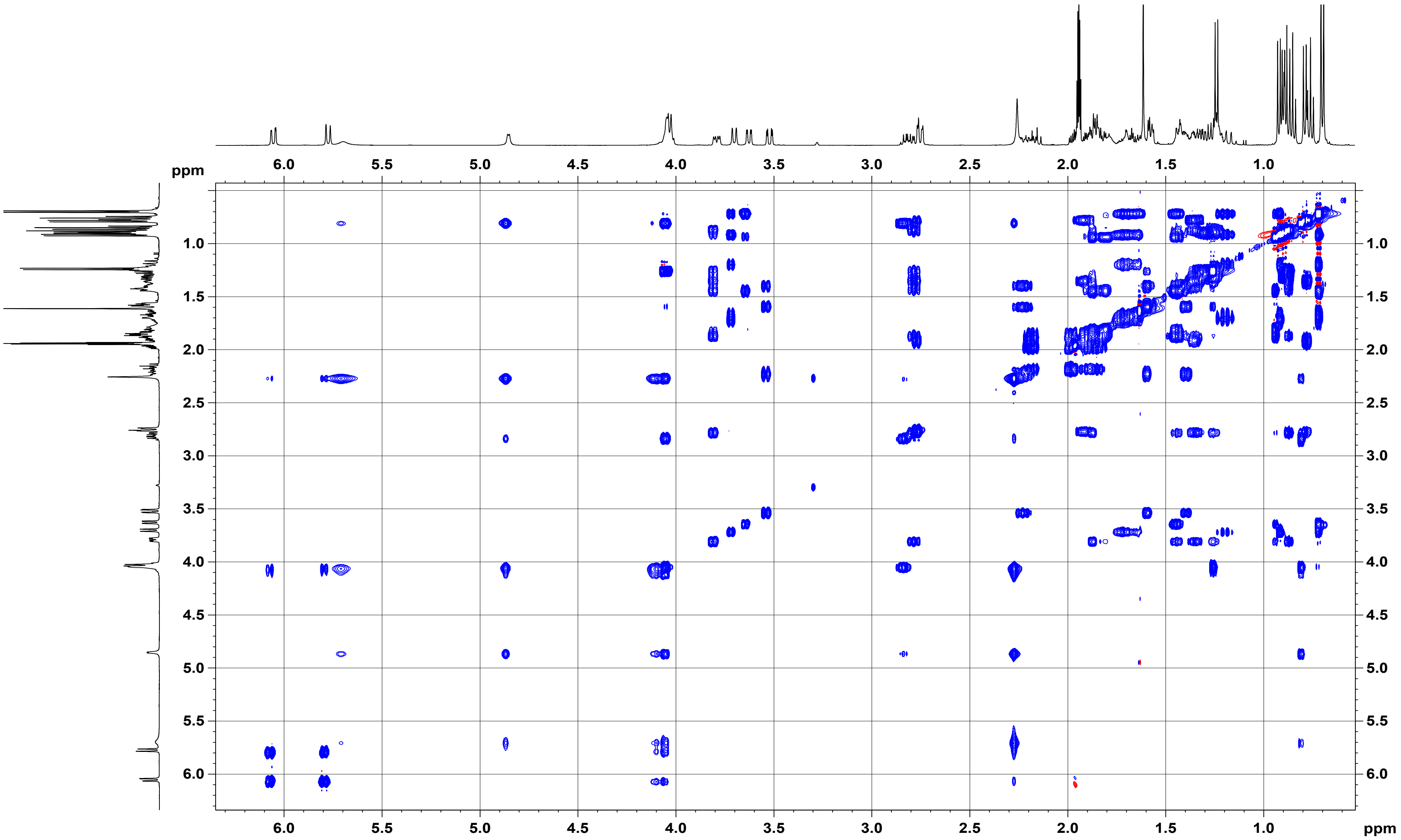

Supplementary References

[1] K. Osoegawa, P. J. Jong, E. Frengen, P. A. Ioannou, *Curr. Protoc. Hum. Genet.* **2001,** *21,* 5.15.1-5.15.33.

[2] T. Kieser, M. J. Bibb, M. J. Buttner, K. F. Chater, D. A. Hopwood, *Practical Streptomyces Genetics*. The John Innes Foundation, Norwich, **2000**.

[3] D. J. Gibson, L. Young, R. Y. Chung, J. C. Venter, C. A. Hutchison, H. O. Smith, *Nat. Methods* **2009,** *6,* 343–345.

[4] A. C. Jones, B. Gust, A. Kulik, L. Heide, M. J. Buttner, M. J. Bibb, *PLoS ONE* **2013,** *8,* e69319.

[5] W. J. Dower, J. F. Miller, C. W. Ragsdale, *Nucleic Acids Res.* **1988,** *16,* 6127–6145.

[6] M. E. Yurkovich, P. A. Tyrakis, H. Hong, Y. Sun, M. Samborskyy, K. Kamiya, P. F. Leadlay, *ChemBioChem* **2011,** *13,* 66–71.

[7] D. J. MacNeil, K. M. Gewain, C. L. Ruby, G. Dezeny, P. H. Gibbons, T. MacNeil, *Gene* **1992,** *111,* 61-68.

[8] J. P. Gomez-Escribano, M. J. Bibb, *Microb. Biotechnol.* **2011,** *4,* 207–215.

[9] Y. Sun, X. He, J. Liang, X. Zhou, Z. Deng, *Appl. Microbiol. Biotechnol.* **2009***, 82,* 303–310.

[10] C. J. Wilkinson, Z. A. Hughes-Thomas, C. J. Martin, I. Böhm, T. Mironenko, M. Deacon, M. Wheatcroft, G. Wirtz, J. Stauton, P. F. Leadlay, *J. Mol. Microbiol. Biotechnol.* **2002,** *4,* 417–426.

[11] B. Gust, G. L. Challis, K. Fowler, T. Kieser, K. F. Chater, *Proc. Natl. Acad. Sci.* **2003,** *100,* 1541–1546.
